# Altered surface mGluR5 dynamics provoke synaptic NMDAR dysfunction and cognitive defects in *Fmr1* knockout mice

**DOI:** 10.1038/s41467-017-01191-2

**Published:** 2017-10-24

**Authors:** Elisabetta Aloisi, Katy Le Corf, Julien Dupuis, Pei Zhang, Melanie Ginger, Virginie Labrousse, Michela Spatuzza, Matthias Georg Haberl, Lara Costa, Ryuichi Shigemoto, Anke Tappe-Theodor, Filippo Drago, Pier Vincenzo Piazza, Christophe Mulle, Laurent Groc, Lucia Ciranna, Maria Vincenza Catania, Andreas Frick

**Affiliations:** 1 INSERM, Neurocentre Magendie, Physiopathologie de la plasticité neuronale, U1215, 33077 Bordeaux, cedex France; 2 University of Bordeaux, Neurocentre Magendie, Physiopathologie de la plasticité neuronale, U1215, 33077 Bordeaux, cedex France; 3 Interdisciplinary Institute for Neuroscience, IINS-CNRS, UMR 5297, University of Bordeaux, 33077 Bordeaux, cedex France; 4 University of Bordeaux, Interdisciplinary Institute for Neuroscience, UMR 5297, 33077 Bordeaux, cedex France; 5 Institute of Neurological Sciences, National Research Council, ISN-CNR, 95126 Catania, Italy; 6 Department of Clinical and Experimental Medicine, University of Messina, 98125 Messina, Italy; 7 IST Austria, Klosterneuburg, 3400 Austria; 8 Institute for Pharmacology, University of Heidelberg, Im Neuenheimer Feld 366, 69120 Heidelberg, Germany; 9 Department of Biomedical and Biotechnological Sciences, University of Catania, 95123 Catania, Italy; 10 Oasi Maria SS Institute for Research on Mental Retardation and Brain Aging (IRCCS), 94018 Troina (EN), Italy

## Abstract

Metabotropic glutamate receptor subtype 5 (mGluR5) is crucially implicated in the pathophysiology of Fragile X Syndrome (FXS); however, its dysfunction at the sub-cellular level, and related synaptic and cognitive phenotypes are unexplored. Here, we probed the consequences of mGluR5/Homer scaffold disruption for mGluR5 cell-surface mobility, synaptic N-methyl-D-aspartate receptor (NMDAR) function, and behavioral phenotypes in the second-generation *Fmr1* knockout (KO) mouse. Using single-molecule tracking, we found that mGluR5 was significantly more mobile at synapses in hippocampal *Fmr1* KO neurons, causing an increased synaptic surface co-clustering of mGluR5 and NMDAR. This correlated with a reduced amplitude of synaptic NMDAR currents, a lack of their mGluR5-activated long-term depression, and NMDAR/hippocampus dependent cognitive deficits. These synaptic and behavioral phenomena were reversed by knocking down Homer1a in *Fmr1* KO mice. Our study provides a mechanistic link between changes of mGluR5 dynamics and pathological phenotypes of FXS, unveiling novel targets for mGluR5-based therapeutics.

## Abstract (web summary)

Dysfunction of mGluR5 has been implicated in Fragile X syndrome. Here, using a single-molecule tracking technique, the authors found an increased lateral mobility of mGluR5 at the synaptic site in *Fmr1* KO hippocampal neurons, leading to abnormal NMDAR-mediated synaptic plasticity and cognitive deficits.

## Introduction

Fragile X syndrome (FXS) is the most common form of inherited intellectual disability and best-known cause of autism^1^. In most cases FXS is caused by transcriptional silencing of the *FMR1* gene and the ensuing lack of encoded Fragile X Mental Retardation Protein (FMRP) (reviewed in ref. ^2^), an RNA-binding protein that regulates translation and trafficking of its interacting mRNAs in dendrites and axons (reviewed in ref. ^3^). During the last decade numerous FMRP target mRNAs have been identified^4–7^. In contrast, how changes in the expression of their protein products contribute to different features of FXS pathology remains to be elucidated in detail (reviewed in refs. ^8–11^). Studies from the *Fmr1* knockout (KO) mouse model of FXS provide compelling evidence that an increased expression of a subset of synaptic proteins—and subsequent alteration in synaptic plasticity—contribute to numerous cognitive phenotypes of this disorder (reviewed in ref. ^12^). In particular, exaggerated group-I metabotropic glutamate receptor subtype 5 (mGluR5)/protein synthesis-dependent hippocampal long-term depression (LTD) of α-amino-3-hydroxy-5-methyl-4-isoxazole propionic acid receptor (AMPAR) currents is a hallmark feature of FXS^13^. This seminal finding forms the basis of the mGluR theory of FXS^14^. In support of this theory, correction of the aberrant mGluR5 signaling through either pharmacological or genetic means, leads to the rescue of a number of disease phenotypes (reviewed in ref. ^15^).

Although much work has focused on the protein synthesis-dependent functional consequences of inappropriate mGluR5 activation, other findings suggest that the intrinsic properties and signal transduction mechanisms of mGluR5 might also be altered in FXS (reviewed in ref. ^16^). Indeed, previous work has demonstrated that the interaction between long Homer proteins and mGluR5 is reduced in the absence of FMRP^17^, likely contributing to an altered mGluR5-mediated signaling in *Fmr1* KO mice^18, 19^. The consequences of altered protein interactions for receptor dynamics at synapses, however, remain to be investigated.

The dynamic movement of synaptic components has emerged as a key feature of synaptic transmission and plasticity (reviewed in refs. ^20, 21^). Indeed, receptors on the neuronal surface constantly switch between mobile and immobile states, driven by thermal agitation and reversible binding to stable elements such as scaffolding proteins, cytoskeletal anchoring slots or extracellular anchors (reviewed in ref. ^22^). The mobility of receptors within the membrane may promote their interactions with other synaptic receptors (reviewed in ref. ^22^), and its alteration might also correlate with pathophysiological states, as recently suggested for neurodegenerative disorders^23, 24^. Thus, an understanding of the dynamics of receptors at *Fmr1* KO synapses may provide novel insights into the mechanisms underlying the synaptic pathology in FXS.

Homer proteins are a family of post-synaptic density (PSD) scaffolding proteins responsible for the link between mGluR5 and other PSD proteins^25^. Both long (Homer1b/c, Homer2, and Homer3, here collectively referred to as Homer) and short (Homer1a) isoforms have been identified. The long Homer isoforms are constitutively expressed, multimerize, and link mGluR5 to signaling pathways within the PSD (reviewed in ref. ^26^). Homer1a, on the other hand, is an immediate early gene inducible by synaptic activity, which functions as a dominant negative regulator of group-I mGluR signaling by disrupting the binding between mGluR5 and Homer^27, 28^. Interestingly, mGluR5 and NMDA receptor (NMDAR) co-assemble in the same Homer-containing PSD complex^25, 29^. In the presence of Homer1a, the multimeric mGluR5/Homer complex is disrupted, permitting direct physical and functional interactions between NMDAR and mGluR5 and promoting mGluR5-mediated inhibition of NMDAR currents^30, 31^.

Here we explored the dynamics of mGluR5 at hippocampal synapses and the consequences of a disrupted interaction with Homer proteins for NMDAR function and plasticity, as well as for related cognitive deficits in *Fmr1* KO mice. We addressed this question using a powerful combination of high-resolution single-molecule tracking, electrophysiological and knockdown approaches in hippocampal neurons from wild type (WT) and *Fmr1* KO mice, together with behavioral analysis. The majority of these experiments were performed using the second-generation *Fmr1* KO mouse line, which lacks both *Fmr1* mRNA and FMRP^32^. Certain electrophysiological experiments were performed both in the second-generation and first-generation^33^ mutants, demonstrating good comparability between these models. We found that the lateral mobility of mGluR5 was increased specifically at the synaptic sites in *Fmr1* KO hippocampal neurons and correlated with an increased synaptic confinement and co-clustering of mGluR5 and NMDAR, likely resulting from the mGluR5/Homer disruption. This led us to investigate changes in synaptic NMDAR currents and their long-term depression following mGluR5 activation. These synaptic phenomena were recapitulated in WT neurons by a peptide-based approach that disrupted the mGluR5/Homer scaffold. Importantly, we found that restoring this mGluR5/Homer interaction by reducing the expression of Homer1a in the hippocampus rescued abnormal NMDAR function and plasticity as well as cognitive deficits in *Fmr1* KO mice. Our data highlights the importance of altered mGluR5 dynamics for the pathophysiology of FXS, corroborating the view that the regulation of the interaction of mGluR5 with long Homer isoforms represents a promising therapeutic target for FXS.

## Results

### Exaggerated synaptic mobility of mGluR5 in *Fmr1* KO neurons

**Fig. 1:**
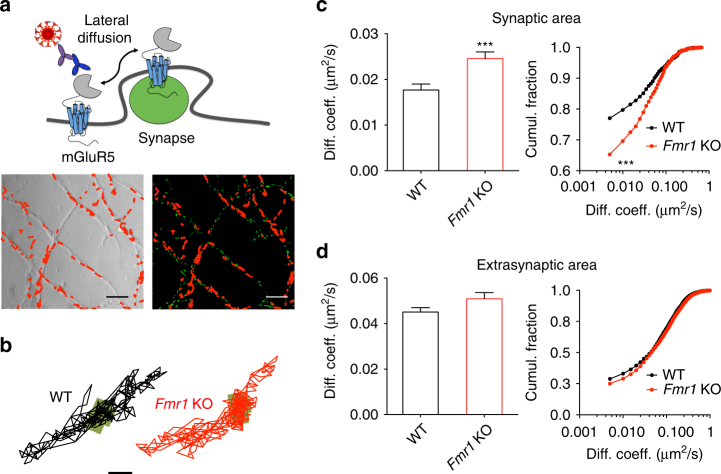
Cell-surface mGluR5 displays an increased lateral diffusion rate within the synaptic compartment of hippocampal *Fmr1* KO neurons. (**a**) Experimental setup. Upper panel: schematic representation of endogenous mGluR5 in the dendritic membrane labeled with a QD-antibody complex targeting the extracellular domain of the receptor. Lower panel: representative images of the dendrites of hippocampal neurons shown in phase contrast (left), and their MitoTracker-labeled synaptic sites (right; green) overlaid with reconstructed trajectories of surface mGluR5-QD complexes (depicted in red) in the dendritic membrane of the same neurons. Scale bar = 5 μm. (**b**) Representative trajectories of single surface mGluR5-QD in WT and *Fmr1* KO neurons. The synaptic sites are represented by the green areas. Scale bar = 1 μm. (**c**) Cumulative distribution (left panel) and cumulative frequency distribution (right panel) of the instantaneous diffusion coefficient of mGluR5-QDs in the synaptic compartment. The lateral diffusion is significantly higher in *Fmr1* KO neurons (WT, 0.017 ± 0.001 µm^2^/s, n = 1632 trajectories from 16 dendritic fields of 3 different cultures); *Fmr1* KO, 0.024 ± 0.001 µm^2^/s, n = 1451 trajectories (14 dendritic fields from 3 cultures); ***P < 0.001 by Mann–Whitney test on cumulative distribution; ***P < 0.001 by Kolmogorov–Smirnov test on cumulative frequency distribution). (**d**) Cumulative distribution (left panel) and cumulative frequency distribution (right panel) of the instantaneous diffusion coefficient of mGluR5-QDs in the extrasynaptic area of WT and *Fmr1* KO neurons (WT, 0.045 ± 0.002 µm^2^/s, *n* = 1907 trajectories from 16 dendritic fields of 3 different cultures; *Fmr1* KO, 0.048 ± 0.002 µm^2^/s, *n* = 1347 trajectories from 14 dendritic fields of 3 different cultures; *P* = 0.106 by Mann–Whitney test on cumulative distribution; *P* = 0.649 by Kolmogorov–Smirnov test on cumulative frequency distribution)

In spite of its prominent role in the pathophysiology of FXS, the dynamics of mGluR5 at synapses have not yet been studied in the context of this disorder. Here we used a single nanoparticle (quantum dot; QD) imaging approach to track surface mGluR5 in live hippocampal neurons derived from second-generation *Fmr1* KO and WT mouse embryos (12–15 days in vitro). This technique permitted us to examine the exploratory activity of single-particle complexes within defined sub-cellular compartments. Synapses were labeled using an active mitochondria marker (MitoTracker) to distinguish them from extrasynaptic sites, as previously described^34^ (Fig. 1a). MitoTracker labeled synaptic sites similarly in WT and KO neurons (Supplementary Fig. 1). An analysis of the trajectories of single mGluR5 molecules revealed that their diffusion coefficient was significantly enhanced within the synaptic compartment of *Fmr1* KO as compared with WT neurons (Fig. 1b, c; 41.18%; *P* < 0.001; WT mobility values were similar to those reported previously^23^). This result indicates an increased mobility of mGluR5 within the synaptic membrane of *Fmr1* KO neurons. Accordingly, the fraction of mobile mGluR5 (diffusion coefficient >0.005 μm^2^/s) at the synapse was higher in *Fmr1* KO neurons (+16.83%; *P* < 0.001). In contrast to the synaptic sites, lateral mobility of mGluR5 at extrasynaptic sites was comparable between *Fmr1* KO and WT neurons (Fig. 1d; diffusion coefficient, *P* = 0.106; mobile fraction, *P* = 0.833).

To determine whether the observed increase in membrane mobility was specific for mGluR5, or a more general phenomenon affecting other glutamate receptors as well, we also quantified the mobility of individual AMPA-type (AMPAR) and NMDA-type (NMDAR) glutamate receptors. To this end, we used antibodies specific to the extracellular domains of the GluA2 and GluN1 receptor subunits comprising the AMPAR and NMDAR tetramer complexes, respectively, in conjunction with the same QD tracking approach. The dynamics of these receptor subunits have been extensively characterized previously using QD-based tracking approaches^34^ (see also Supplementary refs.^1–4^). We found no differences in the lateral diffusion and the mobile fraction of AMPAR within the synaptic compartment (diffusion coefficient, *P* = 0.732; mobile fraction, *P* = 0.913), whereas a small but significant reduction was detected in the extrasynaptic compartment (diffusion coefficient: –4.21%, *P* < 0.001; mobile fraction: –4.53%, *P* < 0.001) of *Fmr1* KO neurons (Supplementary Fig. 2a–c). Conversely, NMDAR showed a small yet statistically significant increase in the lateral diffusion both in the synaptic (diffusion coefficient: +13.33 %, *P* < 0.001) and the extrasynaptic sites (diffusion coefficient: +13.60 %, *P* < 0.001) of *Fmr1* KO neurons, which was not sufficient to affect the fraction of mobile receptors in both compartments (synaptic sites, *P* = 0.517; extrasynaptic sites, *P* = 0.860; Supplementary Fig. 3a–c). These data suggest that the absence of FMRP differentially impacts the mobility of mGluR5, AMPAR and NMDAR, with a major effect for mGluR5 at synaptic sites.

### Impaired mGluR5/Homer scaffold alters mGluR5 diffusion

**Fig. 2:**
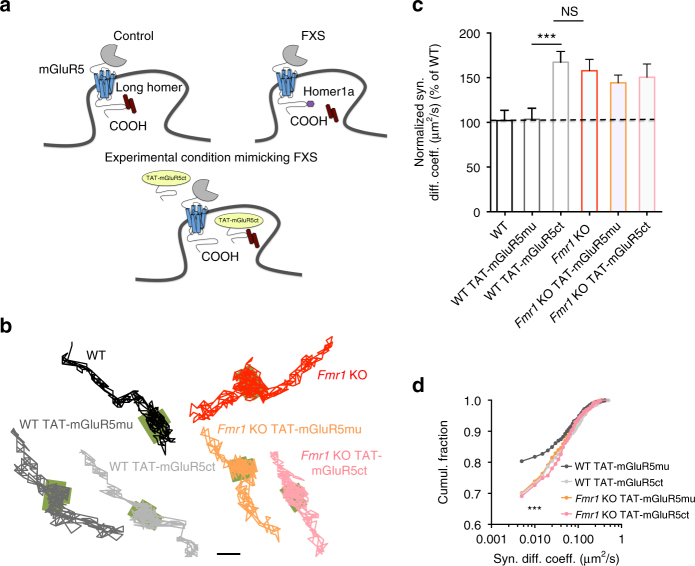
Disruption of the link between mGluR5 and Homer in WT neurons mimics the *Fmr1* KO phenotype. (**a**) Schematic illustration of the effect of the cell-permeable TAT-mGluR5ct peptide. This peptide disrupts the mGluR5/Homer link, mimicking the situation in *Fmr1* KO neurons. (**b**) Representative trajectories of single surface mGluR5-QD in WT and *Fmr1* KO neurons treated with TAT-mGluR5ct and its mutated TAT-mGluR5mu control peptide (both peptides 5 µM, 1 h). The synaptic sites are represented by the green areas. Scale bar = 1 μm. (**c**) Normalized cumulative distribution of the instantaneous diffusion coefficient of mGluR5-QDs in the synaptic area of WT and *Fmr1* KO neurons treated with TAT-mGluR5mu and TAT-mGluR5ct. The lateral diffusion rate of mGluR5-QDs in WT neurons treated with TAT-mGluR5ct peptide is comparable to that in *Fmr1* KO neurons under basal conditions (WT, 0.016 ± 0.002 µm^2^/s, *n* = 636 trajectories from 12 dendritic fields of 3 cultures; WT TAT-mGluR5mu, 0.016 ± 0.002 µm^2^/s, *n* = 1798 trajectories from 12 dendritic fields of 3 cultures; WT TAT-mGluR5ct, 0.026 ± 0.002 µm^2^/s, *n* = 1444 trajectories from 19 dendritic fields of 3 cultures; *Fmr1* KO, 0.025 ± 0.002 µm^2^/s, *n* = 797 trajectories from 16 dendritic fields of 3 cultures; *Fmr1* KO TAT-mGluR5mu, 0.023 ± 0.001 µm^2^/s, *n* = 1419 trajectories from 13 dendritic fields of 3 cultures; *Fmr1* KO TAT-mGluR5ct, 0.024 ± 0.002 µm^2^/s, *n* = 489 trajectories from 9 dendritic fields of 3 cultures; WT TAT-mGluR5mu vs. WT TAT-mGluR5ct ***P < 0.001 by Kruskal–Wallis test with Dunn’s multiple comparison test; WT TAT-mGluR5ct vs. *Fmr1* KO *P* > 0.999 by Kruskal–Wallis test with Dunn’s multiple comparison test). (**d**) Cumulative frequency distribution of the instantaneous diffusion coefficient of mGluR5-QDs within the synaptic area of WT and *Fmr1* KO neurons treated with TAT-mGluR5mu and TAT-mGluR5ct (***P < 0.001 by Kolmogorov–Smirnov test. For statistical analysis each condition was separately compared to WT TAT-mGluR5mu)

Since Homer isoforms function as anchoring molecules for mGluR5 at synapses^25^, we hypothesized that the previously described reduction in mGluR5/Homer interaction^17^ might lead to the exaggerated membrane mobility of mGluR5 in *Fmr1* KO neurons reported here. If our prediction was correct, then disrupting the specific mGluR5/Homer interaction in WT neurons should mimic the disease phenotype. We tested this hypothesis, using a cell-permeable peptide containing the Homer binding motif of mGluR5 (TAT-mGluR5ct; characterized previously^35, 36^; Fig. 2a). Indeed, pre-incubation of WT neurons with TAT-mGluR5ct caused an increase in the lateral diffusion and mobile fraction of mGluR5 in the synaptic compartment (Fig. 2b–d; diffusion coefficient: +62.5%; mobile fraction: +12.57%; WT TAT-mGluR5ct vs. WT TAT-mGluR5mu; *P* < 0.001 for both parameters). Importantly, both parameters were now comparable to those of *Fmr1* KO neurons without the peptide (Fig. 2c, d; diffusion coefficient, *P* > 0.999; mobile fraction, *P* = 0.531). As expected, pre-incubation of WT neurons with a peptide containing a mutated Homer binding motif (TAT-mGluR5mu^35, 36^) had no effect on the lateral diffusion of mGluR5 (Fig. 2c, d; diffusion coefficient, *P* > 0.999; mobile fraction, *P* = 0.795). Moreover, neither TAT-mGluR5ct nor TAT-mGluR5mu treatment had any effect on the mGluR5 mobility in *Fmr1* KO neurons (Fig. 2c, d; diffusion coefficient, *P* = 0.966; mobile fraction, *P* = 0.088; *Fmr1* KO TAT-mGluR5ct vs. *Fmr1* KO TAT-mGluR5mu). Taken together, these experiments provide strong correlative evidence that changes in the lateral diffusion of mGluR5 detected in *Fmr1* KO neurons are indeed due to a disrupted link between the long Homer scaffolding proteins and mGluR5.

### Enhanced mGluR5/NMDAR co-clustering in *Fmr1* KO neurons

**Fig. 3:**
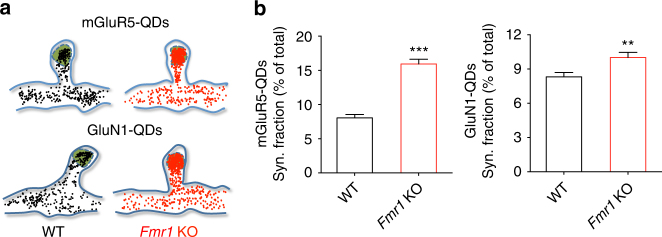
mGluR5 and GluN1 are more confined within the synaptic compartment in *Fmr1* KO neurons. (**a**) Representative surface distribution of mGluR5-QD (upper panel) and GluN1-QD (lower panel) in a 500-frame stack (each dot represents the detection of a single receptor during 50-ms acquisition time), revealing the synaptic site as a trapping zone (green). (**b**) Relative fractions of synaptic mGluR5-QD (left panel) and GluN1-QD (right panel) particles. These values are increased in *Fmr1* KO neurons (mGluR5-QD: WT, 8.053 ± 0.504 %, *n* = 16 dendritic fields from 3 cultures; *Fmr1* KO, 15.95 ± 0.685 %, n = 14 dendritic fields from 3 cultures; ***P < 0.001, *t* = 9.44, df = 28, unpaired Student’s *t*-test; GluN1-QD: WT, 8.318 ± 0.382 %, *n* = 63 dendritic fields from 6 cultures; *Fmr1* KO, 10.02 ± 0.446 %, *n* = 51 dendritic fields from 6 cultures; **P < 0.01, *t* = 2.91, df = 112, unpaired Student’s *t*-test)

In addition to anchoring mGluR5 at synapses, Homer-containing complexes also provide a physical link to NMDARs^29^. We thus explored whether the disrupted mGluR5–Homer scaffold might also alter the interaction between mGluR5 and NMDAR in *Fmr1* KO neurons. In a first set of experiments, we took advantage of the detection accuracy of single QDs (reviewed in ref. ^20^) by measuring the synaptic fraction of mGluR5-QD and GluN1-QD co-localized with MitoTracker (Fig. 3a). We found that the synaptic fraction of both mGluR5-QD and GluN1-QD was increased in *Fmr1* KO neurons (Fig. 3b; mGluR5, *P* < 0.001; GluN1, *P* < 0.01). This finding provides direct evidence that mGluR5 and NMDAR are more confined within the synapse in *Fmr1* KO neurons, likely increasing the probability of physical interactions between these receptors within a given time window.

**Fig. 4:**
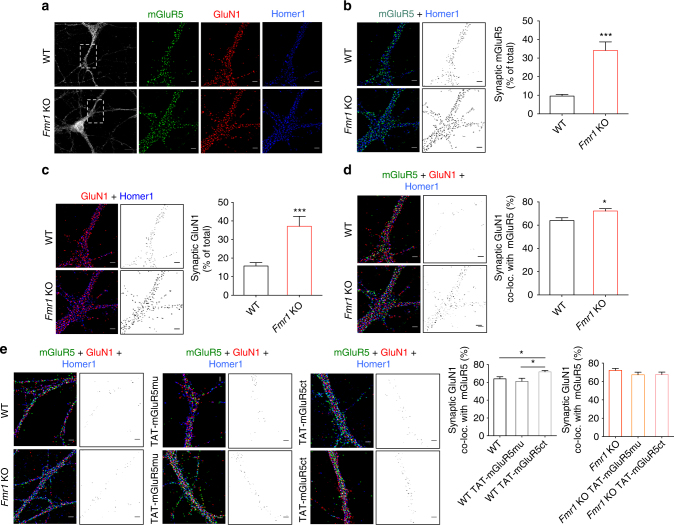
Disruption of the mGluR5/Homer scaffold increases surface mGluR5/NMDAR co-clustering. (**a**) Cultured WT and *Fmr1* KO hippocampal neurons were triple-labeled for mGluR5, GluN1 and Homer1. (**b**, **c**) Left: Representative image of Homer/mGluR5 and Homer/GluN1 co-localization; middle: Distribution of co-localized mGluR5/Homer1 or GluN-/Homer1 clusters; right: mGluR5/Homer1 and GluN1/Homer1 clusters as percentage of total mGluR5 or GluN1 signal (mGluR5: WT, 9.53 ± 0.959 %, *n* = 27; *Fmr1* KO, 34.14 ± 4.598 %, *n* = 20; ***P < 0.001, *t* = 6, df = 45, unpaired Student’s *t*-test; GluN1: WT 15.75 ± 1.841 %, *n* = 29; *Fmr1* KO 37.15 ± 5.324 %, *n* = 18; ***P < 0.001, *t* = 4.47, df = 45, unpaired Student’s *t*-test). (**d**) Left: Representative image showing mGluR5/GluN1/Homer1 colocalization; middle: Distribution of co-localized mGluR5/GluN1/Homer1 labeling; right: Co-localized mGluR5/GluN1/Homer1 clusters as percentage of synaptic GluN1 signal (WT, 63.97 ± 2.414 %, *n* = 26; *Fmr1* KO, 72.14 ± 2.081 %, *n* = 23; *P < 0.05, *t* = 2.53, df = 47, unpaired Student’s *t*-test). (**e**) TAT-mGluR5ct peptide increased mGluR5/GluN1 co-clustering at synapses in WT neurons, whereas TAT-mGluR5mu or TAT-mGluR5ct (both 5 µM, 1 h) had no effect in *Fmr1* KO neurons; Left: Representative images and distribution of mGluR5/GluN1/Homer1-co-labeling signal in control and TAT-mGluR5mu or TAT-mGluR5ct treated WT and *Fmr1* KO neurons. Right: Co-localized mGluR5/GluN1/Homer1-positive signals as percentage of synaptic GluN1 signal (WT: 63.97 ± 2.414 %, *n* = 26; WT TAT-mGluR5mu: 61.19 ± 3.489 %, *n* = 14; WT TAT-mGluR5ct: 71.79 ± 1.528 %, *n* = 22; WT vs. WT TAT-mGluR5ct, *P = 0.043; WT TAT-mGluR5mu vs. WT TAT-mGluR5ct *P *=* 0.017, F (2, 59) = 4.87; *Fmr1* KO: 72.14 ± 2.081 %, *n* = 23; *Fmr1* KO TAT-mGluR5mu: 67.32 ± 2.832 %, *n* = 18; *Fmr1* KO TAT-mGluR5ct: 67.58 ± 2.69 %, *n* = 26; *Fmr1* KO vs. *Fmr1* KO TAT-mGluR5ct *P* = 0.391; *Fmr1* KO TAT-mGluR5mu vs. *Fmr1* KO TAT-mGluR5ct *P* = 0.997, F (2, 64) = 1.13). P values by one-way ANOVA test with Tukey’s Multiple Comparison test. *n* = dendritic fields from 3 cultures. Scale bar = 10 μm (**a**) 2 μm (**b**, **c**, **d**, **e**)

To extend these findings, we performed a triple immunofluorescence labeling experiment for mGluR5, NMDAR and Homer1 (the latter used as a synaptic marker), together with confocal microscopy and post hoc image analysis (Fig. 4a–d). Quantitative analysis of the proportion of mGluR5-/Homer1-positive or GluN1-/Homer1-positive fluorescence intensity (expressed as a function of total mGluR5- or GluN1 signal) suggests an increased localization of both mGluR5 and NMDAR at the synapse in *Fmr1* KO neurons (Fig. 4b, c, respectively; mGluR5, *P* < 0.001; NMDAR, *P* < 0.001). In addition, combined analysis of all three markers points to a higher degree of co-clustering of mGluR5 and NMDAR at synaptic sites (Fig. 4d; *P* < 0.05) further supporting a tighter association of these receptors in the absence of FMRP.

To further examine whether the disrupted mGluR5/Homer scaffold might provide a causal mechanism for this increased co-clustering of mGluR5 and NMDAR in *Fmr1* KO neurons, we again exploited the interfering peptide. As expected, the pre-incubation of WT neurons with TAT-mGluR5ct resulted in a significant increase in the mGluR5/NMDAR co-localization (Fig. 4e; *P* < 0.05), reflecting the increased percentage of synaptic mGluR5 and NMDAR (Supplementary Fig. 4a–c). Pre-incubation with TAT-mGluR5mu had no effect on the co-localization (Fig. 4e; *P* = 0.725; Supplementary Fig. 4a–c). Thus, the disruption of the mGluR5/Homer binding is likely responsible for a tighter physical association between mGluR5 and NMDAR at synapses in *Fmr1* KO neurons.

### Reduced NMDAR function and plasticity in *Fmr1* KO neurons

**Fig. 5:**
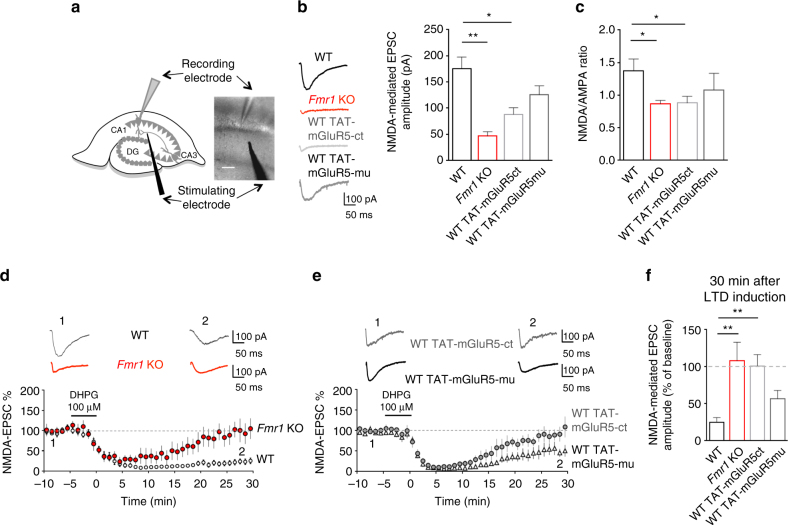
Disruption of mGluR5/Homer coupling alters synaptic NMDAR function and plasticity. (**a**) NMDAR-mediated excitatory post-synaptic currents (EPSCs_NMDA_) were recorded from CA1 pyramidal neurons. Scale bar = 100 μm (**b**) Left: representative EPSCs_NMDA_ traces from WT, *Fmr1* KO, WT treated with either TAT-mGluR5ct or TAT-mGluR5mu (both 5 µM, 4 h). Histograms: EPSCs_NMDA_ amplitude (F_(3, 35)_ = 6.26; **P = 0.0016; one-way ANOVA with Tukey’s Multiple Comparison). Compared to WT (175.7 ± 21.9 pA, *n* = 11 neurons from 7 animals), EPSCs_NMDA_ were lower in *Fmr1* KO (46.4 ± 8.4 pA, *n* = 7 neurons from 4 animals, **P < 0.01) and in WT treated with TAT-mGluR5ct (88.1 ± 12.7 pA, *n* = 13 neurons from 7 animals, *P < 0.05) but not with TAT-mGluR5mu (125.9 ± 16.8 pA n = 8 neurons from 3 animals, *P* = 0.14). (**c**) NMDA/AMPA ratio in WT, *Fmr1* KO, WT treated with TAT-mGluR5ct or TAT-mGluR5mu (F_(3, 32)_ = 4.1; *P = 0.013; one-way ANOVA with Tukey’s Multiple Comparison). NMDA/AMPA ratio (in WT 1.37 ± 0.17, *n* = 9 neurons from 4 animals) was reduced in *Fmr1* KO (0.86 ± 0.05, *n* = 8 neurons from 3 animals, *P = 0.02) and in WT treated with TAT-mGluR5ct (0.88 ± 0.10, *n* = 9 neurons from 3 animals, *P = 0.02). (**d**, **f**) The mGluR1/5 agonist DHPG (100 µM, 5 min) induced long-term depression (mGluR-LTD) of EPSCs_NMDA_ in WT (EPSCs_NMDA_ amplitude: 24.9 ± 2 % of baseline, *n* = 8 neurons from 5 animals) but not in *Fmr1* KO (EPSCs_NMDA_ 107.8 ± 25 %, *n* = 6 neurons from 4 animals, **P < 0.01, WT vs. *Fmr1* KO). (**e**, **f**) mGluR-LTD of EPSCs_NMDA_ was abolished in WT treated with TAT-mGluR5ct (EPSCs_NMDA_ 100.6 ± 15 %, *n* = 8 neurons from 7 animals, **P < 0.01, WT vs. TAT-mGluR5ct) but not with TAT-mGluR5mu (EPSCs_NMDA_ 56.6 ± 11 %, *n* = 7 neurons from 3 animals). (**f**) mGluR-LTD magnitude in all conditions (F_(3, 19)_ = 7.27, **P = 0.0019, one-way ANOVA with Tukey’s Multiple Comparison)

What are the consequences of this tighter mGluR5/NMDAR association at synaptic sites for NMDAR function? To address this question, we measured synaptic NMDAR-mediated excitatory postsynaptic currents (EPSCs_NMDA_) induced by Schaffer collateral stimulation using whole-cell patch-clamp recordings from CA1 pyramidal neurons in acute hippocampal slices (Fig. 5a). EPSCs_NMDA_ displayed lower amplitudes in *Fmr1* KO neurons when compared with WT neurons (Fig. 5b; *Fmr1* KO: 46.4 ± 8.4 pA; WT: 175.7 ± 21.9 pA; *P* < 0.001). Consistently, the NMDA/AMPA ratio was significantly lower in *Fmr1* KO neurons (Fig. 5c; *Fmr1* KO: 0.86 ± 0.05; WT: 1.37 ± 0.29; *P* = 0.02). These defects in NMDAR function were mimicked in WT neurons by application of the interfering peptide TAT-mGluR5ct (Fig. 5b; EPSCs_NMDA_, TAT-mGluR5ct: 88.01 ± 12.70 pA; *P* < 0.01 compared to untreated WT; Fig. 5c; NMDA/AMPA ratio, TAT-mGluR5ct: 0.88 ± 0.10; *P* = 0.02 compared to untreated WT). These data thus strongly support our hypothesis that alterations in the membrane dynamics of mGluR5, and its tighter coupling with NMDAR—in the synapses of both *Fmr1* KO neurons as well as in neurons treated with the disrupting peptide—contribute to abnormal NMDAR function.

It is well established that the activation of group-I mGluR induces an exaggerated LTD of excitatory postsynaptic AMPA currents (EPSC_AMPA_) in hippocampal *Fmr1* KO neurons^13^. In contrast, LTD of excitatory postsynaptic NMDA currents (EPSCs_NMDA_) mediated by group-I mGluR activation^37–39^ has not been investigated in *Fmr1* KO mice. In WT hippocampal CA1 neurons, application of the group-I mGluR orthosteric agonist (*S*)-3,5-dihydroxyphenylglycine (DHPG, 100 µM, 5 min) induced a strong and long-lasting reduction in EPSCs_NMDA_ (Fig. 5d). Remarkably, this form of synaptic NMDAR plasticity was largely absent in *Fmr1* KO neurons (Fig. 5d; *P* = 0.0006). As with the enhanced mGluR5/NMDAR co-clustering, this phenotype was also recapitulated in WT neurons by use of the peptide mimicking approach, suggesting the defective mGluR5/Homer interaction as the underlying cause (Fig. 5e, f; *P* < 0.01). As expected, in *Fmr1* KO slices pre-treated with TAT-mGluR5ct, mGluR-LTD of EPSCs_NMDA_ was still largely absent (*Fmr1* KO TAT-mGluR5ct vs. WT, *P* = 0.032), and comparable to untreated *Fmr1* KO slices (Supplementary Fig. 5a, b; *P* = 0.69**)**. These data suggest that the disruption of the mGluR5/Homer scaffold compromises NMDAR function under both basal conditions, as well as during synaptic plasticity, in *Fmr1* KO neurons.

### Homer1a knockdown rescues NMDAR function and plasticity

**Fig. 6:**
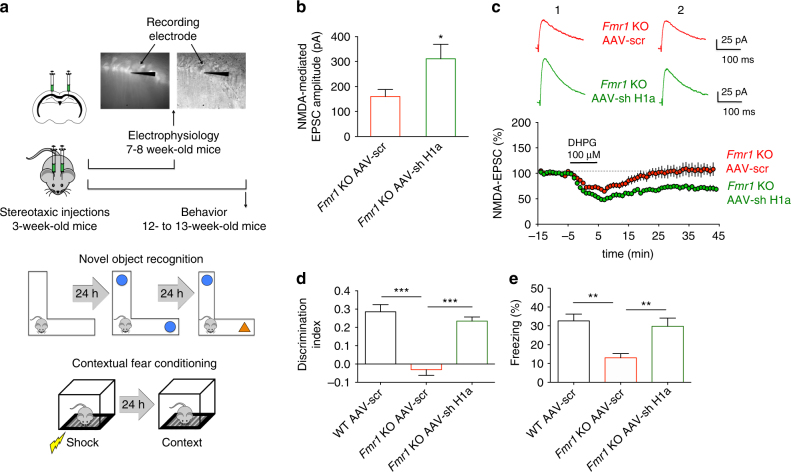
Homer1a knockdown rescues synaptic NMDAR dysfunction and cognitive defects in *Fmr1* KO mice. (**a**) Experimental procedure: electrophysiology (upper panel), novel object-recognition task (NOR) on L-maze (middle panel), contextual fear-conditioning task (CFC) (lower panel). WT and *Fmr1* KO mice (21 days) were bilaterally injected into the dorsal hippocampus with AAV-shH1a or AAV-Scr expressing GFP, and tested at 7–8 weeks (electrophysiology; **b**, **c**) or 12–13 weeks of age (behavior; **d**, **e**). (**b**) The EPSC_NMDA_ amplitude of *Fmr1* KO AAV-sh H1a mice (311.3 ± 58 pA) was larger than that of *Fmr1* KO AAV-scr mice (160.4 ± 27.8 pA, *n* = 12 neurons from 4 animals for both conditions, *P < 0.05, *t* = 2.35, df = 22, unpaired Student’s *t*-test). (**c**) AAV-sh H1a infection in *Fmr1* KO mice rescues LTD of EPSC_NMDA_ induced by DHPG (100 µM, 10 min) (EPSC_NMDA_ amplitude 40 min after LTD induction: 70.4 ± 6 % of control, *n* = 6 neurons from 5 animals). mGluR-LTD of EPSC_NMDA_ was absent in *Fmr1* KO AAV-scr mice (EPSC_NMDA_ amplitude: 106.8 ± 7 % of control, *n* = 7 neurons from 6 animals, ***P < 0.001, *t* = 7.73, df = 117 by unpaired Student’s *t*-test). (**d**) Discrimination index of NOR on test day: WT AAV-scr, 0.285 ± 0.040, *n* = 8; *Fmr1* KO AAV-scr, −0.030 ± 0.032, *n* = 9; *Fmr1* KO AAV-sh H1a, 0.235 ± 0.022, *n* = 8; F (2, 22) = 29.02 (***P < 0.001, one-way ANOVA); *Fmr1* KO AAV-sh H1a mice performed better than *Fmr1* KO AAV-scr, while *Fmr1* KO AAV-scr were impaired cf. WT AAV-scr (***P < 0.001, one-way ANOVA test with Tukey’s test). (**e**) Percentage of time freezing on test day in the CFC (WT AAV-scr, 32.623 ± 3.637 %, *n* = 9; *Fmr1* KO AAV-scr, 13.038 ± 2.203 %, *n* = 8; *Fmr1* KO AAV-sh H1a, 29.743 ± 4.371 %, *n* = 8, F (2, 22) = 8.836, **P < 0.01, one-way ANOVA). *Fmr1* KO AAV-scr exhibited an impaired CFC memory cf. WT AAV-scr, whereas *Fmr1* KO AAV-sh H1a showed rescued CFC memory (**P < 0.01, one-way ANOVA test with Tukey’s test)

Homer1a is known to antagonize the interaction between mGluR5 and Homer. Thus, we asked whether knocking down Homer1a—an approach that fosters the mGluR5/Homer interaction by decreasing the Homer1a/Homer balance^18^—could correct the dysfunction in NMDAR currents in *Fmr1* KO neurons. To address this question we exploited an AAV vector expressing a small interfering hairpin RNA (shRNA) targeted against the unique 3’-untranslated region of Homer1a mRNA and GFP bicistronically^40, 41^. A similar vector expressing scrambled shRNA and GFP served as control^40, 41^. AAV vectors were stereotaxically injected into the hippocampal CA1 area of *Fmr1* KO mice and EPSCs_NMDA_ were measured from infected (i.e., GFP expressing) CA1 pyramidal neurons in acute brain slices 4 weeks later when transgene protein expression had peaked and remained at stable levels (Fig. 6a). EPSCs_NMDA_ displayed higher amplitudes in *Fmr1* KO neurons expressing the shRNA targeted against Homer1a when compared to *Fmr1* KO neurons expressing the scrambled shRNA (Fig. 6b; *P* < 0.05). In addition, the mGluR-dependent LTD of EPSCs_NMDA_, that was absent in *Fmr1* KO neurons, was restored in *Fmr1* KO neurons expressing the shRNA for Homer1a (Fig. 6c; *P* < 0.001). Both findings confirm our hypothesis that mGluR5/Homer disruption can cause abnormal NMDAR function and plasticity in *Fmr1* KO neurons.

### Homer1a knockdown rescues cognitive defects in *Fmr1* KO mice

**Fig. 7:**
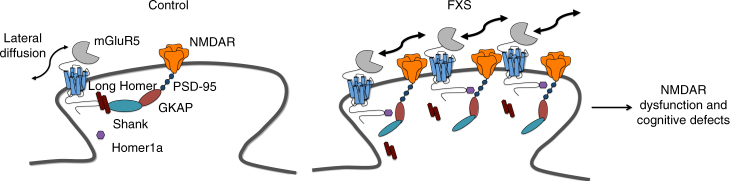
Model for dysfunction of the NMDAR/mGluR5 crosstalk in *Fmr1* KO neurons. In WT neurons, long Homer proteins anchor mGluR5 to a chain of PSD proteins in the synapse and prevent a direct interaction with NMDAR. Under those conditions, a co-clustering of mGluR5 and NMDAR is prevented. In *Fmr1* KO neurons, mGluR5 is less associated with the long Homer proteins and more associated with the short isoform, Homer1a. This disengagement from the long Homer protein–containing complex increases the lateral diffusion of mGluR5 and promotes its interaction with synaptic NMDAR. This configuration at the synapse prevents boosting of NMDAR currents under control conditions and their LTD following mGluR5 stimulation

Can the correction of the disrupted mGluR5/Homer scaffold also rescue cognitive defects linked to NMDAR dysfunction? To evaluate the effects of Homer1a reduction on hippocampus dependent memory formation, we used two behavioral tasks—a one-trial acquisition novel object-recognition task (NOR), performed on an L-maze and which tests episodic memory^42^, and contextual fear conditioning (CFC)^43^. Both procedures induce robust hippocampus dependent learning with a single training episode, and have previously been utilized for the investigation of memory defects in the *Fmr1* ^*tm1Cgr*^ *mouse* ^42, 43^. Since these phenotypes have not previously been investigated in the second-generation *Fmr1* KO mouse model, we initially performed a pilot experiment with a separate batch of experimentally naïve animals to determine whether we could recapitulate these phenotypes. Consistent with the aforementioned studies employing the first-generation *Fmr1* KO mouse line, the second-generation model, used here, exhibited similar decreases in the discrimination index (DI) in the NOR task (Supplementary Fig. 6, Fig. 7, Fig. 8 and Fig. 9; *P* < 0.001) and in the percentage of freezing compared with WT mice following retrieval of CFC memory (Supplementary Fig. 10; *P* < 0.001). We then tested whether mGluR5/Homer crosstalk modulation could correct these defects in another batch of behaviorally naive *Fmr1* KO mice, stereotaxically injected with the aforementioned AAV vectors into the CA1 area of the hippocampus. Importantly, the selective Homer1a knockdown in the hippocampus (Fig. 6a) corrected defects in both the NOR memory (Fig. 6d; *Fmr1* KO AAV-sh H1a vs. *Fmr1* KO AAV-scr, *P* < 0.001; Supplementary Fig. 11, Fig. 12, Fig. 13 and Fig. 14) and CFC memory (Fig. 6e; *Fmr1* KO AAV-sh H1a vs. *Fmr1* KO AAV-scr, *P* < 0.01) in adult *Fmr1* KO mice. Taken together with the aforementioned results, these data strongly support the idea that disruption of mGluR5/Homer scaffold, leading to altered NMDAR function in the hippocampus contributes to impairment of hippocampal-dependent cognitive function in *Fmr1* KO mice.

## Discussion

In spite of the widespread acceptance of the mGluR theory of FXS, the sub-cellular mechanisms underlying mGluR5-dependent defects in synaptic function and plasticity, as well as their associated cognitive phenotypes remain poorly understood. Most studies, to date, have focused on the altered translational processes arising from perturbations in mGluR signaling and their ensuing effect on synaptic plasticity. Surprisingly scant attention, however, has been paid to the behavior of the receptor itself and its interactions with other membrane-bound receptors. Here we present several novel aspects of mGluR5 pathophysiology in FXS. Specifically, we show that not only are the dynamics of mGluR5 altered at the synapse of *Fmr1* KO neurons (leading to a greater lateral diffusion of the receptor), but also that the confinement of the receptor at the synapse is increased. These changes are accompanied by an enhanced co-clustering of mGluR5 and NMDAR at synapses as well as altered NMDAR function/plasticity in the hippocampal CA1 region of *Fmr1* KO mice. Importantly, our experiments point to a plasticity defect not previously reported involving mGluR5-mediated LTD of NMDA receptor currents in FXS. This NMDAR dysfunction and lack of plasticity correlates with hippocampus dependent cognitive deficits.

Previous studies have shown that interactions between mGluR5 and postsynaptic density scaffolding proteins, in particular Homer, are critical for the correct functioning of mGluR5 (reviewed in ref. ^26^). Conversely, disruption of this mGluR5–Homer association in the absence of FMRP^17^ has been reported to play a key causal role in several FXS phenotypes such as anxiety, susceptibility to seizures and changes in circuit level hyperexcitability in the neocortex and hippocampus^18, 19, 44^. To explore whether alterations in mGluR5–Homer crosstalk might contribute to the changes in mGluR5 mobility and NMDAR function reported here, we made use of a peptide disruption approach^35^ to perturb the normal interactions between mGluR5 and long Homer isoforms. Indeed, we found that application of this peptide recapitulated changes in mGluR5 mobility detected in *Fmr1* KO mice. This finding was in coherence with previous studies demonstrating that interaction of mGluR5 with Homer proteins at the postsynaptic site regulates lateral diffusion of mGluR5^45^. The fact that the exaggerated mGluR5 mobility is restricted to the synapse is not surprising taking into consideration that long Homer isoforms are enriched at synaptic sites^28^. Interestingly, these alterations in receptor dynamics appear to be specific to the mGluR5–Homer interaction because the AMPAR subunit, GluA2, which does not bind Homer proteins, did not exhibit substantial alterations in lateral diffusion in the synaptic area. Noteworthy, although these mobility values may be influenced by a number of experimental variables, our measures for WT neurons are consistent with those reported under similar conditions (e.g., ref. ^23^; see also Supplementary Table 1). In addition to alterations in mGluR5 mobility, we also found that application of the peptide led to an increased synaptic confinement of mGluR5, the co-clustering of mGluR5 and NMDAR, and ultimately, to alterations in NMDAR function and NMDAR-dependent plasticity—features that we also observed in *Fmr1* KO neurons. Taken together, these findings suggest a strong causative role for the altered mGluR5–Homer crosstalk in the aforementioned novel changes in mGluR5 dynamics and NMDAR function in FXS (see model in Fig. 7).

Although alterations in synaptic plasticity have been well documented in FXS, this is the first report of a defect in mGluR5-mediated LTD of NMDAR-mediated EPSCs in *Fmr1* KO mice. This is surprising, given that this form of plasticity is well characterized in non-disease models (e.g. refs. ^37–39^). Our results suggest that a disruption of the link between mGluR5 and Homer proteins in *Fmr1* KO neurons plays a negative role in the induction of LTD of synaptic EPSCs_NMDA_ in response to group-I mGluR activation. In this scenario, mGluR5 once liberated from a co-assembly with Homer1 partners would undergo an increased physical and functional interaction with NMDAR, altering the function of this receptor type^31^. In line with previous results, we found that EPSCs_NMDA_ evoked by Schaffer collateral stimulation showed lower amplitudes in *Fmr1* KO neurons. The altered mGluR5/NMDAR partnership might also impact on NMDAR removal from the synapse, a mechanism that is believed to be responsible for depression of synaptic NMDAR currents^38^. In support of this notion, we found that NMDAR is more confined at synapses in the *Fmr1* KO neurons (and in WT neurons following incubation with the TAT-mGluR5ct peptide). One possible mechanism for the inhibition of NMDAR currents might be a direct interaction of NMDAR with G-protein βγ subunits^30^—facilitated by the physical constraint between mGluR5 and NMDA. Similarly, steric hindrance caused by a closer association between NMDAR and mGluR5 could prevent post-translational modification of NMDAR, underlying their removal from synapses. Whereas the induction of group-I mGluR mediated LTD of AMPA currents involves both pre- and post-synaptic mechanisms^46–48^, we suggest that the group-I mGluR mediated LTD of NMDA currents reported here engages a post-synaptic mechanism. This conclusion is based on the post-synaptic location of Homer proteins, and also supported by previous studies into the mechanism of this plasticity form^37^.

How might the absence of FMRP cause mGluR5/Homer disruption and consequently the described changes in mGluR5 and NMDAR function? The increased phosphorylation of Homer has previously been demonstrated to reduce its affinity for mGluR5^49^. On the other hand, the increased phosphorylation of mGluR5 induces a higher affinity for Homer^50^. This would provoke a remodeling of mGluR5–Homer-NMDAR complexes, freeing mGluR5 to form functional associations with NMDAR and potentially leading to the observations reported here. Although this provides a tantalizing explanation for our findings, it may not be the only mechanism underlying these changes. Moreover, given that the loss of FMRP leads to the dysregulated translation of a plethora of target mRNAs it is difficult to pinpoint one kinase as the causative factor. While inhibition of CaMKII in acute slices and cultured neurons from *Fmr1* KO mice has been shown to correct aberrant UP states and frequency of spontaneous firing—features that are indicative of altered circuit level activity^49^—it is conversely possible that altered circuit function can impinge on mGluR5–Homer crosstalk. Altered circuit level activity has been well documented in *Fmr1* KO mice (e.g. refs. ^51, 52^) and may play a role in a number of central phenotypes of the disorder^53^. Increased circuit activity can be expected to influence immediate early gene induction^27, 28^, leading to increased Homer1a expression, which exacerbates crosstalk disruption. This disruption, in turn, may drive further alterations in circuit level function^49^. Interestingly, our experiments involving hippocampal specific knockdown of Homer1a suggest discrete and independent mechanisms for Homer1a in the pathology of FXS. In particular, we show here that hippocampal-dependent defects occur through functional modification of NMDAR. Altered mGluR-dependent NMDAR-LTD might be expected to profoundly impact cognitive functions in FXS. In support of this notion, the plasticity of NMDAR currents has recently been suggested to provide a mechanism for metaplasticity^39, 54^. In addition, hippocampal-dependent cognitive impairment induced by social defeat stress has been linked to a reduced Homer-mGluR5 interaction in mice^55^. Likewise, a selective over-expression of Homer1a in the dorsal hippocampus has been shown to impair spatial working memory^56^.

Here we show the rescue of two hippocampus- and NMDAR-dependent cognitive phenotypes by knockdown of Homer1a expression specifically in the hippocampus. Although previous work has shown that generalized genetic ablation of Homer1a rescued certain behavioral phenotypes of *Fmr1* KO mice related to anxiety and seizure susceptibility^18^, these tests have no direct correspondence with cognitive performance, which is one of the prevailing features of FXS. Here we demonstrate rescue of two phenotypes with direct relevance to cognitive function. Moreover, we demonstrated that knockdown within the hippocampus alone is sufficient to rescue these phenotypes. In addition, rescue of cognitive function was correlated with rescue of a novel synaptic plasticity defect. We are aware that recent literature has raised concerns about the robustness of the cognitive phenotypes reported in the *Fmr1* KO model (reviewed in refs. ^57, 58^). It is thus imperative to establish robust tests that can be replicated between laboratories to strengthen the use of the *Fmr1* KO mouse as a preclinical model of FXS^57^. To this end, the use of the non-classical object–recognition test (performed on an L-maze)^42, 59^, and of a purely contextual fear memory paradigm^43^ rather than auditory fear conditioning (that has typically been used for the *Fmr1* KO model e.g. refs. ^60–63^) may represent an important refinement for cognitive testing of the *Fmr1* KO mouse. Our study, using the second-generation model, replicates findings reported for the first-generation *Fmr1* ^tm1Cgr^ model, suggesting that these tests are robust and reproducible between independent laboratories. With respect to contextual fear memory, the key difference between our work (and the findings of refs. ^43, 64^) and previous findings in the field is that we paired the shock with the presentation of the context. This paradigm leads to a different relevance of the background vs. foreground sensorial cues and the related recruitment of the hippocampus, compared with the protocol used for auditory fear conditioning. This task could thus be more suitable for the detection of behavioral deficits in the *Fmr1* KO mice.

These finding can be expected to have wider implications for future therapeutic approaches for the treatment of FXS and other neurodevelopmental disorders. NMDAR hypofunction has been detected in FXS models in other brain regions and has been recently proposed to contribute to cognitive defects in FXS (reviewed in ref. ^65^). In addition, it has been considered significant in the context of autism and schizophrenia and its correction by means of mGluR5-positive allosteric modulators has been proposed^66^. Indeed, mice with reduced expression of the NMDAR subunit, GluN1 exhibit a range of behavioral phenotypes that are not only consistent with autism spectrum disorder, but also which overlap with those observed in *Fmr1* KO mice. Given the known reciprocal modulation of mGluR5 and NMDA responses, our results of an altered crosstalk of these two receptors in FXS should be taken into consideration when predicting the outcome of single or combined therapy with agents targeting both receptors. Our finding may also contribute to an improved understanding of several factors impeding clinical trial design, such as the marked heterogeneity present within the FXS population^67^. If increased circuit activity (as discussed above) indeed leads to altered Homer1a levels, then this is likely to vary widely amongst individuals based on their responses to external stimuli, environment, etc. Here we propose that therapeutic approaches aimed at restoring the normal mGluR5/Homer and mGluR5/NMDAR interactions might provide a promising alternative for the treatment of FXS. It is hoped that these findings will contribute to the development of alternative, targeted therapies for this disorder and its co-morbidities, and provide mechanistic links to other genetic causes of autism.

## Methods

### Animals

All experiments were conducted in strict compliance with the European Directive (2010/63/EU), and French and Italian law governing the use of laboratory animals and were approved by the Bordeaux Ethics Committee (C2EA50, authorization #5012024-A) and by the Ethics Committee of Catania University (project # 181). Mice were housed in a SPF animal facility prior to experiments, kept on a 12 h–12 h light–dark cycle (lights on at 0:700 am) and had *ad libitum* access to food and water at all times. Second generation *Fmr1* KO mice^32^ were mostly used in our study. This model was generated by deletion of both the promoter and exon 1 of the *Fmr1* gene, as described previously^32^. These mice are distinct from the original *Fmr1* KO (*Fmr1* ^*tm1Cgr*^ *)* mouse line^33^, because they are deficient for both *Fmr1* mRNA and FMRP protein. This mouse model has largely been used for physiological, molecular, and anatomical studies (e.g., ref. ^53^, see also Supplementary refs. ^5–10^), however limited behavioral characterization has also been performed (e.g., ref. ^60^ and Supplementary refs. ^11–14^). Mice were backcrossed six generations into a C57BL/6 J (Charles River, L’Abresle, France) background and maintained in this mixed background for all experiments. The genotype of all progenitors, as well as experimental subjects, was determined by tail PCR as described previously^32^). For dissociated neuronal cultures WT and *Fmr1* KO embryos were generated by crossing homozygous (*Fmr1* ^+/+^ X *Fmr1* ^+/y^ or *Fmr1* ^−/−^ X *Fmr1* ^*−/y*^ *)* progenitor mice. The genotype of the embryos was confirmed by tail PCR of the mother. For electrophysiology and behavioral experiments male WT and *Fmr1* KO littermates were generated by crossing a heterozygous (*Fmr1* ^+/−)^ female mouse with a wild-type (*Fmr1* ^+/y^) male mouse as described previously^53^. Mice were subsequently re-genotyped after the experiment by tail PCR as described previously^53^. Some electrophysiology experiments (see Fig. 5 **)** were carried out on first-generation (*Fmr1* ^*tm1Cgr*^ *) Fmr1* KO mice (on an FVB background)^33^. Genotype was assessed by tail PCR as described previously^68^.

### Primary cell cultures

Cultures of hippocampal neurons and glial cells were prepared from E18 WT and *Fmr1* KO embryos. Pregnant mice were killed by decapitation after deep anesthesia with isoflurane and the uterine horn dissected. Hippocampi were subsequently dissected from the embryos in ice-cold dissection solution and then dissociated in trypsin (Sigma, 0.25%). Briefly, cells were plated at a density of 100 to 200 × 10^3^ cells per milliliter on poly-L-lysine (Sigma) precoated coverslips and kept at 37 °C in 5% CO_2_. The original plating Neurobasal culture medium (Gibco) supplemented with B27 (Gibco, 2%) and complemented with 5% fetal bovine serum was replaced with a serum free medium on day in vitro (DIV) 2. Cytosine B-D-arabinofuranoside (Sigma, 5 μM) was added on DIV 4. All the experiments were performed at DIV 12/15.

### Pharmacological treatments

A cell-permeable (TAT-fused) peptide containing the proline-rich motif (PPXXF) of the mGluR5 C-terminal tail that binds the EVH1 domain of Homer, TAT-mGluR5ct (YGRKKRRQRRR-ALTPPSPFR), and a control peptide with a mutated Homer binding motif, mGluR5mu (YGRKKRRQRRR-ALTPLSPRR), were synthesized at the UT Southwestern Protein Chemistry Technology Center and kindly provided by Prof. K. Huber (Department of Neuroscience, University of Texas Southwestern Medical Center, Dallas, TX 75390, USA; see ref. ^35, 36^). The peptides were dissolved in H_2_0 at a concentration of 5 mM, and aliquots of this stock concentration were stored at −20 °C. Frozen aliquots of both TAT-fused peptides were used within 10 days and diluted to the desired final concentration. Hippocampal cultures were treated with TAT-mGluR5ct or TAT-mGluR5mu for 1 h at a final concentration of 5 µM in serum free culture medium at 37 °C. Slices were incubated during 4 h with either TAT-mGluR5ct or TAT-mGluR5mu (each at 5 µM) in oxygenated ACSF at room temperature (21–22 °C). (S)-3,5-Dihydroxyphenylglycine (DHPG) (Abcam, # ab120007, 100 µM) was dissolved in ACSF and applied by bath perfusion.

### Single-Particle Tracking and Surface Diffusion Calculation

For single-molecule tracking experiments, neurons were first exposed for 10 min to either mouse monoclonal anti-NH_2_ mGluR5 antibody (1:20)^69^, mouse monoclonal anti-GluA2 AMPA receptor (AMPAR) subunit antibody (Millipore, #MAB397, 1:200), or rabbit polyclonal anti-GluN1 NMDA receptor (NMDAR) subunit antibody (Alomone Laboratories, #AGC-001, 1 : 200^34^ and Supplementary refs. ^15–19^) at 37 °C. Neurons were then incubated for 10 min in a solution containing quantum dots (QD) 655 coupled to goat anti-mouse IgG (Invitrogen, #Q11022MP) or coupled to goat anti-rabbit IgG (Invitrogen, #Q-11421MP) (final dilution 1:5000/1 : 10,000) at 37 °C. To label synaptic sites, neurons were incubated for 40 s at RT (~ 22 °C) in a solution containing the orange mitochondria marker MitoTracker (Invitrogen, #M-7510, 20 nM). A fraction of coverslips was also incubated for 1 h with TAT-mGluR5ct or TAT-mGluR5mu (5 µM) in culture medium at 37 °C before and during the incubation with the primary antibodies. For QD 655 fluorescence imaging we used an EM-CCD camera (Evolve 512, Photometrics) with a 512 × 512 imaging array together with an HXP-120 light source (Zeiss) and the appropriate filters for excitation and emission. Images were acquired at an integration time of 50 milliseconds for up to 500 consecutive frames (24 s) as described previously (Supplementary refs. ^15,18^). QD movements were followed on randomly selected healthy looking dendritic regions for up to 20 min, and analyzed using Metamorph software (Universal Imaging Corporation, PA, USA). Briefly, the instantaneous diffusion coefficient, D, was calculated for each trajectory, from linear fits of the first 4 points of the mean-square-displacement vs. time function using the following equation: MSD (t) =  < r2 > (t) = 4Dt. To assign synaptic localization, trajectories were sorted into extrasynaptic (i.e. MitoTracker-negative pixels) and synaptic regions (MitoTracker-positive pixels). To determine the distribution and synaptic fraction of single QD complexes, frame stacks were obtained, and on each frame the receptor/QD complexes were precisely located in synaptic and extrasynaptic compartments. Then, those locations were projected on a single image, providing a high-resolution distribution of the receptor/QD complexes.

### Immunocytochemistry and confocal analysis

The surface expression of mGluR5 was studied using an antibody against the NH_2_ terminal of the mGluR5 in non-permeabilized neurons (see above QD tracking experiments). After removing the medium, cell cultures were incubated with the antibody (1:10) for 30 min at 37 °C. Subsequently, cultures were fixed with a solution containing 4% paraformaldehyde (PFA) and 4% sucrose for 10 min at RT, permeabilized in PBS containing 0.1% Triton-X for 10 min, incubated with blocking solution containing 4% BSA for 45 min at RT, followed by incubation with the rabbit monoclonal anti-GluN1- NH_2_ antibody (Alomone Labs, #AGC-001, 1:200) and the Guinea pig polyclonal anti-Homer1 antibody (Synaptic Systems, #160 004, 1:500) for 1 h at RT. After washing, cultures were incubated for 45 min at RT with the appropriate secondary fluorescent antibodies (Alexa Fluor 647 anti-mouse, Invitrogen, #A-21236, 1:750; Alexa Fluor 555 anti-rabbit, Invitrogen, #A-21428, 1:750; Alexa Fluor 488 anti-Guinea pig, Invitrogen, #A-11073, 1:750). Some coverslips were also incubated for 1 h with TAT-mGluR5ct or TAT-mGluR5mu (final concentration for each: 5 µM in culture medium) at 37 °C before the incubation with anti-mGluR5 antibodies. For immunohistochemistry on fixed brain tissue for the evaluation of transduction efficiency, free-floating 50 µm slices were permeabilized in blocking solution (3% BSA, 10 % normal goat serum in 1× PBS) containing 0.5% Triton-X for 90 min, then incubated overnight with anti-NeuN (Millipore, clone 60, #MAB377). After washing, slices were incubated for 2 h at RT with Alexa Fluor 594-conjugated goat anti-mouse, Invitrogen, #A-11032, 1 : 500) and counter-stained with DAPI.

Images were acquired to measure co-localization of mGluR5, GluN1 and Homer1, using a commercial Leica DMI6000 TCS SP5 confocal microscope with identical settings for all conditions. Ten individual confocal images per coverslip were acquired at 12-bit depth with a pixel size of 96.2 × 96.2 nm (×63 objective, 1.4 NA, 2.5 digital zoom, 1024 × 1024 pixel per image, 50 Hz scanning speed, 98.41 × 98.41 µm field of view). Images were processed with AutoquantX software (MediaCybernetics) and ImageJ software. A minimum of eight randomly chosen cells per condition was acquired and analyzed. A 2D blind deconvolution algorithm was first applied to each image in order to retrieve better data from our images. Then, analysis of the co-localization of mGluR5, GluN1 and Homer1 was performed using the “Co-localization” module of ImageJ (version 1.49; Scion Image, Frederick, MD). A custom-made macro was used to analyze the dendritic part of each image by measuring the fluorescence intensity of each label using fixed threshold intensities.

For the analysis of the ability of the colocalization of MitoTracker and synapses in WT and *Fmr1* KO neurons, cultures (12–15 DIV) were incubated with MitoTracker (Invitrogen, #M-7510, 20 nM) for 40 min at 37 °C and fixed with 3.7% PFA for 10 min. Then, cultures were permeabilized in PBS containing 0.2% Triton-X for 10 min, and incubated with a blocking solution containing 4% BSA and 0.2% Triton-X for 20 min. Next, cultures were incubated for 90 min at RT with the following primary antibodies: Guinea pig polyclonal anti-Homer antibody (Synaptic System, #160 004, 1 : 500) or mouse monoclonal anti-PSD 95 (Thermoscientific # MA1-046, 1:750); rabbit polyclonal anti-Bassoon antibody (Synaptic System #141 003, 1 : 1000); after washing, cultures were incubated for 45 min with the secondary fluorescent antibodies: Biotin-SB-conjugated affinity pure donkey anti-guinea pig (Jackson Immunoresearch, #706-065-148, 1:250) and Cy5 conjugated affinity pure goat anti-rabbit (Jackson Immunoresearch, #111-175-144, 1:500), or Cy5 conjugated affinity pure goat anti-mouse (Jackson Immunoresearch, #115-175-146, 1:500) and Alexa Fluor 488-conjugated affinity pure goat anti-rabbit (Jackson Immunoresearch, #111-545-144, 1:500). Cultures incubated with the anti-Homer antibody were then incubated for 30 min with Fluorescein Streptavidin (Vector #SA-5001, 1:250). Images were acquired using either a LSM-510 Meta confocal microscope (Zeiss) or Leica DMI6000 TCS SP5 and respecting Nyquist sampling parameters (voxel size: 75.2× 75.2× 209 nm; using 63 × 1.4 NA oil DIC, 1.6× digital zoom, 2048 × 2048 pixel size per image, 12 bit depth, 154 × 154 µm field of view and 100 Hz scanning speed). To establish acquisition parameters, a negative control (without primary antibodies) was used and images from WT and *Fmr1* KO were acquired using identical settings. Images were then thresholded and analysis of colocalization of MitoTracker-positive puncta with synapses was performed on randomly selected dendrites using the “Colocalization module” of ImageJ software in two steps. First, the synapses were identified by the colocalization of the post-synaptic markers Homer or PSD-95 with the pre- synaptic marker Bassoon, and then the colocalization between synapses and MitoTracker was calculated.

### Production of recombinant adeno-associated virus

Homer1a mRNA was targeted by RNA interference-mediated silencing. The recombinant adeno-associated viruses (AAVs) were composed of biscistronic expression of short hairpin RNA, driven by the mouse RNA polymerase III U6 promoter (oligonucleotides corresponding to Homer1*a*-specific shRNA (shH1a; sense strand, 5′-GGAGCAUUGAGCUAAUUAUTT-3′; antisense strand, 5′-AUAAUUAGCUCAAUGCUCCTT-3′; Sigma Genosyses), and of control shRNA (sense strand, 5′-GUACUGCUUACGAUACGGTT-3′; antisense strand, 5′-CCGUAUCGUAAGCAGUACUTT-3′), combined with GFP, expressed under the control of the CBA promoter (Supplementary Fig. 15). The entire cassette was flanked by AAV2 inverted terminal repeats (ITRs). Briefly, rAAV2/1 vectors (shCtrl and shH1a) were produced by polyethylenimine (PEI)-mediated triple transfection of HEK 293 cells. HEK 293 cells were cotransfected with the AAV cis plasmid (pAAV-U6-shRNA-CBA-GFP-WPRE-bGH, control shRNA or Homer1a-specific shRNA^40, 41^, the AAV1 (pH21) and AAV2 (pRV1) helper plasmids, and the adenovirus helper plasmid (pFD6). 72h after transfection, cells were collected and lysed by three sequential freeze–thaw cycles (−80/37 °C). The AAV vectors were purified using heparin affinity columns (GE HealthCare), concentrated using Amicon ultra-4 centrifugal filter units with a 100,000 molecular weight cutoff (Millipore) and filtered through a 13 mm diameter 0.2 μm syringe filter. Genomic titers were determined on the basis of an AAV2 ITR sequence-specific qPCR (Supplementary ref. ^20^), using a LightCycler 480 Real-Time PCR System (Roche, Meylan, France).

### In vitro validation of the rAAV-mediated gene silencing

Hippocampal cultures (13 DIV) were infected with AAV vectors (5 × 10^9^ genome copies per 60 mm plate). 5 days after the infection with the shH1a-AAV or shCtrl-AAV, the primary infected neurons were collected and the total RNA extractions were performed using the Trizol reagent (Invitrogen) according to the manufacturer’s instruction. The integrity of the RNA was checked by capillary electrophoresis using the RNA 6000 Nano Lab-on-a-Chip kit and the Bioanalyzer 2100 (Agilent Technologies, Palo Alto, CA, USA). cDNA was synthesized from 2 µg of total RNA with Revert Aid Premium Reverse Transcriptase (Fermentas, St. Leon-Rot, Germany) and random primers (Fermentas).

QPCR was perfomed using a LightCycler 480 Real-Time PCR System (Roche, Meylan, France). QPCR reactions were done in duplicate for each sample, using transcript-specific primers, cDNA and LightCycler 480 SYBR Green I Master (Roche) in a final volume of 10 μl (Melting curve, 95 °C for 5 min and 45 cycles of 95 °C 15 s and 61 °C for 30 s).

Primer sequences for mouse Homer1a are Fwd: 5′-AATTTGAACCCACCGCCTTA-3′ and Rev 5′GGTCATTTCGCTCACGTCTTC-3′. The PCR data were exported and analyzed in an informatics tool (Gene Expression Analysis Software Environment) developed at the NeuroCentre Magendie. For the determination of the reference genes, the Genorm method was used. Relative expression analysis was corrected for PCR efficiency and normalized against two reference genes (mGADPH, Fwd: 5′-TCAAGAAGGTGGTGAAGCAG-3′ and Rev: 5′-TGGGAGTTGCTGTTGAAGTC-3′) and mNono, Fwd 5′-CTGTCTGGTGCATTCCTGAACTAT-3′ and Rev 5′-AGCTCTGAGTTCATTTTCCCATG-3′). The relative level of expression was calculated using the comparative (2^-∆∆CT^) method and controls were arbitrarily set at 1. Transduction of primary hippocampal cultures with shH1a-AAV-resulted in a reduction of *homer1a* mRNA levels by 65–67% relative to those transduced with shCtrl-AAV (Supplementary Fig. 15).

### AAV vector administration

Briefly, WT and *Fmr1* KO mice (post natal age 21 days) were anaesthetized with an isofluorane/air mix (3% for initial induction and 1.5–2% for maintenance), head-fixed in a stereotaxic apparatus (David Kopf Instruments), and placed on a heating pad (HP-1M, Physitemp Instruments, Inc.) connected to a controller (TCAT-2LV, Physitemp Instruments, Inc.) set to maintain the body temperature at 37 °C. Three hundred nanoliters of either AAV-shScr or AAV-shH1a were injected bilaterally into the dorsal hippocampus (Supplementary ref. ^21^; mouse brain atlas; coordinates: −1.95 mm AP, ± 1.25 mm ML, −1.35 mm DV from bregma). The viruses were injected at the rate of 75 nL/min. The injections were performed using a 34-gauge needle (World Precision Instruments) attached to a 10 µL-NanoFil microsyringe (Nanofil, World Precision Instruments). The microsyringe was driven by an electronic micropump system (UltraMicroPump, World Precision Instruments) connected to a microprocessor controller (Micro4, MicroSyringe Pump Controller). Treatments (shCtrl and shH1a) were randomly assigned to individual mice, and balanced within a litter. Electrophysiological experiments began 4 weeks after virus infusion when transgene protein expression had peaked to remain at stable levels (Supplementary ref. ^22^). Behavioral testing started 9 weeks after virus infusion. Injection of AAV vectors resulted in the efficient infection of about 97% of CA1 neurons (Supplementary Fig. 15).

### Electrophysiological recordings of NMDAR currents

Acute hippocampus slices were prepared from male 11–15-day-old WT and *Fmr1* KO mice (for experiments performed at University of Catania; Fig. 5, Supplementary Fig. 5). Brains were removed and placed in oxygenated ice-cold artificial cerebrospinal fluid (ACSF; in mM: NaCl 124; KCl 3.0; NaH_2_PO_4_ 1.2; MgSO_4_ 1.2; CaCl_2_ 2.0; NaHCO_3_ 26; D-glucose 10; pH 7.3), and transversal slices of dorsal hippocampus (300 µm thick) were cut using a vibratome (Leica VT1200). Slices were allowed to recover for at least 3 h at RT. For some experiments slices were incubated with 5–10 µM TAT-mGluR5ct or TAT-mGluR5mu for 4 h at RT.

For electrophysiological recordings, slices were transferred to the recording chamber and visualized using a Leica DMLFS microscope equipped with 20X/0.3 nA and 40×/0.80 nA objectives and differential interference contrast (DIC). A tungsten monopolar electrode (WPI) was placed in the stratum radiatum to stimulate Schaffer collaterals using negative current pulses (duration 0.3 milliseconds), delivered every 30 s by a stimulus generator (A310 Accupulser with A360 stimulus isolator unit, WPI, USA). Stimulation intensity was set to induce 80% of maximal EPSC amplitude. Evoked EPSCs were recorded at RT from CA1 pyramidal neurons in the whole-cell patch-clamp configuration at a holding potential of −60 mV using an EPC7-plus amplifier (HEKA, Germany). Current traces were filtered at 3 kHz and digitized at 10 kHz. The NMDA-to-AMPA ratio (NMDA/AMPA) of evoked EPSCs was measured following standard procedures (Supplementary ref. ^23^). Patch pipettes (open-tip resistance of 1.5–3 MΩ) were filled with intracellular solution containing cesium (130 mM) and tetraethylammonium (TEA, 10 mM). Mixed NMDA/AMPA EPSCs were evoked in ACSF containing bicuculline (5 µM) and glycine (10 µM). EPSCs were evoked every 15 s and recorded at a holding potential of −90 mV, 0 mV, and +50 mV. For NMDA/AMPA ratio measurements, at least twenty traces were averaged at holding potential (HP) of −90 and +50 mV respectively. At a HP −90 mV, EPSCs were exclusively mediated by activation of AMPA receptors (EPSC_AMPA_). The time to peak of EPSC_AMPA_ at HP of −90 mV was used to establish the time window for measuring the peak EPSC_AMPA_ at +50 mV. A complete decay to baseline of EPSC_AMPA_ at HP of −90 mV generally occurred 40 ms after the stimulus artifact. This delay was used as a time window to measure the amplitude of the current mediated by NMDAR (EPSC_NMDA_) at a HP +50 mV. The amplitude of EPSC_NMDA_ at +50 mV divided by the amplitude of EPSC_AMPA_ at +50 mV was taken as the NMDA/AMPA ratio.

For the LTD experiments, patch pipettes were filled with intracellular solution containing (in mM): K-gluconate 140; HEPES 10; NaCl 10; MgCl_2_ 2; EGTA 0.2; QX-314 1; Mg-ATP 3.5; Na-GTP 1; pH 7.3. Slices were continuously perfused with ACSF at a flow rate of 1.0 ml/min. Following whole-cell access, the slice was perfused with Mg^2+^-free ACSF containing CNQX (10 μM), glycine (10 μM) and bicuculline (5 μM) to isolate EPSC_NMDA_. DHPG (100 µM) was dissolved in the same ACSF and bath-applied for 5 min. Data were acquired and analyzed using Signal software (Cambridge Electronic Design, England). EPSC_NMDA_ amplitude values were calculated as the difference between peak current and baseline, averaged every minute, and expressed as percentage of control (calculated from EPSCs recorded during at least 15 min prior to DHPG application). LTD measurements on the same animal model (wild-type and *Fmr1* KO mice on FVB and C57BL6J background) have previously been performed in our laboratory^47, 48^, demonstrating the validity of our DHPG-LTD protocol.

For the electrophysiological recordings of NMDAR currents from *Fmr1* KO mice (second generation) injected with AAV-sh H1a or AAV-scr, hippocampal slices were prepared 4 weeks following viral injection (age of mice: 7–8 weeks) (for experiments performed at IINS-CNRS; Fig. 6). Sagittal slices of dorsal hippocampus (350-μm-thick) were cut in oxygenated ice-cold cutting solution (in mM: Choline-Cl 110; KCl 2.5; NaH_2_PO_4_ 1.25; MgCl_2_ 7; CaCl_2_ 0.5; NaHCO_3_ 25; D-glucose 20; Na-Ascorbate 5; Piruvate Acid 3, pH 7.3), and allowed to recover for at least 30 min at 34 °C in oxygenated `recovery’ solution (in mM: NaCl 110; KCl 2.5; NaH_2_PO_4_ 1.25; MgCl_2_ 7; CaCl_2_ 0.8; NaHCO_3_ 25; D-glucose 20, Na-Ascorbate 1.3; Pyruvate Acid 3, pH 7.3). For electrophysiological recordings, slices were superfused with ACSF at RT containing (in mM): NaCl 125; KCl 2.5; NaH_2_PO_4_ 1.25; MgCl_2_ 1.3; CaCl_2_ 2.3; NaHCO_3_ 25; D-glucose 20, pH 7.3. ACSF also contained NBQX (20 μM), glycine (10 μM) and bicuculline (10 μM) to isolate EPSC_NMDA_. Cells were visualized using a Nikon FN-S2N microscope equipped with 10X/0.30 and 60X/1.0 W objectives and DIC. A glass pipette (open-tip resistance of 3.5–5 MΩ) was placed in the *stratum radiatum* to stimulate Schaffer collaterals with positive current pulses (duration 0.2 milliseconds), delivered every 20 s by a stimulus generator (DS3 constant current isolated stimulator, Digitimer Ltd., England). Evoked EPSC_NMDA_ were recorded at RT from CA1 pyramidal neurons expressing GFP (successfully infected with AAV-sh H1a or AAV-scr) in the whole-cell patch-clamp configuration at a holding potential of +40 mV using an EPC10 amplifier (HEKA, Germany). Current traces were filtered at 3 kHz and digitized at 10 kHz. Patch pipettes (open-tip resistance of 2.5–4 MΩ) were filled with intracellular solution containing (in mM): CsMeSO_3_ 133; HEPES 10; NaCl 4; MgCl_2_ 2; EGTA 0.2; Phosphocreatine 5; Na_2_-ATP 3; Na-GTP 0.4; pH 7.25. For determining the EPSC_NMDA_ Input/Output curve (I/O curve), at least three traces were averaged for all stimulation intensities tested (range: 10–80 µA, 10 µA intervals). For LTD experiments, DHPG (100 µM) was dissolved in the same ACSF and bath-applied for 10 min. Data were acquired using PatchMaster software (HEKA, Germany) and analyzed with Igor software (WaveMetrics, Inc., USA). EPSC_NMDA_ amplitude values were measured as the difference between peak current and baseline, expressed as percentage of control (calculated from EPSCs recorded during at least 10 min prior to DHPG application).

### Behavioral testing

Male mice, obtained from harem breeding composed of two *Fmr1* ^*+/-*^ breeding females and one male (as described above) were maintained in their littermate groups following weaning. No animals were culled from the litters prior to weaning. Male animals were housed in group-cages (42 × 26 × 15 cm; 3–5 individuals per cage). Cages were supplemented with minimal enrichment (cotton nestlets) as required by French law. Every effort was made to ensure that these littermate groups were balanced with respect to genotype (litters containing only one genotype were excluded) and experimental groups were matched with respect to age. Mice were acclimatized to an experimental animal facility for at least 8 weeks prior to behavioral testing (which was started at 12 weeks of age). Behavioral testing was performed in the following order: object-recognition test, open field, contextual fear conditioning. Behavioral phenotypes were initially validated in the second-generation model using naïve animals (*Fmr1* KO and their WT littermates) of equivalent age, which had not undergone surgery to introduce the AAV constructs required for knockdown experiments. All behavior experiments were performed by a female member of the research team, during the light phase of the cycle (between 08:30 and 18:00 hours) and, with the exception of the open field, analyzed blind to genotype. No statistical methods were used to pre-determine sample sizes, but our sample sizes are similar to those used in previous studies^42, 43, 53^.

One-trial-acquisition novel object-recognition (NOR) episodic memory was assayed as described previously^42^ (see Fig. 6a **)** for a schematic representation). Our testing conditions represent a modification of the more ‘classical’ presentation of the test in that mice were tested on an L-maze rather than an open field or circular arena to reduce anxiety and to encourage exploration^70^. The L-maze was made out of gray non-reflective plastic and composed of two corridors (30 cm long × 4.5 cm wide, and 15 cm high walls) set at a 90° angle. Testing was performed under moderate illumination (40–50 lux, measured at the exterior of the maze, 5–10 lux measured at the base of the maze) and each session was filmed (at a rate of 25 frames per second) for post hoc analysis. On day 1, mice were habituated for 10 min to the maze in which the task was performed. On the second day, mice were put back in the maze for 10 min, two identical objects were presented and the time that the mice spent exploring each object was recorded. The choice of object was randomized, but balanced with respect to genotype. On the third day (24 h later) subjects were again placed in the maze for 10 min, one of the familiar objects was replaced with a novel object (the position of the novel object was randomized with respect to the arm of the maze to control for any spatial component). Objects presented were as follows: (1) glass screw cap bottle (6.8 cm tall, approximate diameter 1.9 cm) composed of amber glass and plastic screw cap lid, and (2) plastic Lego piece (3.3 × 3.3 × 2.5 cm). The grooved face of the Lego was presented to the open side of the corridor. All objects were validated in prior tests using a separate group of WT animals of the same genetic background (Supplementary Fig. 16 **)**. The maze and objects were cleaned between each subject with 20% ethanol. Object exploration was analyzed manually by an experienced observer and was defined as the orientation of the nose to the object at a distance of less than 2 cm. In each test session, a discrimination index (DI) was calculated as the difference between the time spent exploring the novel (TN) and the familiar object (TF) divided by the total exploration time (TN+TF): DI = [TN−TF]/[TN+TF]. A higher discrimination index is considered to reflect greater memory retention for the familiar object. Analysis was performed blind to genotype and treatment. All the groups were age-matched and counterbalanced for treatment.

Exploration in an open field was performed in a separate experimental room, using a 38.5 × 38.5 cm arena with 40 cm walls of dark blue Plexiglas. The floor of the arena was white. The room illumination (measured at the center of the arena) was ~ 40 lux. Mice were adapted to a fore-room of the experimental space for at least 30 min prior to the session, although exploration took place in a rear zone, separated by a door. No visual cues were presented. Each mouse explored the arena for 30 min and the session was filmed (25 frames per second) and concomitantly tracked using Ethovision XT acquisition and analysis software (Noldus). The experimenter was not blinded to the genotype or experimental treatment, but all analysis was performed in an automated manner by the software, using predetermined parameters. Distance moved, and velocity, were determined using center point tracking measures.

Contextual fear conditioning (CFC) was assayed as previously described^42^ (Fig. 6a) for a schematic representation). The task took place in a separate room, to which the mouse has not previously been exposed, in a Plexiglas chamber (with a 17 × 25 floor and 22 cm walls (internal dimensions) equipped with overhead infrared lighting. The chamber was composed of three transparent walls, and one opaque wall, giving access to the visuo-spatial cues in the experimental room. The illumination in the room was dim (5–10 lux measured within the chamber and approximately 20 lux measured at the exterior of the chamber). The floor of the conditioning chamber consisted of stainless-steel rods (2 mm diameter), spaced 5 mm apart and connected to a shock generator (Imetronic, Talence, France). The four sides of the chamber and the rods of the floor were cleaned with 70% ethanol before each trial. During conditioning, the mice explored the chamber for 2 min and then received five 2-second-long unsignalled electric shocks (0.4 mA intensity), at 1-minute intervals. The mouse was immediately returned to its home cage at the end of the conditioning session. During the test phase (24 h after conditioning) mice were put back in the same chamber for 6 min, without receiving any shock. Both sessions were filmed (under infrared illumination) and freezing time was scored, post hoc, by an experienced observer. Freezing was defined as the absence of any body movement beside respiration and was normalized by the duration of the test session length to obtain a percent measure. In a subset of experiments, freezing, as well as exploratory behavior (walking, rearing) on the first day was also scored. Experiments and analysis were performed blind to genotype and treatment. All the groups were age-matched and counterbalanced for treatment.

### Data representation and statistical analysis

Group values are presented as mean ± s.e.m. The data normality was tested using the D’Agostino-Pearson omnibus normality test. Comparisons between groups for cumulative distribution of instantaneous diffusion coefficients and for co-localization data from immunocytochemistry experiments were performed using Mann-Whitney test (pair comparison) or Kruskal–Wallis followed by Dunn’s Multiple Comparison test (group comparison). Comparisons of cumulative frequency distribution of instantaneous diffusion coefficients were performed using Kolmogorov-Smirnov test. The comparison of the percentage of immobile and mobile receptors was performed using Chi-square test with Yates’ correction. All of the other comparisons between groups were performed using parametric statistical tests, Student’s t-test (pair comparison), one-way ANOVA (group comparison) followed by Tukey’s Multiple Comparison test when appropriate. Significance levels were defined as *P < 0.05, **P < 0.01, and ***P < 0.001.

### Data availability

The authors declare that all data supporting the findings of this study are available within this article, its Supplementary Information files, or are available from the corresponding authors upon reasonable request

## Electronic supplementary material

Supplementary Information

Peer Review File
