# Supplementary material for: Altered surface mGluR5 dynamics provoke synaptic NMDAR dysfunction and cognitive defects in Fmr1 knockout mice
Source: Nat Commun. 2017 Oct 24;8:1103. doi: 10.1038/s41467-017-01191-2 (PMC5653653; doi:10.1038/s41467-017-01191-2)
Supplement: Supplementary file 1 — Supplementary Information [file 41467_2017_1191_MOESM1_ESM.pdf]

Supplementary Figure 1.

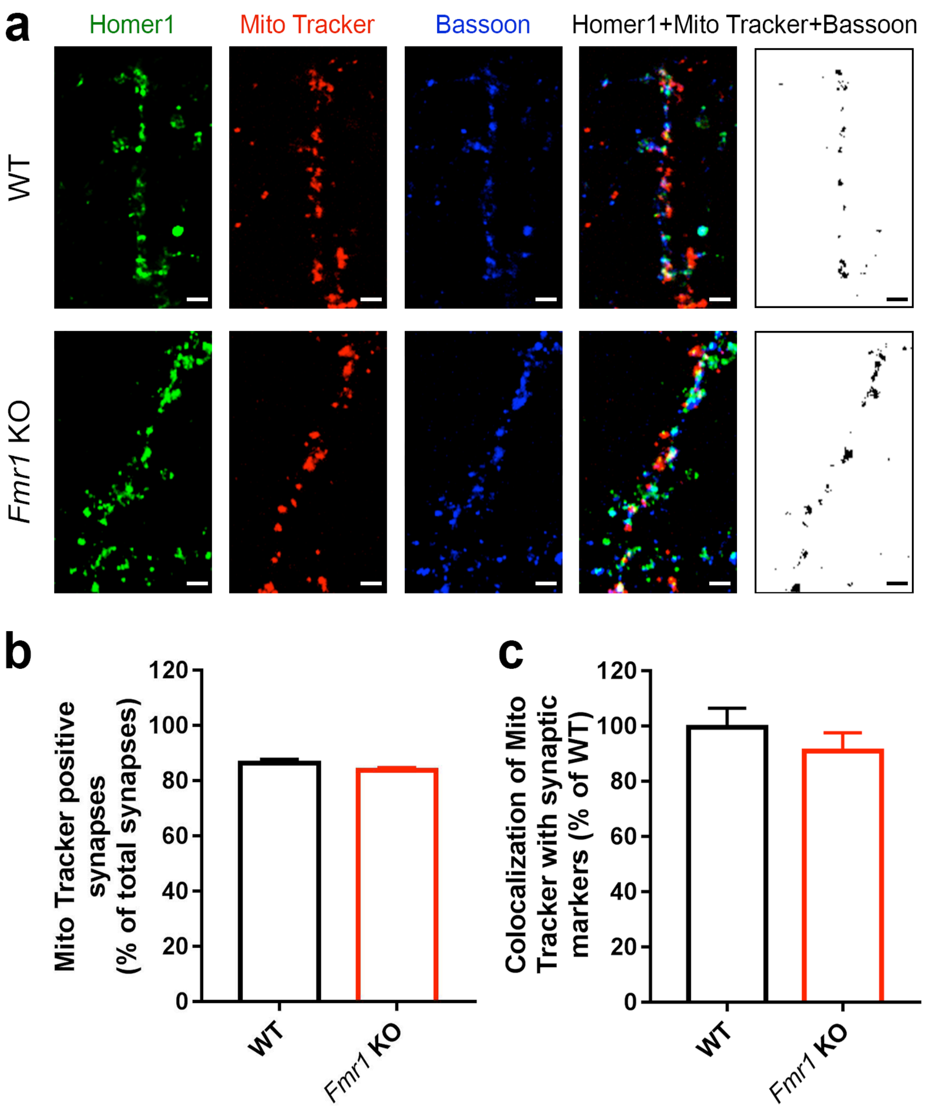

Supplementary Figure 1. MitoTracker equally labels synapses in WT and *Fmr1* KO neurons. (**a**) Cultured hippocampal neurons from WT and *Fmr1* KO mice were triple-labeled with primary antibodies against Homer1 (postsynaptic marker) and Bassoon (presynaptic active zone protein), and with MitoTracker. Representative confocal images of single and triple labeling (left and centre), and co-localization of the three markers obtained with the colocalization module of the program Image J (right panels). Graphs show (**b**) the percentage of synapses labeled with MitoTracker (P = 0.1333 by Mann-Whitney test) and (**c**) the colocalization of Mitotracker at synapses (co-localized Homer/Bassoon) in WT and *Fmr1* KO neurons, expressed as percentage of WT (P = 0.3925 by Mann-Whitney test, n = 53 WT, n = 53 KO, n = total number of dendrites analyzed from two dishes/condition). Identical results were obtained when PSD-95 was used as post-synaptic marker instead of Homer 1. Data are presented as mean ± sem. Scale bar = 2 µm.

Supplementary Figure 2.

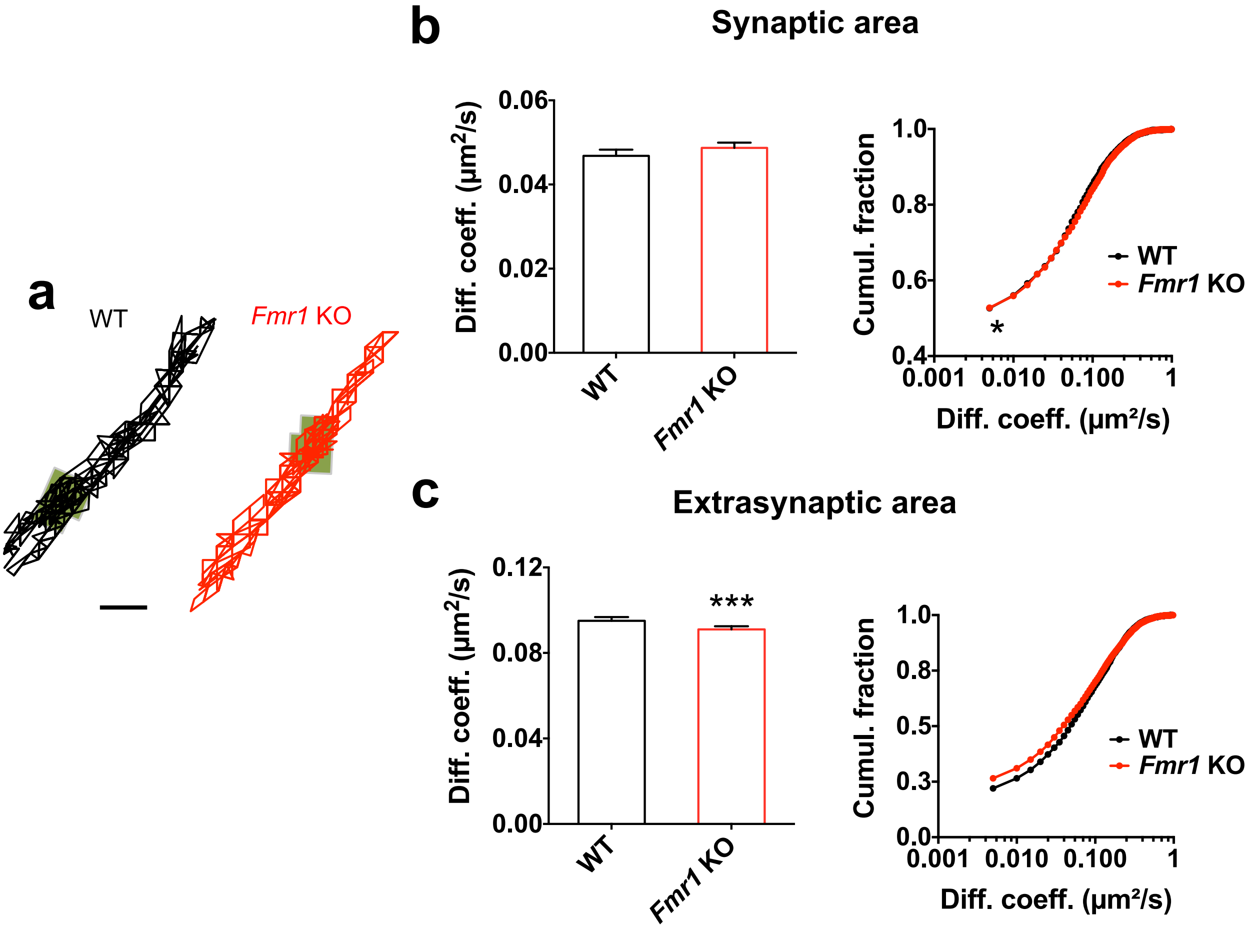

Supplementary Figure 2. The lateral diffusion of the endogenous GluA2-containing AMPA receptor is not changed in the synaptic compartment, and minimally reduced in the extrasynaptic compartment of hippocampal *Fmr1* KO neurons. (**a**) Representative trajectories of single surface GluA2-QD in WT and *Fmr1* KO neurons (500 frames, 20-Hz acquisition, 24-s duration). The synaptic sites are represented by the green areas. Scale bar = 1 µm. (**b**) Cumulative distribution (***left panel***) and cumulative frequency distribution (***right panel***) of the instantaneous diffusion coefficient of GluA2-QDs in the synaptic compartment of WT and *Fmr1* KO neurons. The lateral diffusion of GluA2-QDs is not altered in *Fmr1* KO neurons; WT, 0.047 ± 0.001 µm^2/s, n = 3921 trajectories (33 dendritic fields from 4 cultures); *Fmr1* KO, 0.048 ± 0.001 µm^2/s, n = 6004 trajectories (37 dendritic fields from 5 cultures); P = 0.732 by Mann-Whitney test on cumulative distribution; *P < 0.05 by Kolmogorov-Smirnov test on cumulative frequency distribution). (**c**) Cumulative distribution (***left panel***) and cumulative frequency distribution (***right panel***) of the instantaneous diffusion coefficient of GluA2-QDs in the extrasynaptic area of WT and *Fmr1* KO neurons. The lateral diffusion of GluA2-QDs is slightly decreased in the extrasynaptic compartment of *Fmr1* KO neurons; WT, 0.095 ± 0.002 µm^2/s, n = 4651 trajectories (n = 33 dendritic fields from n = 4 cultures); *Fmr1* KO, 0.091 ± 0.001 µm^2/s, n = 7234 trajectories (n = 37 dendritic fields from n = 5 cultures); ***P < 0.001 by Mann-Whitney test on

cumulative distribution; *P* = 0.534 by Kolmogorov-Smirnov test on cumulative frequency distribution). Data are shown as mean ± s.e.m.

Supplementary Figure 3.

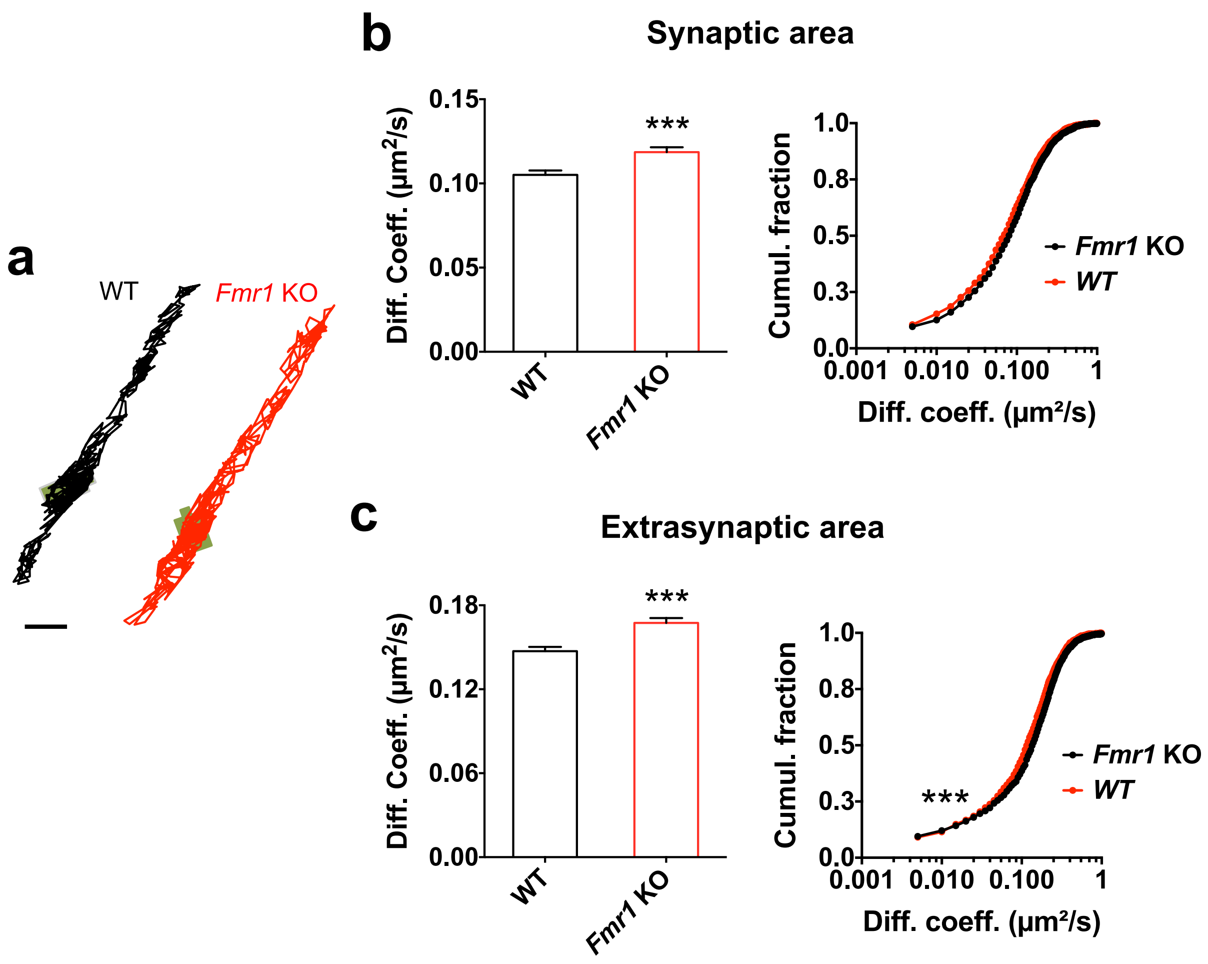

Supplementary Figure 3. The lateral diffusion of the endogenous GluN1-containing NMDA receptor is slightly increased both in the synaptic and extrasynaptic compartments of hippocampal *Fmr1* KO neurons. (**a**) Representative trajectories of single surface GluN1-QD in WT and *Fmr1* KO neurons (500 frames, 20-Hz acquisition, 24-s duration). The synaptic sites are represented by the green areas. Scale bar = 1 µm. (**b**) Cumulative distribution (***left panel***) and cumulative frequency distribution (***right panel***) of the instantaneous diffusion coefficient of GluN1-QDs in the synaptic compartment of WT and *Fmr1* KO neurons. The lateral diffusion of GluN1-QDs is slightly increased in *Fmr1* KO neurons; WT, 0.105 ± 0.003 $\mu m^2/s$, n = 2000 trajectories (63 dendritic fields from 6 cultures); *Fmr1* KO, 0.119 ± 0.003 $\mu m^2/s$, n = 2000 trajectories (60 dendritic fields from 6 cultures); ****P* < 0.001 by Mann-Whitney test on cumulative distribution; *P* = 0.670 by Kolmogorov-Smirnov test on cumulative frequency distribution). (**c**) Cumulative distribution (***left panel***) and cumulative frequency distribution (***right panel***) of the instantaneous diffusion coefficient of GluN1-QDs in the extrasynaptic area of WT and *Fmr1* KO neurons. The lateral diffusion of GluN1-QDs is slightly altered in *Fmr1* KO neurons (WT, 0.147 ± 0.003 $\mu m^2/s$, n = 2000 trajectories (63 dendritic fields from 6 cultures); *Fmr1* KO, 0.167 ± 0.003 $\mu m^2/s$, n = 2000 trajectories (60 dendritic fields

from 6 cultures); ***$P < 0.001$ by Mann-Whitney test on cumulative distribution; ***$P < 0.001$ by Kolmogorov-Smirnov test on cumulative frequency distribution). Data are shown as mean ± s.e.m.

Supplementary Figure 4.

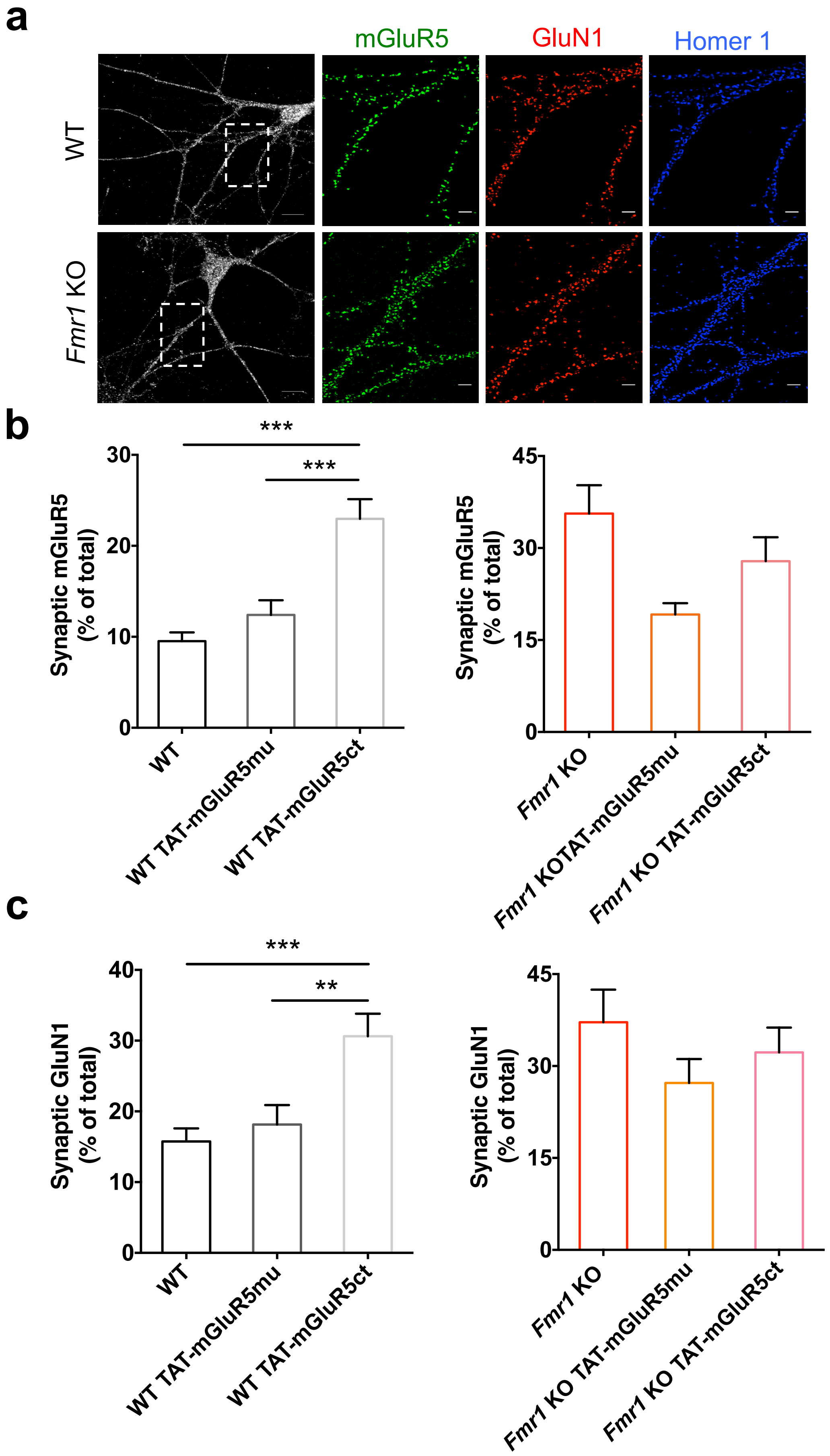

Supplementary Figure 4. Disruption of the link between mGluR5 and Homer proteins increases the synaptic localization of mGluR5 and GluN1 in WT neurons. (**a**) Representative images of cultured hippocampal neurons from WT and *Fmr1* KO mice that were triple-labeled with the primary antibodies anti-mGluR5-NH_2, anti-GluN1-NMDAR-NH_2 and anti-Homer1. (**b,c**) Pretreatment with TAT-mGluR5ct peptide (5 µM, 1h) leads to a significant increase in mGluR5 and GluN1 localization in the synaptic area of WT neurons (***left panels***). Quantitative analysis of the proportion of mGluR5-/Homer1-positive or GluN1-/Homer1-positive fluorescence signals expressed as a function of total mGluR5 or GluN1 signal, respectively (mGluR5, WT, 9.53 ± 0.959 %, n = 27 dendritic fields from 3 cultures; WT TAT-mGluR5mu, 12.41 ± 1.604 %, n = 20 dendritic fields from 3 cultures; WT TAT-mGluR5ct, 22.97 ± 2.161 %, n = 23 dendritic fields from 3 cultures; WT versus WT TAT-mGluR5ct ***$P < 0.001$ by one-way ANOVA test with Tukey's Multiple Comparison test, WT TAT-mGluR5mu versus WT TAT-mGluR5ct ***$P < 0.001$ by one-way ANOVA test with Tukey's Multiple Comparison test, $F_{(2, 67)} = 20.18$; GluN1, WT, 15.75 ± 1.841 %, n = 29 dendritic fields from 3 cultures; WT TAT-mGluR5mu, 18.15 ± 2.748 %, n = 18 dendritic fields from 3 cultures; WT TAT-mGluR5ct, 30.61 ± 3.202 %, n = 27 dendritic fields from 3 cultures; WT versus WT TAT-mGluR5ct ***$P < 0.001$ by one-way ANOVA test with Tukey's Multiple Comparison test, WT TAT-mGluR5mu versus WT TAT-mGluR5ct **$P < 0.01$ by one-way ANOVA test with Tukey's Multiple Comparison test, $F_{(2, 71)} = 9.84$). No differences were observed for mGluR5 and GluN1 localization in *Fmr1* KO neurons treated with either TAT-mGluR5mu or TAT-mGluR5ct (both 5 µM, 1h) (***right panels***) (mGluR5, *Fmr1* KO, 34.14 ± 4.598 %, n = 20 dendritic fields from 3 cultures; *Fmr1* KO TAT-mGluR5mu, 19.17 ± 1.838 %, n= 9 dendritic fields from 3 cultures; *Fmr1* KO TAT-mGluR5ct, 27.86 ± 3.905 %, n= 22 dendritic fields from 3 cultures; *Fmr1* KO versus *Fmr1* KO TAT-mGluR5ct $P = 0.917$ by Kruskal-Wallis test with Dunn's multiple comparison test, *Fmr1* KO TAT-mGluR5mu versus *Fmr1* KO TAT-mGluR5ct $P > 0.999$ by Kruskal-Wallis test with Dunn's multiple comparison test; GluN1, *Fmr1* KO, 37.15 ± 5.324 %, n = 18 dendritic fields from 3 cultures; *Fmr1* KO TAT-mGluR5mu, 27.24 ± 3.9 %, n = 12 dendritic fields from 3 cultures; *Fmr1* KO TAT-mGluR5ct, 32.22 ± 4.061 %, n = 27 dendritic fields from 3 cultures; *Fmr1* KO versus *Fmr1* KO TAT-mGluR5ct $P = 0.396$ by one-way ANOVA test with Tukey's Multiple Comparison test, *Fmr1* KO TAT-mGluR5mu versus *Fmr1* KO TAT-mGluR5ct $P = 0.761$ by one-way ANOVA test with Tukey's Multiple Comparison test, $F_{(2, 54)} = 0.87$). Scale bar = 2 µm (except panel A for low magnification scale bar = 10 µm). All data are shown as mean ± s.e.m.

Supplementary Figure 5.

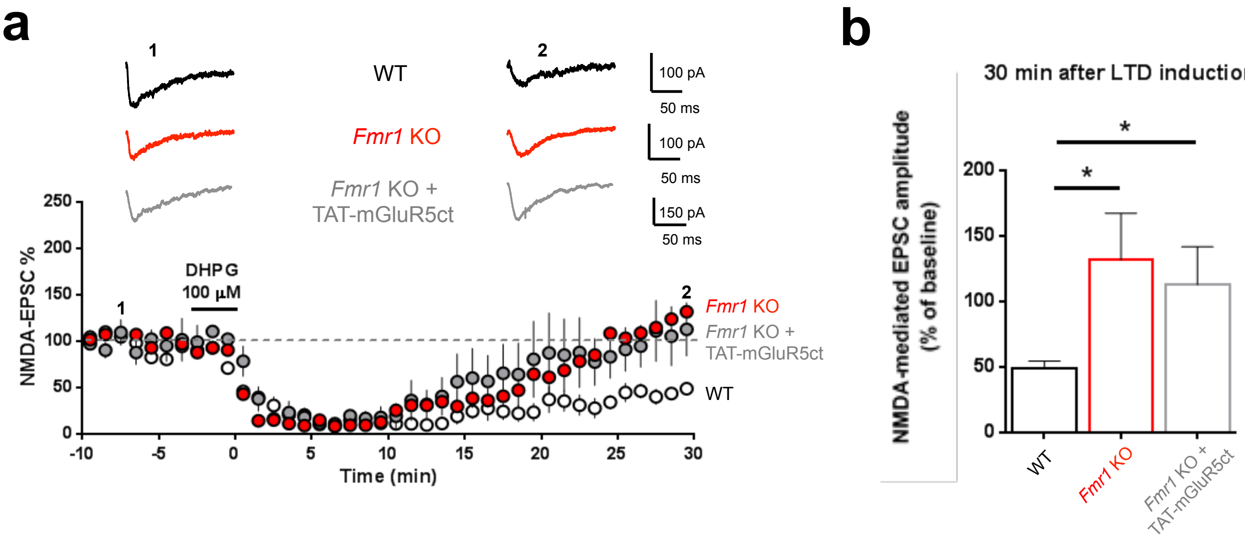

Supplementary Figure 5. Synaptic NMDAR function and plasticity are similarly altered in *Fmr1* KO neurons either untreated or pretreated with TAT-mGluR5ct. (a, b) NMDA receptor-mediated excitatory post-synaptic currents ($EPSCs_{NMDA}$) were recorded under whole-cell patch clamp from CA1 pyramidal neurons in hippocampal slices following stimulation of *Schaffer* collaterals. Representative traces show $EPSCs_{NMDA}$ recorded from CA1 neurons in slices from WT, *Fmr1* KO, *Fmr1* KO pretreated with TAT-mGluR5ct (5 µM, 4 hours). Bath application of the group-I mGluR agonist DHPG (100 µM, 5 min) induced a long-term depression (mGluR-LTD) of $EPSCs_{NMDA}$ recorded in WT slices ($EPSCs_{NMDA}$ amplitude 30 min after LTD induction: 48.9 ± 5.6 % of baseline, n = 6 neurons from 3 animals). mGluR-LTD of $EPSCs_{NMDA}$ was not observed in *Fmr1* KO neurons either untreated ($EPSCs_{NMDA}$ amplitude: 131.8 ± 35.5, n = 5 neurons from n = 4 animals, a value significantly different from WT, **P* = 0.032, t = 2.532, df = 9 by unpaired Student's *t*-test) or pretreated with TAT-mGluR5ct peptide ($EPSCs_{NMDA}$ amplitude: 112.9 ± 28.9, n = 5 neurons from 3 animals, a value significantly different from WT, **P* = 0.04, t = 2.385, df = 9 by unpaired Student's *t*-test). No significant difference was detected between untreated *Fmr1* KO slices and *Fmr1* KO slices pretreated with TAT-mGluR5ct (*P* = 0.69, t = 0.4125, df = 8 by unpaired Student's *t*-test).

Supplementary Figure 6.

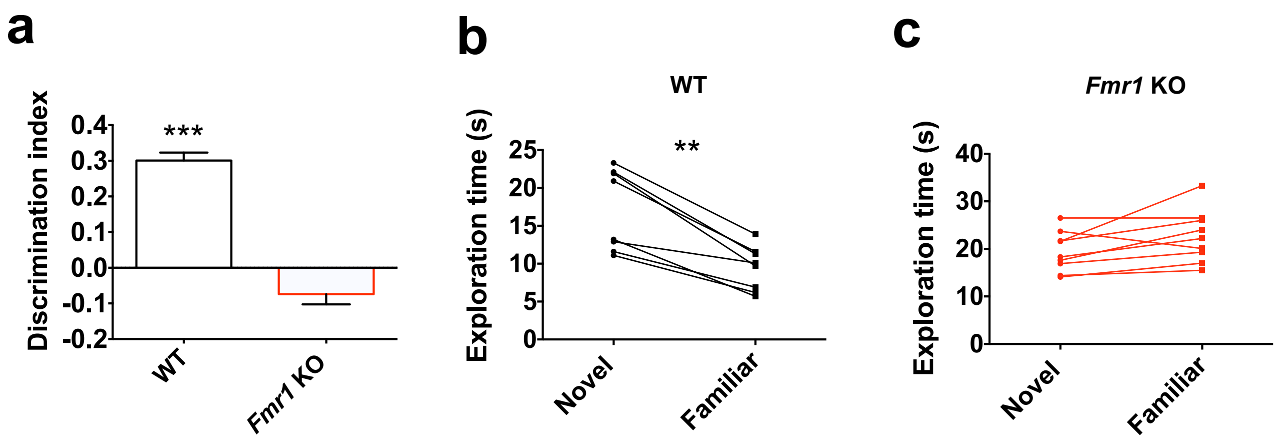

Supplementary Figure 6. Discrimination index (DI) and exploration time for WT and *Fmr1* KO mice in the novel object recognition task. (a) *Fmr1* KO mice show a lower DI when compared with WT mice (WT, 0.301 ± 0.0224, n = 8; *Fmr1* KO, 0.074 ± 0.028, n = 9; ****P* < 0.001, t = 10.2, df = 15 by unpaired Student's t-test). **(b)** WT exploration time (novel, 17.13 ± 1.890 s, n = 8; familiar, 9.425 ± 1.030 s, n = 8; ***P* < 0.01, t = 3.58, df = 10.82 by unpaired Student's t-test). **(c)** *Fmr1* KO exploration time (novel, 19.42 ± 1.408 s, n = 9; familiar, 22.66 ± 1.834 s, n = 9; *P* = 0.181, t = 1.40, df = 16 by unpaired Student's t-test).

Supplementary Figure 7.

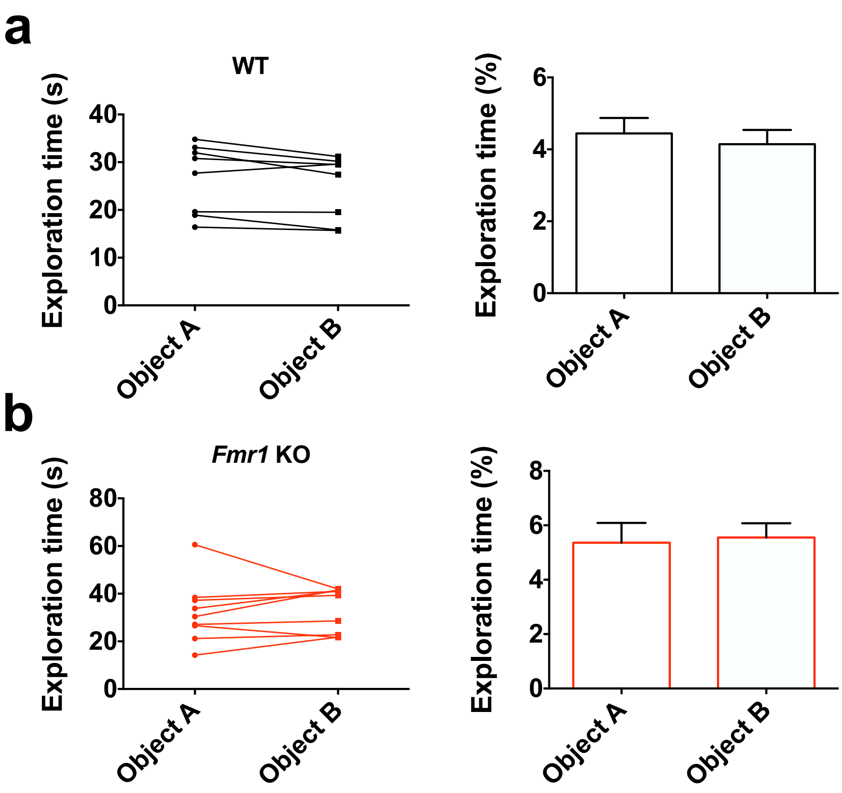

Supplementary Figure 7. Exploration time during the training session (2 identical objects) of the novel object recognition task is identical for WT and *Fmr1* KO mice. (a) Exploration time (***left panel***) and percentage of exploration time (***right panel***) spent by WT mice exploring two identical objects during the training session (day 2 of the protocol) (exploration time, object A, 26.66 ± 2.57 s, n = 8; object B, 24.86 ± 2.37 s, n = 8; *P* = 0.614, t = 0.515, df = 14 by unpaired Student's t-test; percentage of exploration time, object A, 4.44 ± 0.43 %, n = 8; object B, 4.14 ± 0.39 %, n = 8; *P* = 0.614, t = 0.515, df = 14 by unpaired Student's t-test). **(b)** Exploration time (***left panel***) and percentage of exploration time (***right panel***) spent by *Fmr1* KO mice exploring two identical objects during the training session (day 2 of the protocol) (exploration time, object A, 32.17 ± 4.37 s, n = 9; object B, 33.32 ± 5.38 s, n = 9; *P* = 0.833, t = 0.215, df = 16 by unpaired Student's t-test; percentage of exploration time, object A, 5.36 ± 0.73 %, n = 9; object B, 5.55 ± 0.52, n = 9; *P* = 0.833 %, t = 0.215, df = 16 by unpaired Student's t-test).

Supplementary Figure 8.

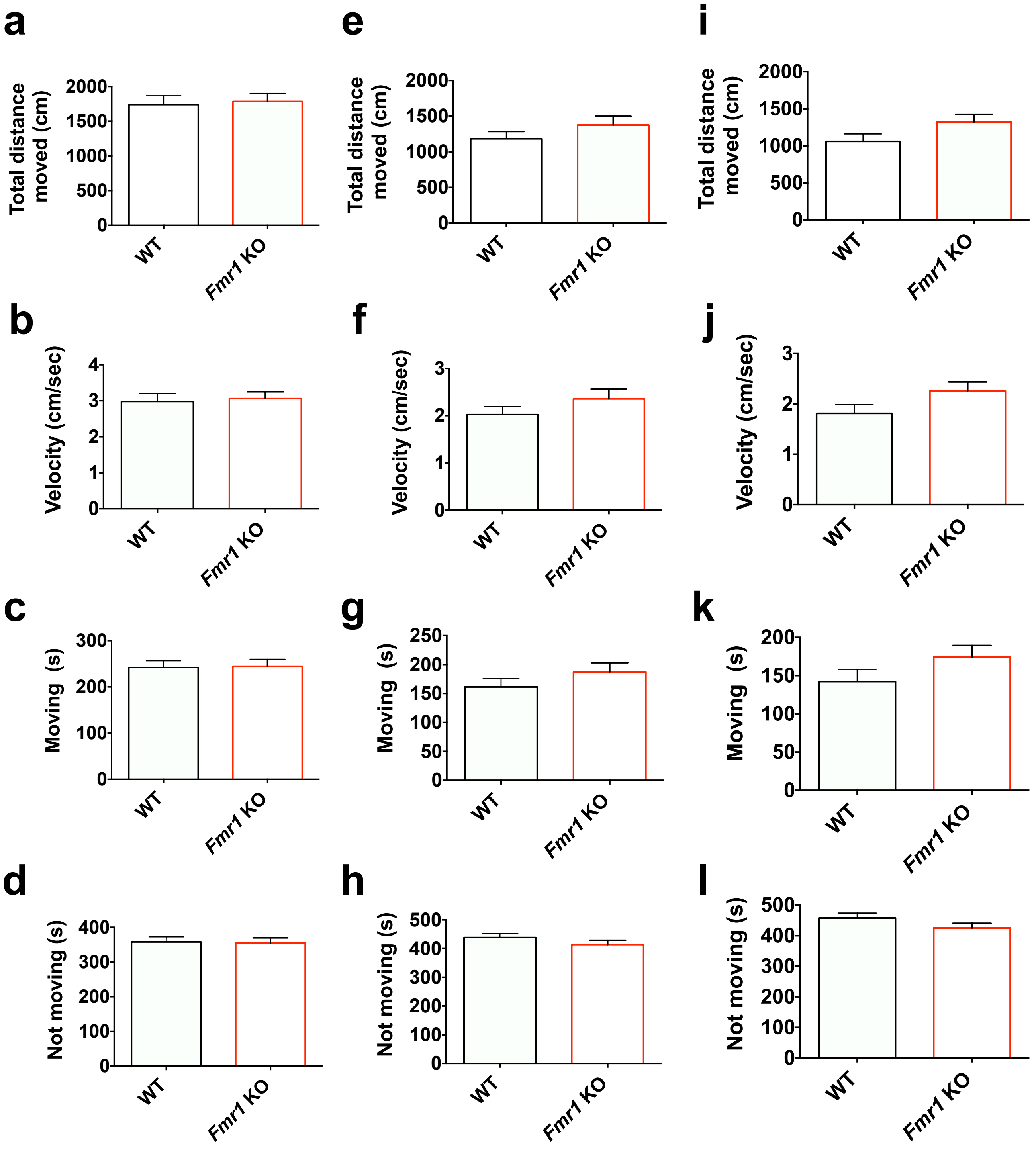

Supplementary Figure 8. Exploratory activity of WT and *Fmr1* KO mice in the L-maze during the novel object recognition task. (a, b, c, d) Exploratory activity of WT and *Fmr1* KO mice during the habituation phase (day 1 of the protocol) of the novel object recognition task. *Fmr1* KO mice do not show an increased hyperactivity compared with WT mice. **(a)** Total distance moved (WT, 1740 ± 128 cm, n = 8; *Fmr1* KO, 1725 ± 106.8 cm, n = 8; $P = 0.929$, t = 0.090, df = 14 by unpaired Student's t-test). **(b)** Velocity (WT, 2.979 ± 0.219 cm/s, n = 8; *Fmr1* KO, 2.953 ± 0.183 cm/s, n = 8; $P = 0.929$, t = 0.089, df = 14 by unpaired Student's t-test). **(c)** Time spent moving (WT, 242.1 ± 14.66 s, n = 8; *Fmr1* KO, 236.6 ± 13.68 s, n = 8; $P = 0.789$, t = 0.272, df = 14 by unpaired Student's t-test). **(d)** Time spent not moving (WT, 357.9 ± 14.66 s, n = 8; *Fmr1* KO, 363.3 ± 13.67 s, n = 8; $P = 0.793$, t = 0.269, df = 14 by unpaired Student's t-test). **(e, f, g, h)** Exploratory activity of WT and *Fmr1* KO mice during the training phase (day 2 of the protocol) of the novel object recognition task. *Fmr1* KO mice do not show an increased hyperactivity compared with WT mice. **(e)** Total distance moved (WT, 1182 ± 99.72 cm, n = 8; *Fmr1* KO, 1375 ± 123.1 cm, n = 8; $P = 0.244$, t = 1.217, df = 14 by unpaired Student's t-test). **(f)** Velocity (WT, 2.023 ± 0.171 cm/s, n = 8; *Fmr1* KO, 2.353 ± 0.211 cm/s, n = 8; $P = 0.244$, t = 1.217, df = 14 by unpaired Student's t-test). **(g)** Time spent moving (WT, 161.3 ± 14.09 s, n = 8; *Fmr1* KO, 187.1 ± 16.18 s, n = 8; $P = 0.249$, t = 1.203, df = 14 by unpaired Student's t-test). **(h)** Time spent not moving (WT, 438.7 ± 14.09 s, n = 8; *Fmr1* KO, 412.9 ± 16.18 s, n = 8; $P = 0.249$, t = 1.203, df = 14 by unpaired Student's t-test). **(i, j, k, l)** Exploratory activity of WT and *Fmr1* KO mice during the testing phase (day 3 of the protocol) of the novel object recognition task. *Fmr1* KO mice do not show an increased hyperactivity compared with WT mice. **(i)** Total distance moved (WT, 1059 ± 100 cm, n = 8; *Fmr1* KO, 1263 ± 95.77 cm, n = 8; $P = 0.163$, t = 1.474, df = 14 by unpaired Student's t-test). **(j)** Velocity (WT, 1.814 ± 0.171 cm/s, n = 8; *Fmr1* KO, 2.163 ± 0.164 cm/s, n = 8; $P = 0.163$, t = 1.474, df = 14 by unpaired Student's t-test). **(k)** Time spent moving (WT, 142.3 ± 16.09 s, n = 8; *Fmr1* KO, 167.1 ± 14.55 s, n = 8; $P = 0.272$, t = 1.143, df = 14 by unpaired Student's t-test). **(l)** Time spent not moving (WT, 457.9 ± 16.09 s, n = 8; *Fmr1* KO, 432.9 ± 14.55 s, n = 8; $P = 0.272$, t = 1.144, df = 14 by unpaired Student's t-test).

Supplementary Figure 9.

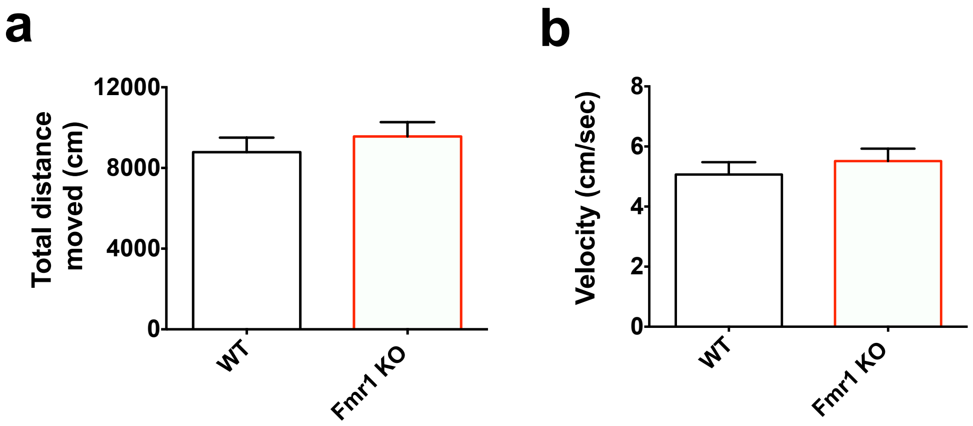

Supplementary Figure 9. Open field activity is identical for WT and *Fmr1* KO mice. **(a)** and **(b)** *Fmr1* KO mice do not show any difference in the open field activity when compared with WT mice (total distance moved**,** WT, 8785 ± 721.9 cm, n = 10; *Fmr1* KO, 9563 ± 708.7 cm, n = 10; *P* = 0.451, t = 0.77, df = 18 by unpaired Student's t-test; velocity WT, 5.07 ± 0.41 cm/s, n = 10; *Fmr1* KO, 5.51 ± 0.123 cm/s, n = 10; *P* = 0.452, t = 0.77, df = 18 by unpaired Student's t-test).

Supplementary Figure 10.

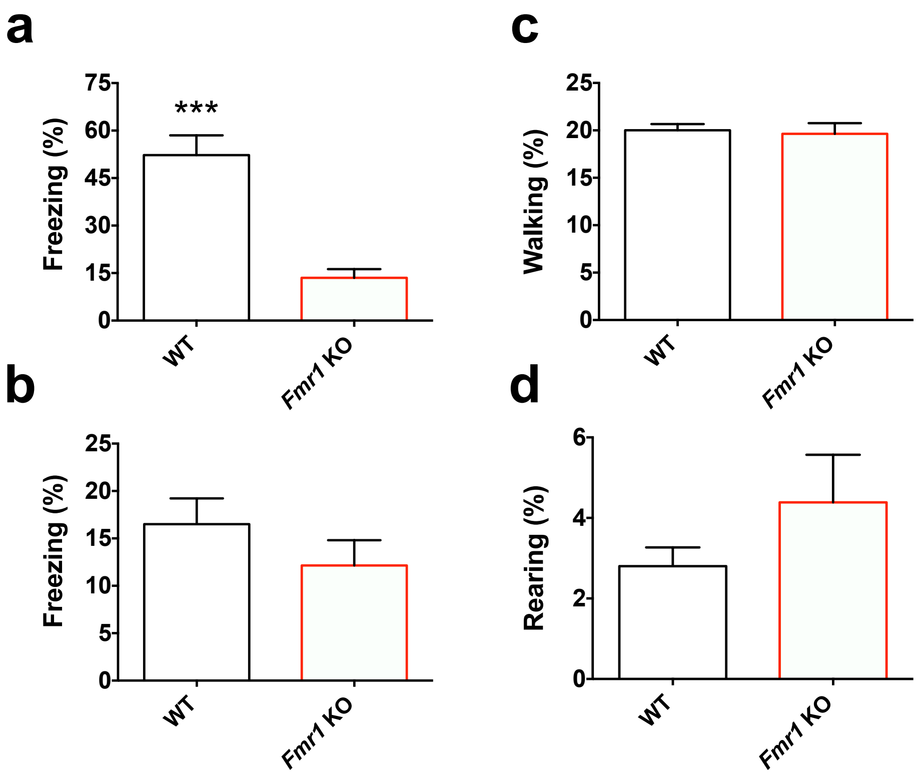

Supplementary Figure 10. Contextual fear conditioning task for WT and *Fmr1* KO mice. (a) Percentage of freezing during the testing phase (second day of the task). *Fmr1* KO mice show a lower percentage of freezing time when compared with WT mice (WT, 52.26 ± 6.246 %, n = 9; *Fmr1* KO, 13.49 ± 2.775 %, n = 11; ***$P < 0.001$, t = 6.05, df = 18 by unpaired Student's t-test). **(b)** Percentage of freezing during the conditioning phase (first day of the task). *Fmr1* KO mice do not show any difference when compared with WT mice (WT, 16.50 ± 2.719 %, n = 8; *Fmr1* KO, 12.15 ± 2.668 %, n = 11; $P = 0.279$, t = 1.117, df = 17 by unpaired Student's t-test). **(c)** and **(d)** Exploratory activity measured as percentage of time spent walking and rearing during the first 2 minutes of the conditioning phase (first day of the task). *Fmr1* KO mice do not show any difference when compared with WT mice (walking, WT, 20.01 ± 0.654 %, n = 9; *Fmr1* KO, 19.64 ± 1.130 %, n = 11; $P = 0.791$, t = 0.268, df = 18 by unpaired Student's t-test; rearing, WT, 2.8 ± 0.467 %, n = 9; *Fmr1* KO, 4.38 ± 1.183 %, n = 11; $P = 0.266$, t = 1.147, df = 18 by unpaired Student's t-test).

Supplementary Figure 11.

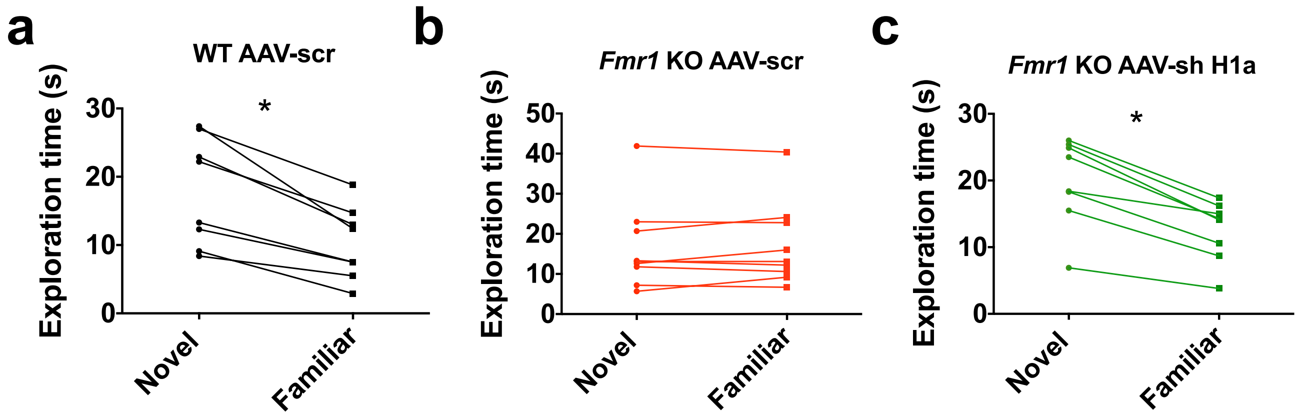

Supplementary Figure 11. Exploration time spent by WT AAV-scr, *Fmr1* KO AAV-scr and *Fmr1* KO AAV-sh H1a mice in the novel object recognition task. Exploration time in the novel object recognition task of WT and *Fmr1* KO mice after bilateral intra-hippocampal microinjection of AAV-sh H1a (300 nl per side) or AAV-scr. The reduction of Homer1a expression resulted in an improvement of cognitive function in *Fmr1* KO mice after AAV-sh H1a injections **(a)** WT AAV-scr exploration time (novel, 17.83 ± 2.793 s, n = 8; familiar, 10.29 ± 1.875 s, n = 8; *$P < 0.05$, t = 2.24, df = 14 by unpaired Student's t-test). **(b)** *Fmr1* KO AAV-scr exploration time (novel, 16.59 ± 3.655 s, n = 9; familiar, 17.23 ± 3.493 s, n = 9; $P = 0.9$, t = 0.13, df = 16 by unpaired Student's t-test). **(c)** *Fmr1* KO AAV-sh H1a (novel, 19.86 ± 2.309 s, n = 8; familiar, 12.51 ± 1.601 s, n = 8; *$P < 0.05$, t = 2.62, df = 12.47 by unpaired Student's t-test).

Supplementary Figure 12.

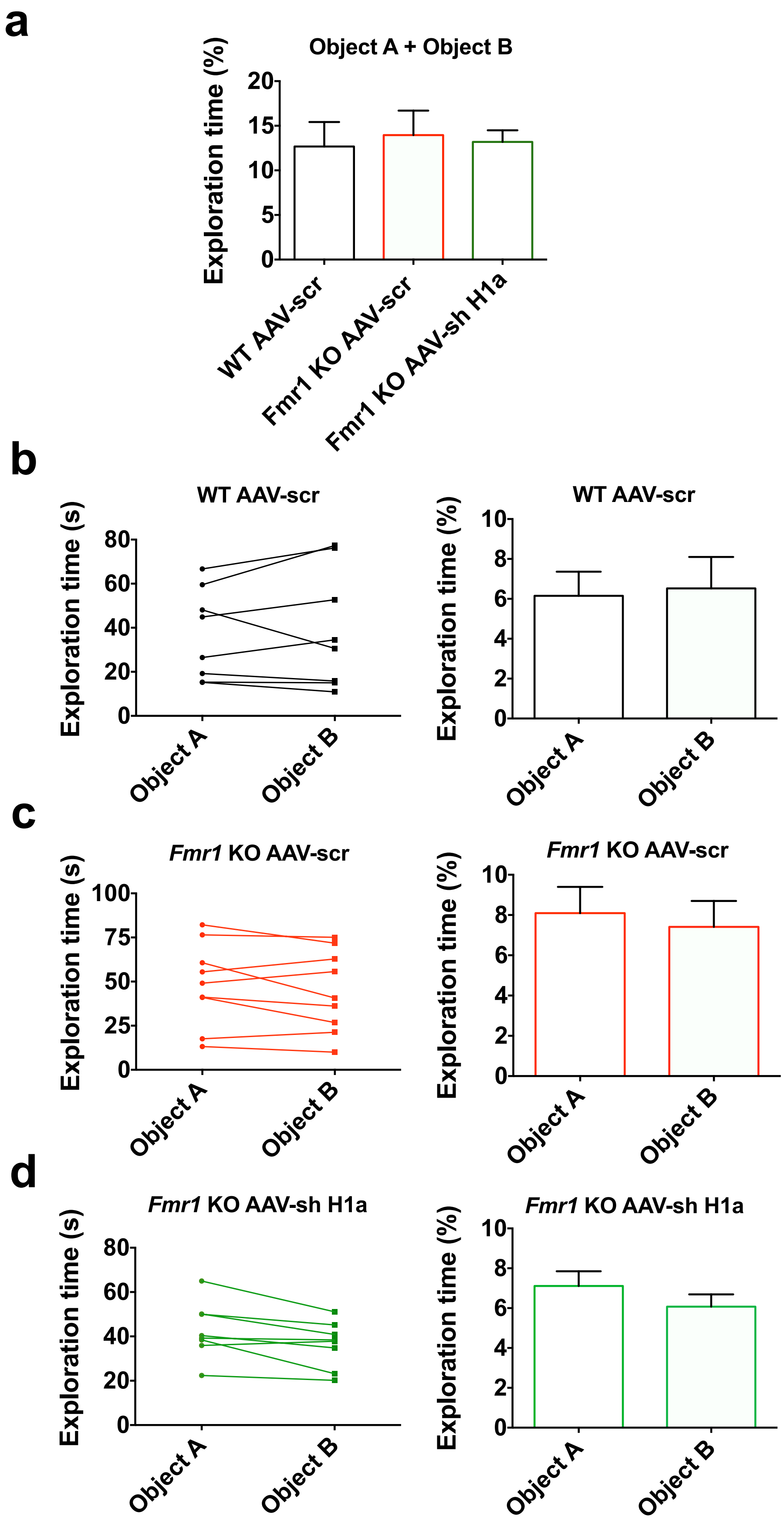

Supplementary Figure 12. Exploration time spent by WT AAV-scr, *Fmr1* KO AAV-scr and *Fmr1* KO AAV-sh H1a mice during the training session (2 identical objects) of the novel object recognition task. **(a)** Objects exploration: No difference was observed between groups in the exploration of object A + object B (WT AAV-scr, 12.68 ± 2.74 %, n = 8; *Fmr1* KO AAV-scr, 13.96 ± 2.74 %, n = 9; *Fmr1* KO AAV-sh H1a, 13.19 ± 1.309 %, n = 8; $F_{(2, 23)} = 0.072$, $P = 0.931$ by one-way ANOVA). **(b)** Exploration time (***left panel***) and percentage of exploration time (***right panel***) spent by WT AAV-scr mice (exploration time, object A, 36.93 ± 7.254 s, n = 8; object B, 39.13 ± 9.48 s, n = 8; $P = 0.856$, t = 0.19, df = 14 by unpaired Student's t-test; percentage of exploration time, object A, 6.154 ± 1.209 %, n = 8; object B, 6.521 ± 1.58 %, n = 8; $P = 0.856$, t = 0.19, df = 14 by unpaired Student's t-test). **(c)** Exploration time (***left panel***) and percentage of exploration time (***right panel***) spent by *Fmr1* KO AAV-scr mice (exploration time, object A, 48.56 ± 7.842 s, n = 9; object B, 44.48 ± 7.7 s, n = 9; $P = 0.715$, t = 0.37, df = 16 by unpaired Student's t-test; percentage of exploration time, object A, 8.093 ± 1.307 %, n = 9; object B, 7.413 ± 1.283 %, n = 9; $P = 0.715$, t = 0.37, df = 16 by unpaired Student's t-test). **(d)** Exploration time (***left panel***) and percentage of exploration time (***right panel***) spent by *Fmr1* KO AAV-sh H1a mice (exploration time, object A, 42.68 ± 4.431 s, n = 8; object B, 36.45 ± 3.68 s, n = 8; $P = 0.298$, t = 1.08, df = 14 by unpaired Student's t-test; percentage of exploration time, object A, 7.113 ± 0.739 %, n = 8; object B, 6.075 ± 0.614 %, n = 8; $P = 0.298$, t = 1.08, df = 14 by unpaired Student's t-test).

Supplementary Figure 13.

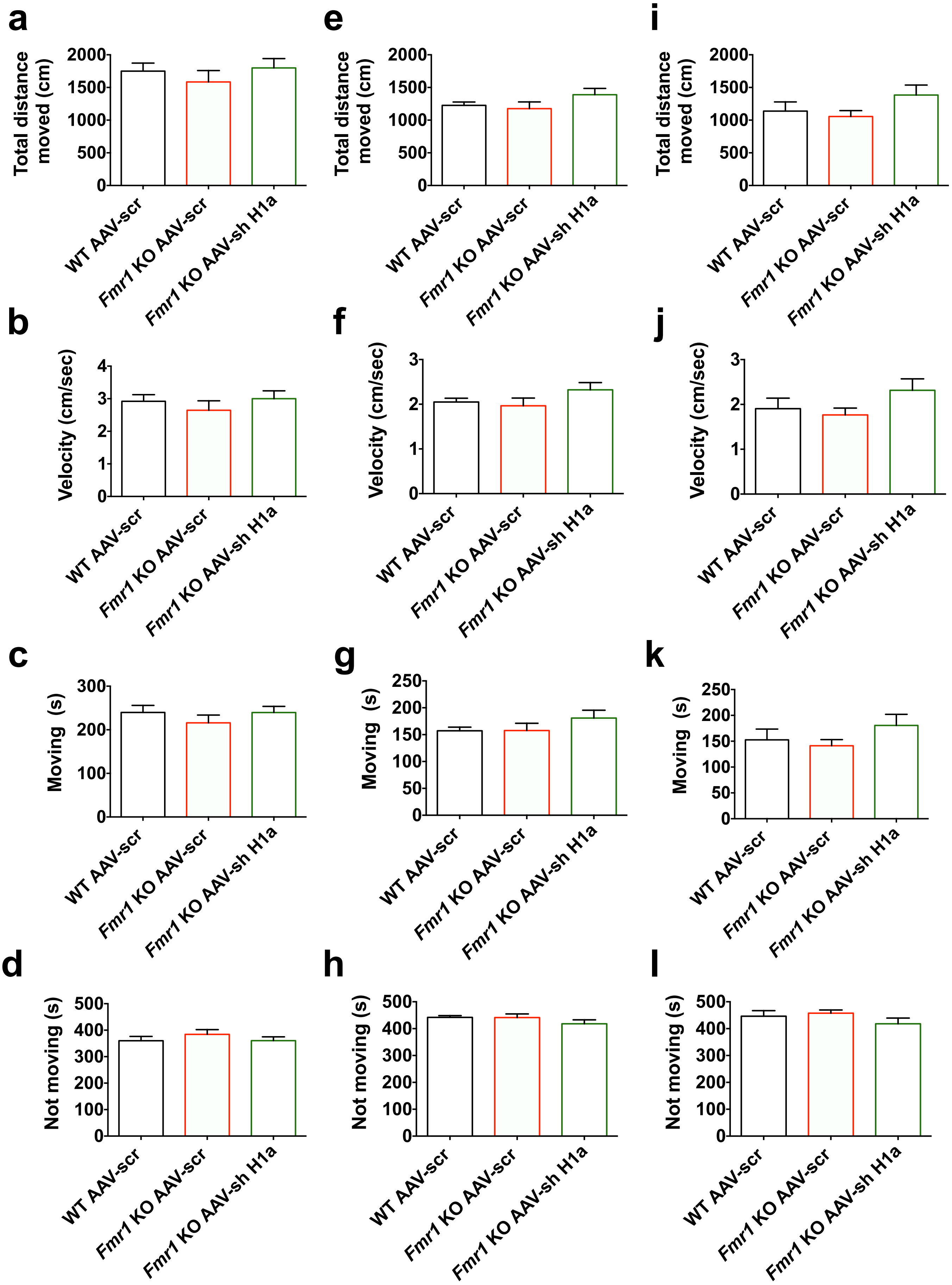

Supplementary Figure 13. Exploratory activity of WT AAV-scr, *Fmr1* KO AAV-scr and *Fmr1* KO AAV-sh H1a mice in the L-maze during the novel object recognition task. (a, b, c, d) Exploratory activity of WT AAV-scr, *Fmr1* KO AAV-scr and *Fmr1* KO AAV-sh H1a mice during the habituation phase (day 1 of the protocol) of the novel object recognition task. No difference was observed between groups in the exploratory activity. **(a)** Total distance moved (WT AAV-scr, 1750 ± 122.6 cm, n = 8; *Fmr1* KO AAV-scr, 1585 ± 173.6 cm, n = 9; *Fmr1* KO AAV-sh H1a, 1799 ± 143.3 cm, n = 8); WT AAV-scr versus *Fmr1* KO AAV-scr P = 0.72 by one-way ANOVA test with Tukey's multiple comparison test; WT AAV-scr versus *Fmr1* KO AAV-sh H1a P = 0.973 by one-way ANOVA test with Tukey's multiple comparison test; *Fmr1* KO AAV-scr versus *Fmr1* KO AAV-sh H1a P = 0.579 by one-way ANOVA test with Tukey's multiple comparison test. **(b)** Velocity (WT AAV-scr, 2.921 ± 0.205 cm/s, n = 8; *Fmr1* KO AAV-scr, 2.646 ± 0.29 cm/s, n = 9; *Fmr1* KO AAV-sh H1a, 3.002 ± 0.24 cm/s, n = 8); WT AAV-scr versus *Fmr1* KO AAV-scr P = 0.72 by one-way ANOVA test with Tukey's multiple comparison test; WT AAV-scr versus *Fmr1* KO AAV-sh H1a P = 0.973 by one-way ANOVA test with Tukey's multiple comparison test; *Fmr1* KO AAV-scr versus *Fmr1* KO AAV-sh H1a P = 0.578 by one-way ANOVA test with Tukey's multiple comparison test. **(c)** Time spent moving (WT AAV-scr, 239.8 ± 16.14 s, n = 8; *Fmr1* KO AAV-scr, 215.9 ± 17.87 s, n = 9; *Fmr1* KO AAV-sh H1a, 239.5 ± 14.21 s, n = 8); WT AAV-scr versus *Fmr1* KO AAV-scr P = 0.556 by one-way ANOVA test with Tukey's multiple comparison test; WT AAV-scr versus *Fmr1* KO AAV-sh H1a P > 0.999 by one-way ANOVA test with Tukey's multiple comparison test; *Fmr1* KO AAV-scr versus *Fmr1* KO AAV-sh H1a P = 0.564 by one-way ANOVA test with Tukey's multiple comparison test. **(d)** Time spent not moving (WT AAV-scr, 360.2 ± 16.14 s, n = 8; *Fmr1* KO AAV-scr, 384.2 ± 17.87 s, n = 9; *Fmr1* KO AAV-sh H1a, 360.5 ± 14.21 s, n = 8); WT AAV-scr versus *Fmr1* KO AAV-scr P = 0.556 by one-way ANOVA test with Tukey's multiple comparison test; WT AAV-scr versus *Fmr1* KO AAV-sh H1a P > 0.999 by one-way ANOVA test with Tukey's multiple comparison test; *Fmr1* KO AAV-scr versus *Fmr1* KO AAV-sh H1a P = 0.564 by one-way ANOVA test with Tukey's multiple comparison test. **(e, f, g, h)** Exploratory activity of WT AAV-scr, *Fmr1* KO AAV-scr and *Fmr1* KO AAV-sh H1a mice during the training phase (day 2 of the protocol) of the novel object recognition task. No difference was observed between groups in the exploratory activity. **(e)** Total distance moved (WT AAV-scr, 1226 ± 51.11 cm, n = 8; *Fmr1* KO AAV-scr, 1176 ± 102.9 cm, n = 9; *Fmr1* KO AAV-sh H1a, 1389 ± 93.36 cm, n = 8); WT AAV-scr versus *Fmr1* KO AAV-scr P = 0.913 by one-way ANOVA test with Tukey's multiple comparison test; WT AAV-scr versus *Fmr1* KO AAV-sh H1a P = 0.42 by one-way ANOVA test with Tukey's multiple comparison test; *Fmr1* KO AAV-scr versus *Fmr1* KO AAV-sh H1a P = 0.218 by one-way ANOVA test with Tukey's multiple comparison test. **(f)** Velocity (WT AAV-scr, 2.050 ± 0.0853 cm/s, n = 8; *Fmr1* KO AAV-scr, 1.966 ± 0.172 cm/s, n = 9; *Fmr1* KO AAV-sh H1a, 2.323 ± 0.161 cm/s, n = 8); WT AAV-scr versus *Fmr1* KO AAV-scr P = 0.914 by one-way ANOVA test with Tukey's multiple comparison test; WT AAV-scr versus *Fmr1* KO AAV-sh H1a P = 0.42 by one-way ANOVA test with Tukey's multiple comparison test; *Fmr1* KO AAV-scr versus *Fmr1* KO AAV-sh H1a P = 0.219 by one-way ANOVAa test with Tukey's multiple comparison test. **(g)** Time spent moving (WT AAV-scr, 157.2 ± 6.695 s, n = 8; *Fmr1* KO AAV-scr,

157.6 ± 13.57 s, n = 9; *Fmr1* KO AAV-sh H1a, 180.8 ± 14.65 s, n = 8); WT AAV-scr versus *Fmr1* KO AAV-scr *P* = 0.999 by one-way ANOVA test with Tukey's multiple comparison test; WT AAV-scr versus *Fmr1* KO AAV-sh H1a *P* = 0.395 by one-way ANOVA test with Tukey's multiple comparison test; *Fmr1* KO AAV-scr versus *Fmr1* KO AAV-sh H1a *P* = 0.386 by one-way ANOVA test with Tukey's multiple comparison test. **(h)** Time spent not moving (WT AAV-scr, 441.8 ± 6.693 s, n = 8; *Fmr1* KO AAV-scr, 441.3 ± 13.54 s, n = 9; *Fmr1* KO AAV-sh H1a, 418 ± 14.65 s, n = 8); WT AAV-scr versus *Fmr1* KO AAV-scr *P* = 0.999 by one-way ANOVA test with Tukey's multiple comparison test; WT AAV-scr versus *Fmr1* KO AAV-sh H1a *P* = 0.39 by one-way ANOVA test with Tukey's multiple comparison test; *Fmr1* KO AAV-scr versus *Fmr1* KO AAV-sh H1a *P* = 0.386 by one-way ANOVA test with Tukey's multiple comparison test. **(i, j, k, l)** Exploratory activity of WT AAV-scr, *Fmr1* KO AAV-scr and *Fmr1* KO AAV-sh H1a mice during the testing phase (day 3 of the protocol) of the novel object recognition task. No difference was observed between groups in the exploratory activity. **(i)** Total distance moved (WT AAV-scr, 1138 ± 141.1 cm, n = 8; *Fmr1* KO AAV-scr, 1056 ± 90.17 cm, n = 9; *Fmr1* KO AAV-sh H1a, 1384 ± 152.5 cm, n = 8); WT AAV-scr versus *Fmr1* KO AAV-scr *P* = 0.892 by one-way ANOVA test with Tukey's multiple comparison test; WT AAV-scr versus *Fmr1* KO AAV-sh H1a *P* = 0.394 by one-way ANOVA test with Tukey's multiple comparison test; *Fmr1* KO AAV-scr versus *Fmr1* KO AAV-sh H1a *P* = 0.184 by one-way ANOVA test with Tukey's multiple comparison test. **(j)** Velocity (WT AAV-scr, 1.904 ± 0.0236 cm/s, n = 8; *Fmr1* KO AAV-scr, 1.766 ± 0.151 cm/s, n = 9; *Fmr1* KO AAV-sh H1a, 2.316 ± 0.255 cm/s, n = 8); WT AAV-scr versus *Fmr1* KO AAV-scr *P* = 0.891 by one-way ANOVA test with Tukey's multiple comparison test; WT AAV-scr versus *Fmr1* KO AAV-sh H1a *P* = 0.394 by one-way ANOVA test with Tukey's multiple comparison test; *Fmr1* KO AAV-scr versus *Fmr1* KO AAV-sh H1a *P* = 0.184 by one-way ANOVA test with Tukey's multiple comparison test. **(k)** Time spent moving (WT AAV-scr, 152.7 ± 20.77 s, n = 8; *Fmr1* KO AAV-scr, 141.2 ± 11.89 s, n = 9; *Fmr1* KO AAV-sh H1a, 180.6 ± 21.47 s, n = 8); WT AAV-scr versus *Fmr1* KO AAV-scr *P* = 0.895 by one-way ANOVA test with Tukey's multiple comparison test; WT AAV-scr versus *Fmr1* KO AAV-sh H1a *P* = 0.544 by one-way ANOVA test with Tukey's multiple comparison test; *Fmr1* KO AAV-scr versus *Fmr1* KO AAV-sh H1a *P* = 0.289 by one-way ANOVA test with Tukey's multiple comparison test. **(l)** Time spent not moving (WT AAV-scr, 446.2 ± 20.81 s, n = 8; *Fmr1* KO AAV-scr, 457.5 ± 12 s, n = 9; *Fmr1* KO AAV-sh H1a, 418.1 ± 21.48 s, n = 8). WT AAV-scr versus *Fmr1* KO AAV-scr *P* = 0.896 by one-way ANOVA test with Tukey's multiple comparison test; WT AAV-scr versus *Fmr1* KO AAV-sh H1a *P* = 0.541 by one-way ANOVA test with Tukey's multiple comparison test; *Fmr1* KO AAV-scr versus *Fmr1* KO AAV-sh H1a *P* = 0.288 by one-way ANOVA test with Tukey's multiple comparison test.

Supplementary Figure 14.

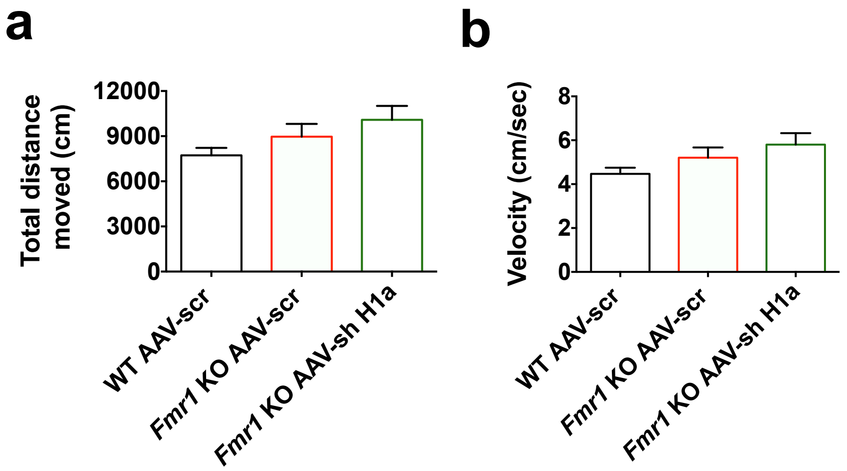

Supplementary Figure 14. Open field activity is identical for WT AAV-scr, *Fmr1* KO AAV-scr and *Fmr1* KO AAV-sh H1a mice. Open field activity of WT and *Fmr1* KO mice after bilateral intra-hippocampal microinjection of AAV-sh H1a or AAV-scr (in both conditions 300 nl per side). **(a)** No difference was observed between groups in the total distance moved (WT AAV-scr, 7728 ± 494.2 cm, n = 11; *Fmr1* KO AAV-scr, 8965 ± 854.5 cm, n = 9; *Fmr1* KO AAV-sh H1a, 10088 ± 920.7 cm, n = 9; $F_{(2, 26)}$ = 2.570, P = 0.096 by one-way ANOVA). **(b)** No difference was observed between groups in the velocity (WT AAV-scr, 4.468 ± 0.284 cm/s, n = 11; *Fmr1* KO AAV-scr, 5.204 ± 0.467 cm/s, n = 9; *Fmr1* KO AAV-sh H1a, 5.804 ± 0.52 cm/s, n = 9; $F_{(2, 23)}$ = 2.646, P = 0.089 by one-way ANOVA).

Supplementary Figure 15.

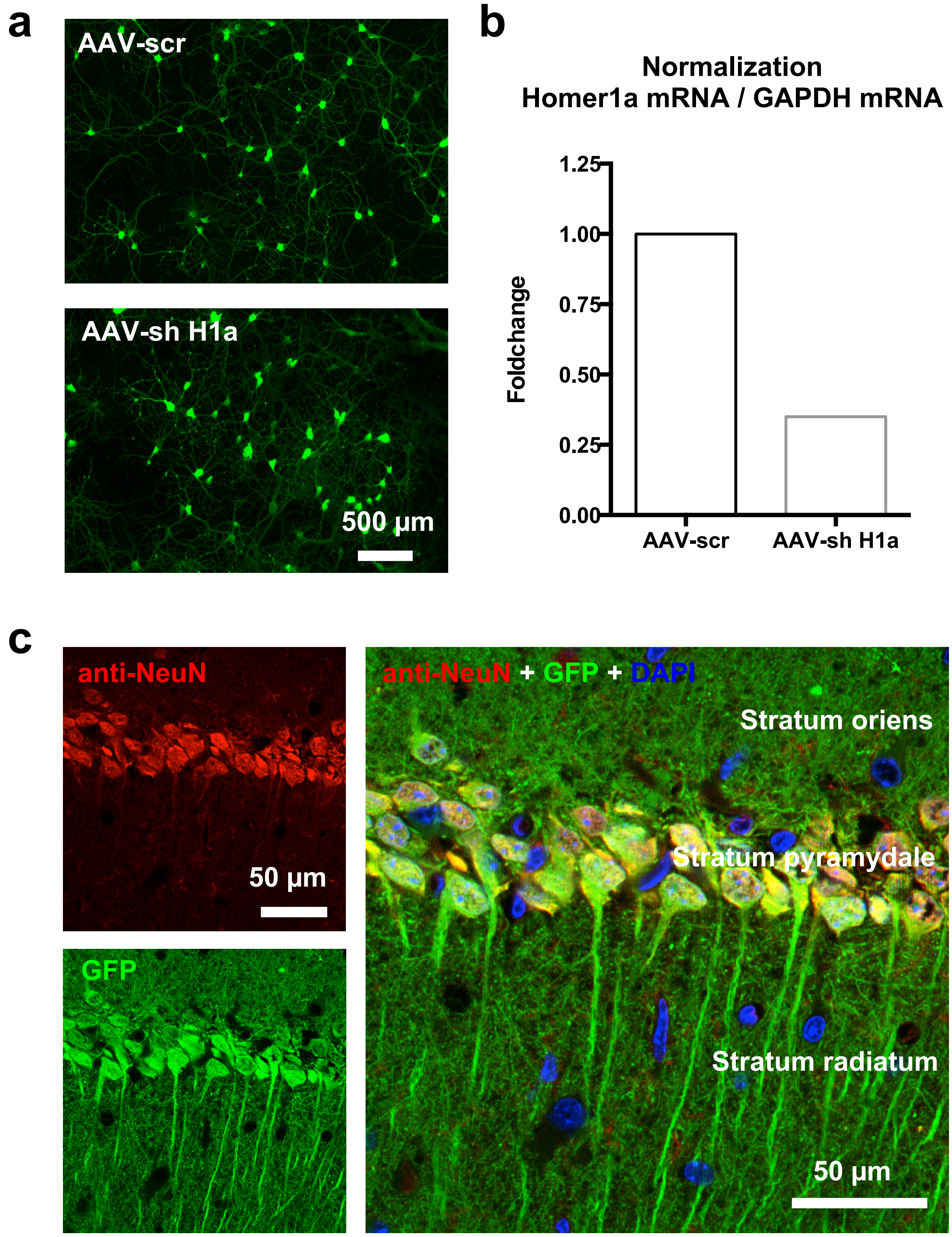

Supplementary Figure 15. Validation of the AAV viruses and efficiency of their infection. (a, b) *In vitro* validation of the rAAV-mediated gene silencing (the recombinant AAVs are composed of a biscistronic expression unit permitting the expression of short hairpin RNA, driven by the mouse RNA polymerase III U6 promoter and GFP reporter, under the control of the CBA promoter). **(a)** Microscopy images obtained from fixed neurons, 4 days following infection (with either AAV-scr and AAV-sh H1a) demonstrate an

equivalent level of infection of cultured hippocampal neurons (as shown by expression of GFP reporter). (**b)** The expression level of Homer1a mRNA was reduced by 65 % in AAV-sh H1a infected neurons compared to those infected with AAV-scr, when normalized to GAPDH mRNA (68% following normalisation to Nono mRNA). Four days after infection the primary infected-neurons were harvested and qPCR was performed using a LightCycler® 480 Real-Time PCR System (Roche, Meylan, France). QPCR reactions were done in duplicate for each sample, using transcript-specific primers for mouse Homer1a. Relative expression analysis was corrected for PCR efficiency and normalized against two reference genes (mGADPH, Fwd: 5′ TCA AGA AGG TGG TGA AGC AG 3′ and Rev: 5′ TGG GAG TTG CTG TTG AAG TC 3′) and mNono, Fwd 5’ CTG TCT GGT GCA TTC CTG AAC TAT3’ and Rev 5’ AGC TCT GAG TTC ATT TTC CCA TG 3’). The relative level of expression was calculated using the comparative ($2^{-\Delta\Delta CT}$) method and controls were arbitrarily set at 1**.** **(c)** Anti-NeuN immunostaining was carried out on free-floating vibratome slices prepared from 4% paraformaldehyde-perfused mouse brains, 9 weeks after bilateral intra-hippocampal microinjection of AAV-sh H1a or AAV-scr (300 nl per side). This analysis revealed that AAVs infected mostly neurons in the *stratum pyramidale* of CA1 subregion (97.3%, n = 219 neurons from four independent injection from two different brains).

Supplementary Figure 16.

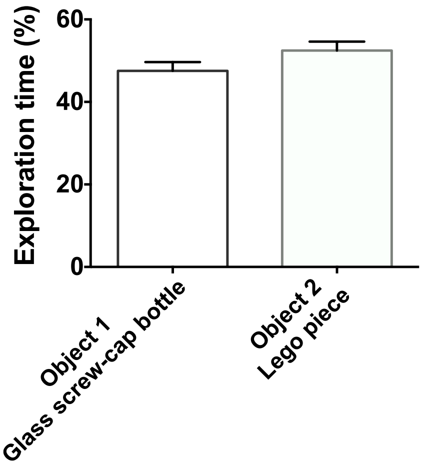

Supplementary Figure 16. Validation of objects used in the novel object recognition task. Validation of the objects using a distinct, behaviorally-naive group of WT mice of the same genetic background. Objects presented were as follows: 1) glass screw-cap bottle (6.8 cm tall, approximate diameter 1.9 cm) composed of amber glass and plastic screw cap lid, and 2) plastic Lego piece (3.3 x 3.3 x 2.5 cm). The mice did not show any preference bias for the selected objects. Data is presented as % of time exploring each object normalized to total exploration time (Object 1- Glass screw-cap bottle, 47.53 ± 2.14, n = 9; Object 2 - Lego piece, 52.47 ± 2.14, n = 9; P = 0.123, t = 1.63, df = 16 by unpaired Student's t-test).

Supplementary Table 1.

Reference	Species	Culture type	DIV	NMDAR subunit	Synaptic Diff. ($\mu m^2/s$)	Recombinant/native
Groc et al., 2004 *Nat Neurosci*	Rat (E18)	Banker cultures	9-11 DIV	GluN1-NMDAR (not QD)	0.021 median (± IQR)	Native
#Aloisi et al.,	Mouse (E18)	Mixed cultures	12-15 DIV	GluN1-NMDAR	0.071 median (± IQR)	Native
Groc et al., 2006 *PNAS*	Rat (E18)	Banker cultures	11-16 DIV	GluN2B-NMDAR	0.05 median (± IQR)	Native
Dupuis et al., 2014 *EMBO J*	Rat (E18)	Mixed cultures	14-20 DIV	GluN2B-NMDAR	*0.031 median (± IQR)	Native
Bard et al., 2010 *PNAS*	Rat (E18)	Banker cultures	15-20 DIV	GluN2A-NMDAR	0.11 median (± IQR)	Native
Bard et al., 2010 *PNAS*	Rat (E18)	Banker cultures	15-20 DIV	GluN2B-NMDAR	0.32 median (± IQR)	Native

Supplementary Table 1. Comparison of diffusion values for NMDA receptors across several studies (inclusion criteria: native receptors, synaptic compartment only, both mobile and immobile receptor fraction, median values).

#Our study. Here values are reported as a median, to be compared with the other studies

*Values in paper are reported as mean ± sem (0.03 ± 0.015 µm2/s); median value given in the table is by personal communication from the first author of this study, J. Dupuis (median ± IQR, 0.03113 ± 0.008-0.0574 µm2/s).

Supplementary References

1. Michaluk, P. *et al.* Matrix metalloproteinase-9 controls NMDA receptor surface diffusion through integrin beta1 signaling. *J. Neurosci.* **29**, 6007-6012 (2009).
2. Opazo, P. *et al.* CaMKII triggers the diffusional trapping of surface AMPARs through phosphorylation of stargazin. *Neuron* **67**, 239-252 (2010).
3. Ladépêche, L. *et al.* Single-molecule imaging of the functional crosstalk between surface NMDA and dopamine D1 receptors. *Proc. Natl. Acad. Sci. U.S.A* **110**, 18005-18010 (2013).
4. Zhang, H. *et al.* Regulation of AMPA receptor surface trafficking and synaptic plasticity by a cognitive enhancer and antidepressant molecule. *Mol. Psychiatry* **18**, 471-484 (2013).

5 de Vrij, F.M., *et al.* Rescue of behavioral phenotype and neuronal protrusion morphology in Fmr1 KO mice. *Neurobiol Dis.* **31**, 127-32 (2008).

6 Tabet R., Vitale N., & Moine H. Fragile X syndrome: Are signaling lipids the missing culprits? *Biochimie.* **130**, 188-194 (2016)

7 .Pilpel Y., *et al.* Synaptic ionotropic glutamate receptors and plasticity are developmentally altered in the CA1 field of Fmr1 knockout mice. *J Physiol.* **587**, 787-804 (2009).

8 Jung K.M., *et al.* Uncoupling of the endocannabinoid signalling complex in a mouse model of fragile X syndrome. *Nat Commun.* **3**, 1080 (2012).

9 Wijetunge L.S., *et al.* Stimulated emission depletion (STED) microscopy reveals nanoscale defects in the developmental trajectory of dendritic spine morphogenesis in a mouse model of fragile X syndrome. *J Neurosci.* **34**, 6405-12. (2014)

10 Haberl M.G., *et al.* Structural-functional connectivity deficits of neocortical circuits in the Fmr1(-/y) mouse model of autism. *Sci Adv.* **1**:e1500775 (2015).

11 Vinueza Veloz M.F., *et al.* The effect of an mGluR5 inhibitor on procedural memory and avoidance discrimination impairments in Fmr1 KO mice. *Genes Brain Behav.* 11, 325-31 (2012).

12 Gantois I., *et al.* Chronic administration of AFQ056/Mavoglurant restores social behaviour in Fmr1 knockout mice. *Behav Brain Res.* **15**, 239:72-9 (2013).

13 Mao S.C., *et al.* Inhibition of spontaneous recovery of fear by mGluR5 after prolonged extinction training. *PLoS One* **8**, e59580 (2013).

14 de Esch C.E., *et al.* Fragile X mice have robust mGluR5-dependent alterations of social behaviour in the Automated Tube Test. *Neurobiol Dis.* Mar;75:31-9 (2015).

15 Groc, L. *et al.* Differential activity-dependent regulation of the lateral mobilities of AMPA and NMDA receptors. *Nat. Neurosci.* **7**, 695-696 (2004).

16 Michaluk, P. *et al.* Matrix metalloproteinase-9 controls NMDA receptor surface diffusion through integrin beta1 signaling. *J. Neurosci.* **29**, 6007-6012 (2009).

17 Opazo, P. *et al.* CaMKII triggers the diffusional trapping of surface AMPARs through phosphorylation of stargazin. *Neuron* **67**, 239-252 (2010).

18 Ladépêche, L. *et al.* Single-molecule imaging of the functional crosstalk between surface NMDA and dopamine D1 receptors. *Proc. Natl. Acad. Sci. U.S.A* **110**, 18005-18010 (2013).

19 Zhang, H. *et al.* Regulation of AMPA receptor surface trafficking and synaptic plasticity by a cognitive enhancer and antidepressant molecule. *Mol. Psychiatry* **18**, 471-484 (2013).

20 Aurnhammer, C. *et al.* Universal real-time PCR for the detection and quantification of adeno-associated virus serotype 2-derived inverted terminal repeat sequences. *Hum. Gene Ther. Method.* **23**, 18-28 (2012).

21 Paxinos, G. & Franklin, K.B.J. *The mouse brain in stereotaxic coordinates*. 2. (San Diego, CA: Academic, 2001).

22 During, M.J., Young, D., Baer, K., Lawlor, P. & Klugmann, M. Development and optimization of adeno-associated virus vector transfer into the central nervous system. *Methods Mol. Med.* **76**, 221-236 (2003).

23 Myme, C.I., Sugino, K., Turrigiano, G.G. & Nelson, S.B. The NMDA-to-AMPA ratio at synapses onto layer 2/3 pyramidal neurons is conserved across prefrontal and visual cortices. *J. Neurophysiol.* **90**, 771-779 (2003).
