# Supplementary material for: Altered surface mGluR5 dynamics provoke synaptic NMDAR dysfunction and cognitive defects in Fmr1 knockout mice
Source: Nat Commun. 2017 Oct 24;8:1103. doi: 10.1038/s41467-017-01191-2 (PMC5653653; doi:10.1038/s41467-017-01191-2)
Supplement: Supplementary file 2 — Peer Review File [file 41467_2017_1191_MOESM2_ESM.pdf]

Reviewers' comments:

Reviewer #1 (Remarks to the Author):

A. Summary of the key results
The work by Aloisi et al. report a correlation between the dynamic of mGluR5 receptors, NMDAR and the function of the scaffold protein Homer1A. The molecular network has been identified to be involved in the cognitive impairments caused by the fragile X-syndrome. With a combination of single molecule tracking, electrophysiological approaches and behavior experiments the authors demonstrate that slight changes in the dynamic arrangement between synaptic molecules might cause the impairment of LTD induction seen in fragile x-syndrome. The potential beauty of the work is the combination of different techniques to track the line from single molecule dynamics to behavior output.
B. Originality and interest: if not novel, please give references
The work focus on the molecular base for fragile x-syndrome and the reported impairment of LTD. The functional connectivity between mGluR5 and Homer1A has been recently reported (Guo, J. (2016) Neurosci. 36, 2131-). The paper by Aloisi et al. add the aspect of molecular dynamics to the molecular mechanism involved and consequently use appropriate tools to confirm this aspect in electrophysiological and behavioral experiments.
C. Data & methodology: validity of approach, quality of data, quality of presentation
The approach to determine the dynamic of surface receptors follows established protocols, mainly based on quantum dot linked antibodies for extracellular epitopes of GluN1, GluA2 and mGluR5 receptors. As reported before by others or coauthors of this work, diffusion properties of these three receptor populations are quite different, particular within the synaptic compartment (Serge et al. 2002, Groc et al. 2004). To my surprise are NMDA receptors are reported to be approximately ten times faster within synapses than GluA2 receptors, which has been reported to be rather the opposite in previous reports. Particular mGluR5 receptors as well as NMDA receptors with a reported mean diff.-coeff. of ~0.02 and ~0.12 $\mu m^2/s$, respectively, are extremely mobile (compare reported values in Serge et al. 2002, Groc et al. 2004). Within the manuscript there is no discussion about the discrepancy between the reported and former published data. Are their problems with the specificity of antibodies? The antibodies used here are similar to the one used in previous studies? The surface labeling in supplementary figure 6 look very dense and rather unspecific, could this be a reason? For the statistics of the mobility data it would be nice to show distributions averaged between different experiments to report the variability between experiments. One way could be to include the experimental variation in the cumulative representation?
D. Appropriate use of statistics and treatment of uncertainties
Statistics reported for the diffusion coefficients seem particular for the reported difference of NMDA receptor diffusion in WT and FMR1 KO mice problematic. The large number of values (>10000 per group) might bias the strength of the used tests. I would suggest to rather compare the averaged distribution of WT and FMR1-KO group by comparing control experiments from different cultures to knock-out cultures. The cutoff for immobile receptors should be mentioned. For many experiments the number of experiments (cells) and their origin of how many different cultures is not given.
E. Conclusions: robustness, validity, reliability
Despite the mentioned problems above, the multitude of experiments, particular y the use

of interaction side specific peptides, seem to confirm the main conclusion that the interaction between mGluR5 and Homer1A is critical to the functional consequences reported here and by others.
F. Suggested improvements: experiments, data for possible revision
As stated above, I would appreciate a revision of the data given on the dynamics of the involved receptors, particular in respect to previous work. In general the use of surface dynamics of key molecules as transmitter receptors can teach us a lot about molecular mechanisms behind and this work could be another nice example. However, large differences in the dynamics between experiments let wonder whether the method is robust enough to support the conclusions made in the paper.
G. References: appropriate credit to previous work?
Previous work is appropriate cited
H. Clarity and context: lucidity of abstract/summary, appropriateness of abstract, introduction and conclusions
The paper is clear written and results discussed in the context.

Reviewer #2 (Remarks to the Author):

This manuscript addresses further contributions of the mGluR5 receptor in hippocampal synaptic and behavioral phenotypes of Fragile X mutant mice, through a Homer 1a mechanism.

At the end of the Results section, page 7 line 229, mention is made of a "second generation" Fmr1 mouse model. Differences in the mutation and breeding are briefly described later under Animals, page 10 lines 345-347. Apparently the mice used in the present experiments are different than the widely used Fmr1 line of mice. A full description of the new line of mutant mice must appear at the beginning of the manuscript and in the abstract. Rationale for the different type of mutation, how the mutation was generated, and the quality and quantity of genetic differences between the conventional Fmr1 mutation, must be carefully explained. Comparisons of the full set of phenotypic differences between the present mouse and the conventional Fmr1 line must be spelled out. The single reference 61 to a 2006 publication is insufficient.

The novel object recognition task was conducted using non-standard methods. In fact, a spatial component has been added, by placing the second object in a different arm of an L-shaped maze, instead of presenting the first and second objects in adjacent locations within an open field.

This difference is important because Fmr1 mice have been frequently reported to show hyperactivity. If the mutants move throughout the L-shaped arena at higher rates, they are likely to explore both arms more than their WT controls, and concomitantly are likely to spend less total time with the objects.

Similarly, hyperactivity could artifactually reduce freezing time in the contextual fear conditioning test.

The potential confound of higher locomotor activity can be easily evaluated. (a) Number of seconds spent exploring each object should be shown, in addition to the discrimination index, for the novel object recognition phase. (b) Number of seconds spent exploring each of the two identical objects during the familiarization phase should be shown. (c) An independent open field test for exploratory locomotion must be conducted, and presented with standard activity parameters.

There is no explanation about how the behavioral data shown in Figure 10 derives from a different experiment than the behavioral data shown in Figure 9 Panels d and e. Does Supplementary Figure 10 represent uninjected mice of the same genotypes as Figure 9? Or does Supplementary Figure 10 employ the conventional Fmr1 knockout line of mice?

Full statistical results must be added (e.g. F value, degrees of freedom, stringent posthoc analyses). Stating only the p value for ANOVA and t-tests is insufficient.

The authors ignore the very large body of literature showing normal cognitive scores in Fmr1 mice on many learning and memory tasks conducted by many independent labs, including the novel object recognition and fear conditioning assays which were conducted in the present studies. The authors are encouraged to read the behavioral literature more fully.

Absence of the full cognitive datasets, and of the necessary open field control experiment, represent serious flaws in this manuscript.

Reviewer #3 (Remarks to the Author):

This manuscript details interesting experiments investigating the interaction of mGluR5 and NMDA receptors in a mutant mouse model of fragile X (the Fmr1 KO). Specifically, the authors explore the dynamics of receptor diffusion in cultured neurons (using Q-dots), finding that the mobility of mGluR5 and the NMDAR are increased in the Fmr1 KO neurons. No change is seen in the mobility of GluA2, suggesting that the effect is somewhat specific to the mGluR-NMDAR complex. Based on the previous results of the Huber lab, they then show that disrupting mGluR5-Homer binding mimics and occludes the increased receptor trafficking in the Fmr1 KO. Additionally, the co-localization of mGluR5, NMDAR and Homer1 is increased in in the synapses of the Fmr1 KO neurons.

In addition to the in vitro work, the authors examine the whether the NMDAR component of LTD is altered in young (P11-15) hippocampal slices from Fmr1 KO mice. Specifically, they measure NMDAR EPSCs during stimulation of group 1 mGluRs using the agonist DHPG. They find that the NMDAR component of DHPG-LTD is deficient in the Fmr1 KO, and this is mimicked by disruption of the mGluR-Homer interaction. To show that enhancing the

mGluR-Homer interaction could rescue the deficit in NMDAR function in the Fmr1 KO, they injected shRNA to the dominant negative Homer 1a (or scrambled shRNA), and measured the NMDAR component of DHPG-LTD in the Fmr1 KO. They also performed novel object recognition and contextual fear conditioning on injected animals, and show a remarkable rescue of both behvaioral deficits.

Overall, this study is an interesting addition to the literature regarding the regulation of mGluR-NMDAR interaction, and the role of this in the fragile X mouse model. The in vitro Q-dot experiments are particularly compelling. There are, however, a number of drawbacks that should be addressed, particularly with respect to the electrophysiology and behaviour experiments.

1.) The in vitro experiments using Q-dots show a convincing increase in the mobility of mGluR5 and NMDARs in the Fmr1 KO neurons. However, these experiments use Mitotracker labelling to define "synaptic sites" without providing verification using a more conventional synaptic marker (i.e., synaptophysin, PSD-95). This is important because all of the work shown in Figures 5-7 is dependent upon the "synaptic" association. These data would be much more robust if there was validation using another method, and if the total co-localization of mGluR5 and GluN1 was shown (not just the "synaptic" fraction).

2.) The LTD experiments are interesting in light of the specific focus on NMDAR EPSCs. However, there are a few issues. First, the developmental time-point at which these experiments are performed is P11-15, at which point mGluR-LTD is presynaptic (Nosyreva and Huber, 2005). The authors, however, interpret their effects in terms of a postsynaptic mechanism. This should be clarified by including measurements of PPR. These experiments should also be accompanied by standard LTD measurements to ensure that they are indeed inducing DHPG-LTD with their protocol.
3.) In Figure 7, both the mGluR5ct and control peptides seem to have an effect on the Fmr1 KO. This should be discussed. Accordingly, the electrophysiology shown in Figure 8 should include a peptide-treated Fmr1 KO group.

4.) The in vivo injection experiments require a number of controls, including an estimation of the efficiency of the Homer1a knockdown, and WT groups for the electrophysiology experiments.

5.) The behaviour experiments seem to be the weaker parts of this study. The descriptions of the work are not very thorough, and the data appear hastily put together. For example, the methods state that behaviour experiments were only performed in the Fmr1 KO, yet there is data shown for WT. Were these experiments performed blind to genotype? Were the groups age-matched and counterbalanced for treatment? These details are essential for assessing the results, especially since the effects shown are so uncharacteristically large.

Moreover, many of these findings have been previously published. The deficits in contextual fear conditioning have been described previously (Eadie et al. 2012, Auerbach et al. 2011). While it is true that reduction of Homer1a in the Fmr1 KO has not been found to rescue these specific phenotypes, it has been thoroughly studied with respect to other phenotypes

(Huber). Additionally, the deficit in contextual fear conditioning has been attributed to a hypofunction of NMDARs in the dentate gyrus (Eadie et al. 2012). How does this fit with the authors' other findings, which are focused on CA1?

Reviewer #1 (Remarks to the Author):

A. Summary of the key results

The work by Aloisi et al. report a correlation between the dynamic of mGluR5 receptors, NMDAR and the function of the scaffold protein Homer1A. The molecular network has been identified to be involved in the cognitive impairments caused by the fragile X-syndrome. With a combination of single molecule tracking, electrophysiological approaches and behavior experiments the authors demonstrate that slight changes in the dynamic arrangement between synaptic molecules might cause the impairment of LTD induction seen in fragile x-syndrome. The potential beauty of the work is the combination of different techniques to track the line from single molecule dynamics to behavior output.

Response:

We thank the reviewer for this very positive statement.

B. Originality and interest: if not novel, please give references

The work focus on the molecular base for fragile x-syndrome and the reported impairment of LTD. The functional connectivity between mGluR5 and Homer1A has been recently reported (Guo, J. (2016) Neurosci. 36, 2131-). The paper by Aloisi et al. add the aspect of molecular dynamics to the molecular mechanism involved and consequently use appropriate tools to confirm this aspect in electrophysiological and behavioral experiments.

Response:

We thank the reviewer for appreciating the advancement of our work in the context of the present literature in the field. We would like to point out that our paper does not focus on the reported impairment of LTD (LTD of AMPA currents) but demonstrates for the first time that also mGlu-LTD of NMDA currents is impaired in FXS. Indeed, the main novelty of our work consists on the demonstration that the dynamics of mGlu5 receptors and plasticity of NMDA receptors are altered in the FXS model. Both findings are novel and never explored before. In addition, we provide a mechanistic explanation for these alterations, which are both dependent on mGluR5/Homer disruption adding new value and relevance to an emerging pathophysiological mechanism in this disease.

C. Data & methodology: validity of approach, quality of data, quality of presentation

The approach to determine the dynamic of surface receptors follows established protocols, mainly based on quantum dot linked antibodies for extracellular epitopes of GluN1, GluA2 and mGluR5 receptors. As reported before by others or coauthors of this work, diffusion properties of these three receptor populations are quite different, particular within the synaptic compartment (Serge et al. 2002, Groc et al. 2004).

Comment:

To my surprise are NMDA receptors are reported to be approximately ten times faster within synapses than GluA2 receptors, which has been reported to be rather the opposite in previous reports. Particular mGluR5 receptors as well as NMDA receptors with a reported mean diff.-coeff. of ~0.02 and ~0.12 $\mu m^2/s$, respectively, are extremely mobile (compare reported values in Serge et al. 2002, Groc et al. 2004). Within the manuscript there is no discussion about the discrepancy between the reported and former published data.

Response:

The reviewer is right to mention that the diffusion coefficients measured in this study are different from the ones previously reported, although those differences remain moderate (as an example, median diffusion coefficient of 0.084 $\mu m^2/s$ for AMPAR and 0.037 $\mu m^2/s$ for NMDAR in Groc et al., 2004 versus 0.095 $\mu m^2/s$ for AMPAR and 0.11 $\mu m^2/s$ for NMDAR in the present study). Several factors can explain these differences: i) the former data (Sergé et al., 2002; Groc et al., 2004) were obtained in Banker cultures (neurons only) while mixed cultures (neurons and glia together) were used here, ii) the developmental stages were different between studies (3-8 days in vitro for Sergé et al., 2002, 9-11 days in vitro for Groc et al., 2004 versus 12-15 days in vitro in the present study), iii) the species were different (rats in Sergé et al., 2002 and Groc et al., 2004 versus mice here), iv) the frequencies of image acquisitions were also different (25 Hz in Sergé et al., 2002 and 15 Hz in Groc et al., 2004 versus 20 Hz in the present study), as well as v) the culture and recording medium. Each of these factors may account for these differences between studies. However, one should keep in mind that our data match recent publications performed in a similar configuration (see Ladépêche et al., 2013 and Dupuis et al., 2014), that absolute diffusion values should be handled with caution since they depend heavily on the methods used to perform single-particle tracking measurements, and that the same recording parameters were carefully kept along this study to allow unbiased comparison between the different experimental conditions.

Comment:
Are their problems with the specificity of antibodies? The antibodies used here are similar to the one used in previous studies?

Response:
The antibodies used to track AMPAR (mouse monoclonal anti-GluA2 antibody, #MAB387, Millipore) and NMDAR (rabbit polyclonal anti-GluN1 antibody, #AGC-001, Alomone labs) have been already used in many other studies by the groups of Drs. L. Groc and D. Choquet and demonstrated to be specific using various approaches (e.g. Opazo et al., 2010; Ladépêche et al., 2013; Zhang et al., 2013; Dupuis et al., 2014). The specificity of mGluR5 antibody (mouse monoclonal anti-NH_2-mGluR5 antibody) has been previously demonstrated by Mion et al., 2001, using both Western blotting and cell surface immunolabelling under non permeabilized condition. We have now added this information in the Methods section (page 11, paragraph "Single-Particle (Quantum Dot) Tracking and Surface Diffusion Calculation" (Line 385)).

Comment:
The surface labeling in supplementary figure 6 look very dense and rather unspecific, could this be a reason?

Response:
We do not believe the signal to be particularly dense or unspecific for the following reasons. The antibodies used have been shown to be specific using various approaches (e.g. specificity was tested against competing peptides, immunolabeling on HEK cells expressing recombinant NMDAR etc.) (please see response to comment above). Furthermore, the values for the synaptic fraction of GluN1 are similar to those reported in the literature (Ladepeche et al., 2013, PNAS), and therefore seem within a normal range. In addition, the imaging parameters (e.g. threshold of the signal) were based on previous experience with this method and the respective antibodies, and were set to eliminate noise.

Comment: For the statistics of the mobility data it would be nice to show distributions averaged between different experiments to report the variability between experiments. One way could be to include the experimental variation in the cumulative representation?

We are not certain about the specific way this could be addressed. Below we attached a plot representing the mean squared displacement (MSD, i.e. surface explored by receptors) of NMDA receptors as function of time for WT and Fmr1KO conditions (+SEM). This plot shows that NMDA receptors explore larger areas over time in the KO condition, and illustrates the variability observed during our experiments. We hope that this addresses the reviewer's comment.

Response:

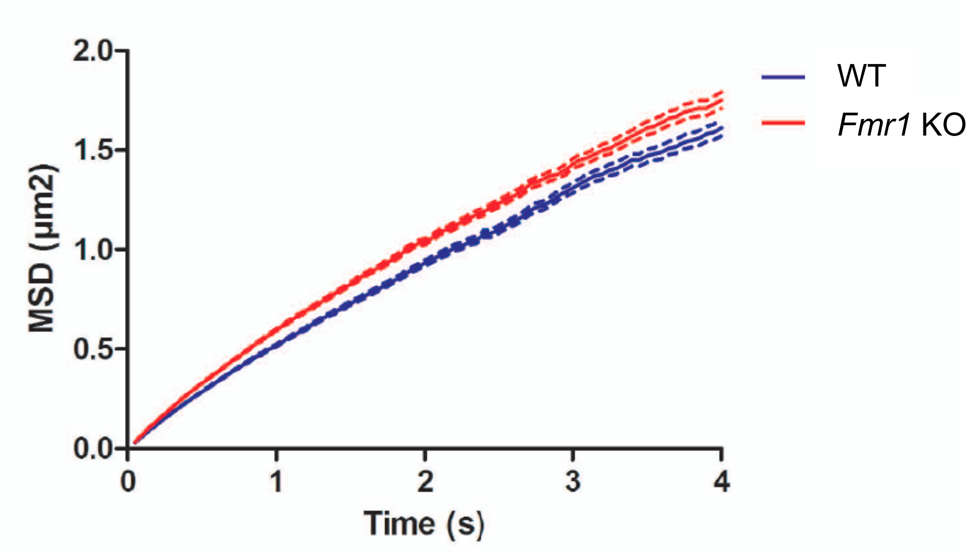

D. Appropriate use of statistics and treatment of uncertainties

Statistics reported for the diffusion coefficients seem particular for the reported difference of NMDA receptor diffusion in WT and *Fmr1* KO mice problematic. The large number of values (>10000 per group) might bias the strength of the used tests. I would suggest to rather compare the averaged distribution of WT and *Fmr1* KO group by comparing control experiments from different cultures to knock-out cultures.

Response:

We fully agree with the reviewer. Indeed, the large number of values in both WT and Fmr1 KO groups may bias the statistical significance of the results. To overcome this limitation, we randomly selected 2000 extra-synaptic and synaptic trajectories acquired from different WT and KO cultures as represented in the revised version of Figure 3b,c. As shown on the histograms and cumulative fractions, extra-synaptic and synaptic GluN1-NMDAR diffusion coefficients were still found to be significantly enhanced in the Fmr1 KO condition compared to WT, which confirms our initial observation.

Comment:

The cutoff for immobile receptors should be mentioned.

Response:

Mobile receptor: diffusion coefficient > 0.005 µm^2/s.

This information is already given in the Results section (page 4, paragraph "Exaggerated lateral diffusion of mGluR5 at synapses in Fmr1 KO neurons"(line 114)).

Comment:
For many experiments the number of experiments (cells) and their origin of how many different cultures is not given.

Response:
We have now included this information in each figure legend.

E. Conclusions: robustness, validity, reliability
Despite the mentioned problems above, the multitude of experiments, particular the use of interaction side specific peptides, seem to confirm the main conclusion that the interaction between mGluR5 and Homer1A is critical to the functional consequences reported here and by others.

Response:
We thank the reviewer for this evaluation of our study.

F. Suggested improvements: experiments, data for possible revision
As stated above, I would appreciate a revision of the data given on the dynamics of the involved receptors, particular in respect to previous work. In general the use of surface dynamics of key molecules as transmitter receptors can teach us a lot about molecular mechanisms behind and this work could be another nice example. However, large differences in the dynamics between experiments let wonder whether the method is robust enough to support the conclusions made in the paper.

Response:
As mentioned above, the diffusion coefficients measured in this study are indeed slightly different from previously reported values (see Sergé et al., 2002; Groc et al., 2004) which may be explained by several factors varying from one study to another, including i) the type of neuronal cultures used, ii) the developmental stages studied, iii) the animal species, iv) the acquisition frequencies, and v) the recording medium. Each of these factors may account for these differences between studies. Our data however match recent publications performed in a similar configuration (see Opazo et al., 2010; Dupuis et al., 2014), and all single-particle tracking experiments were conducted in the same recording configuration to allow unbiased comparison between the different experimental conditions. Most of all, when considering the apparent discrepancy in diffusion coefficients between our dataset and previous reports, one should keep in mind that absolute diffusion values should be handled with caution since they depend heavily on the methods used to perform single-particle tracking measurements, and thus vary from one study to another for a same subtype of receptor depending on the experimental configuration, although they fall in a similar range.

G. References: appropriate credit to previous work?
Previous work is appropriate cited

H. Clarity and context: lucidity of abstract/summary, appropriateness of abstract, introduction and conclusions

The paper is clear written and results discussed in the context.

Reviewer #2 (Remarks to the Author):

Summary statement
This manuscript addresses further contributions of the mGluR5 receptor in hippocampal synaptic and behavioral phenotypes of Fragile X mutant mice, through a Homer 1a mechanism.

Comment:
At the end of the Results section, page 7 line 229, mention is made of a "second generation" *Fmr1* mouse model. Differences in the mutation and breeding are briefly described later under Animals, page 10 lines 345-347. Apparently the mice used in the present experiments are different than the widely used *Fmr1* line of mice. A full description of the new line of mutant mice must appear at the beginning of the manuscript and in the abstract.
Rationale for the different type of mutation, how the mutation was generated, and the quality and quantity of genetic differences between the conventional *Fmr1* mutation, must be carefully explained. Comparisons of the full set of phenotypic differences between the present mouse and the conventional *Fmr1* line must be spelled out. The single reference 61 to a 2006 publication is insufficient.

Response:
We thank the reviewer for suggestions for improving the clarity of the manuscript. We have now added the following information:
"This model was generated by deletion of both the promoter and exon 1 of the Fmr1 gene, as described previously (Mientjes et al., *2006). These mice are distinct from the original Fmr1 KO (Fmr1^{tm1Cgr}) mouse line (Bakker et al., 1994), because they are deficient for both Fmr1 mRNA and FMRP protein.*
The rationale and strategy for the creation of this mutant have been described in the original paper describing this mutant (Mientjes et al., 2006), which was cited in our manuscript. Since this is not a "new model" and moreover, because the creation of this mutant has already been published (and this work was not performed by our groups) we are not certain that a full description of the creation of this model is appropriate in the context of our work. In the original Fmr1 KO model (Fmr1^{tm1Cgr}), a neomycin cassette was introduced in exon 5 of the Fmr1 gene. Although the protein product of this gene is not expressed (Mientjes et al., 2006), the promoter is intact and, due to an alternative splicing event, mRNA products containing Fmr1 sequences are produced (Yan et al., 2004;Mientjes et al., 2006, The consequence of these finding remain to be evaluated (Peier et al., 2005) and we would like to point out that numerous behavioral phenotypes are recapitulated between this and the "second generation" model, notably enhanced acoustic startle (Zhang et al., 2014) and object recognition memory and contextual fear memory (current manuscript). Furthermore, we have found that the novel phenotype described in the present paper i.e. that the lack or robust reduction of mGlu-LTD of NMDA currents is exhibited by both Fmr1 mutant mouse models. We would also like to insist that, far from being an orphan model, this mutant has been used in at least 16 publications of which we are aware, including 5 on which some of the current authors are also co-authors (please see list below – highlighted articles refer to those on which one of the senior authors is a co-author)

Neuhofer et al., 2015 Front Cell Neurosci. 2015 Mar 26;9:100. doi: 10.3389/fncel.2015.00100.
Martin HG et al., 2016 Cereb Cortex. 26(5):2084-92.
Tabet et al., 2016 Proc Natl Acad Sci U S A.113(26):E3619-28
Broek, JA et al., 2016 Mol Autism. 7:17. doi: 10.1186/s13229-016-0080-1
Wang et al., 2015 Front Cell Neurosci. 9:234. doi: 10.3389/fncel.2015.00234
Haberl, MG et al 2015 Sci Adv. 2015 Nov 20;1(10):e1500775. doi: 10.1126/sciadv.1500775.
Zhang, Y et al., 2014 Nat Neurosci. 2014 Dec;17(12):1701-9
Wijetunge LS et al, 2014. J Neurosci. 34(18):6405-12.
Mao, sc et al., 2013 PLoS One. 2013;8(3):e59580. doi: 10.1371/journal.pone.0059580.
El Fatimy, R et al., 2012 PLoS One. 7(6):e39338. doi: 10.1371/journal.pone.0039338
Jung, KM et al., 2012 Nat Commun. 2012;3:1080. doi: 10.1038/ncomms2045.
Vinueza Veloz MF, et al., 2012 Genes Brain Behav. 11(3):325-31.
Scotto-Lomassese, S J Neurosci. 2011 Feb 9;31(6):2205-15.
Levenga, J et al., 2009 Neurobiol Dis. 35(2):241-50.
Pilpel, Y et al., 2009 J Physiol. 2009 Feb 15;587(Pt 4):787-804.
de Vrij FM et al., 2008 Neurobiol Dis. 31(1):127-32.

Comment:
The novel object recognition task was conducted using non-standard methods. In fact, a spatial component has been added, by placing the second object in a different arm of an L-shaped maze, instead of presenting the first and second objects in adjacent locations within an open field.

Response:
As mentioned below by reviewer 2, there is significant body of evidence showing no, or very limited cognitive phenotypes in the mouse model of FXS. This issue has been discussed in a recent review (Gross, Hoffmann, Bassell and Berry-Kravis, 2015 Neurotherapeutics 2(3):584-608) which highlighted the importance of establishing robust phenotypic tests which are reproducible between labs. To this extent, we believe that the use of this non-classical configuration can be an advantage, because we have been able to reproduce a phenotype established previously in the Fmr1^{tm1Cgr} mutant in an FVB background in an independent lab (Busquets-Garcia et al., 2013, Nature Medicine 19: 603-607)

This test has been already described for evaluating the THC-induced memory impairment in the object recognition task (Puighermanal et al., 2009 Nat. Neurosci. 12, 1152–1158 and Puighermanal et al., 2013 Neuropsychopharmacology 38, 1334–1343) and in mouse models of Alzheimers disease (Ill-Raga et al., 2015 and Aso E. 2015, J Alzheimers Dis. 43(3):977-91). The non-classical configuration used here has a number of advantages over object exploration in a classical open-field: the maze is non-anxiogenic (the dark-coloured, non-reflective walls of the maze and narrow corridor, limit anxiety, which may be a confounding phenotype in certain open field configurations). In addition, the dim light conditions present in the L-maze may encourage self-directed exploration (e.g. as described in Mun et al., 2015 Scientific rep 5:17697). Moreover, since x,y exploration is limited by the limited arena size, the mice are encouraged to spend more time in exploring new features each time the arena is presented (namely objects) and object exploration time is improved (Aso et al., 2015). In addition, this configuration reduces the habituation sessions required for the classical open field configuration. The inventing laboratory have obtained a utility model to permit the commercialization of this kind of maze for the study of object recognition (#U200802592 Title: Device for testing cognition in animals. Inventors: A. Busquets-Garcia, R. Maldonado,

A. Ozaita)

To limit any confounds related to a spatial component (as raised above) we balanced the presentation of the novel object by randomly assigning it to the top or bottom arm of the maze. By scoring the exploration of objects on day 1 of the task (the acquisition phase of the task in which two identical objects were presented) we also verified that there was no bias with respect to one arm or another.

Comment:
This difference is important because *Fmr1* mice have been frequently reported to show hyperactivity. If the mutants move throughout the L-shaped arena at higher rates, they are likely to explore both arms more than their WT controls, and concomitantly are likely to spend less total time with the objects.

Response:
To address this question, we performed posthoc automated tracking using Ethovision software (Noldus) and analyzed the total distance moved, velocity and percentage of time active and inactive during exploration in the L-maze during habituation and on the training and test of object exploration. We found no differences between the genotypes for any of these parameters. These data are presented in Figures 13 - Supplementary Figure. We performed the same analysis for the AAV injected cohort and once again found no difference between the different groups. These data are presented in Figure 18 - Supplementary Figure. Based on this analysis and also the data related to object exploration on the first day (see below), we see no evidence supporting the potentially confounding effect of a hyperactivity phenotype.

Once again, we would also like to point out that the 'hyperactivity' phenotype is also somewhat inconsistently reported within the literature and may largely depend on light conditions, housing conditions, testing method, duration and genetic background. For example, Yan et al., 2004 performed analysis (open field in the dark phase and under red light illumination for 5 minutes per day over a 5 days period; $Fmr1^{tm1Cgr}$ model on FVB/C57Bl/6J hybrid background) and reported no increased activity. On the other hand, Bhattacharya et al., (2012) reported differences in the exploration of peripheral sectors of an open field (under 800 lux illumination, exploration during 15 mins during the light phase and using the $Fmr1^{tm1Cgr}$ model on the C57Bl/6J background). It is likely that the differences in locomotor activity in the latter case may be confounded by the strong light conditions, which may create an aversive or anxiogenic environment. Using actimetry, Pietropaolo et al., 2011 reported an increase in activity over 1 hr in the $Fmr1^{tm1Cgr}$ model on a FVB background only (no genotype effect was observed in the C57Bl/6 background). Spencer et al., 2005 reported increased distance travelled over a 30 minute exploratory period, using the $Fmr1^{tm1Cgr}$ model on a C57Bl/6J background, but precise lighting conditions were not reported, so it is difficult to evaluate the possible confounds of anxiety (which is also a phenotype of this model).

Comment:
Similarly, hyperactivity could artificially reduce freezing time in the contextual fear conditioning test.

Response:

We are aware of these possible confounds and for this reason analysed freezing during the first session (acquisition). We found no difference in freezing scores between wildtype and Fmr1 KO animals, suggesting that both groups learned from the experience. For the sake of brevity and because, as Reviewer 3 points out, the contextual fear memory phenotype is not novel, these data were not reported in the original manuscript (but are now reported here – Figure 15 - Supplementary Figure).

Nonetheless to respond more fully to this criticism (in the context of the previous comments), we also performed a more extensive analysis of activity during the first 2 minutes of exploration. To do this we initially tried to automatically track movement of the animal using Ethovision analysis software. However, due to the poor contrast between the animal and the conditioning chamber, in particular due to the rods of the floor, this approach was not successful. Therefore we manually scored exploration. Since the conditioning chamber is a richer environment than the open field arena due to the textured floor, non-uniform junction between walls and floor and also due to the olfactory and visual cues, exploratory behaviour is more complex and composed of walking, supported and non-supported rearing, nose-pokes, head-turns without full body movement and periods of grooming. We manually scored a subset of these behaviours: walking and rearing. We found no difference in these activity-based measures between wild-type and $Fmr1^{-/y}$ *animals (Figure 15 - Supplementary Figure).*

Comment:
The potential confound of higher locomotor activity can be easily evaluated. (a) Number of seconds spent exploring each object should be shown, in addition to the discrimination index, for the novel object recognition phase. (b) Number of seconds spent exploring each of the two identical objects during the familiarization phase should be shown. (c) An independent open field test for exploratory locomotion must be conducted, and presented with standard activity parameters.

Response:
a) and b) This is now shown (Figures 11, 12, 16 and 17 -Supplementary Figures)
c) We analysed open field exploration during a 30 minute period, under 40 lux illumination (measured in the centre of the arena) and analysed total distance moved and velocity (Figure 14 – Supplementary Figure). We found no genotype effect for these measures over the period analysed. To confirm these findings, we repeated the experiment using an independent group of age-matched animals (male, $Fmr1^{-/y}$ *and wildtype littermates of equivalent age) and this time recorded exploration over 60 mins. Once again, we found no significant difference in total distance moved and velocity, as well as periods of activity and inactivity (not shown).*

Comment:
There is no explanation about how the behavioral data shown in Figure 10 derives from a different experiment than the behavioral data shown in Figure 9 Panels d and e. Does Supplementary Figure 10 represent uninjected mice of the same genotypes as Figure 9? Or does Supplementary Figure 10 employ the conventional *Fmr1* knockout line of mice?

Response:
We apologise for the confusion with respect to the source of the data presented in these two figures. Since the object recognition memory and contextual fear memory phenotypes had not been previously demonstrated in the second generation Fmr1 KO model, we first performed pilot experiments using naïve (non-injected) mice of equivalent age to those used for the shRNA experiments. The data presented in the old Figure 10 (now Figures 11 and 15-

Supplementary Figures) pertained to data from these pilot experiments. Having confirmed this phenotype, we repeated the experiments using a separate cohort of animals, stereotaxically injected at P21 and tested at 12 weeks of age. This data is now presented in Figure 10 (previously Figure 9). We have now amended the figure legends and in addition added the following to the main text to further clarify this point:
"Since these phenotypes have not previously been investigated in the second generation *Fmr1*KO mouse model, we initially performed a pilot experiment to determine whether we could recapitulate these phenotypes." *Lines 231-233, page 7*

Comment:
Full statistical results must be added (e.g. F value, degrees of freedom, stringent posthoc analyses). Stating only the p value for ANOVA and t-tests is insufficient.

Response:
We have now included further information regarding the statistic in each figure legend.

Comment:
The authors ignore the very large body of literature showing normal cognitive scores in *Fmr1* mice on many learning and memory tasks conducted by many independent labs, including the novel object recognition and fear conditioning assays which were conducted in the present studies. The authors are encouraged to read the behavioral literature more fully.

Response:
We thank the reviewer for their suggestions for improving our manuscript. We are aware of the rich body of literature related to the behavioural characterization of the Fmr1KO mouse. Unfortunately, due to the limited number of references permitted by the journal (70) and the diverse approaches presented in the manuscript, we had to reduce our discussion of the relevant literature to a minimum.

With respect to the issues raised about the normal cognitive scores in Fmr1KO mice, we are aware of these conflicting results, as well as the suggestion that genetic background may play a role in the manifestation of certain phenotypes (e.g. Pietropaolo et al., 2011; Spencer et al. 2011). As pointed out above, one of the current and future challenges for research in this domain is to identify phenotypes, or phenotyping approaches, that are reproducible between laboratories. To this extent, we find it to be a strength of our findings that we were able reproduce phenotypes using methods established by independent laboratories using the $Fmr1^{tm1Cgr}$ mouse in an FVB background (Busquets-Garcia et al., 2013; Gomis-González et al., 2016; Oddi et al., 2015).

With respect to the contextual fear memory data published previously, we would like to point out that the methods used here differ from those of Paradee et al., (1999), Dobkin et al., (2000), van Dam et al., (2000), Peier et al., (2005), in the respect that no auditory cues were presented. In addition, the conditioning described in our manuscript occurred over a single session, as described previously (Frankland, Bontempi et al., 2004) and not over successive days as described by Eadie et al., 2012. In addition, although inconsistent results were reported in these earlier studies, more recent work, such as Eadie et al., 2012 (contextual memory following repeated exposure to conditioning chamber); Oddi et a.l, 2015; Lim et al., 2014 (trace fear conditioning) and Ding et al., 2014 reported phenotypic differences.

With respect to novel object recognition, we would like to point out that this paradigm has recently been highlighted as one of the more promising behavioural tools for investigating cognitive defects in the Fmr1KO mouse (Gross et al., 2015) e.g Ventura et al., 2004; Seese et al, 2014; *Bhattacharya et al.,* 2012. *In particular, the presentation of only two objects and the testing of long-term memory (24h) present major refinements over previous work. As mentioned above, the reproducibility of the approach employed in our manuscript and its utility for examining pharmacological correction is also an advantage.*

Finally, we would like to make the point that a full behavioural characterization of the second-generation Fmr1 KO model was not the major focus of this manuscript. Given the constraints of the journal (limit 70 references) we hope that the Reviewer will accept our apologies for not being able to discuss these points in greater detail, with reference to the appropriate literature, in the current manuscript.

Comment

Absence of the full cognitive datasets, and of the necessary open field control experiment, represent serious flaws in this manuscript.

Response:

We appreciate the suggestions from the Reviewer to improve the presentation and interpretation of our work. We have now provided analysis for all the questions presented above. We hope that the Reviewer will understand that a more complete behavioural characterization is beyond the scope of the current work and will be addressed in a later article.

Reviewer #3 (Remarks to the Author):

Summary:

This manuscript details interesting experiments investigating the interaction of mGluR5 and NMDA receptors in a mutant mouse model of fragile X (the *Fmr1* KO). Specifically, the authors explore the dynamics of receptor diffusion in cultured neurons (using Q-dots), finding that the mobility of mGluR5 and the NMDAR are increased in the *Fmr1* KO neurons. No change is seen in the mobility of GluA2, suggesting that the effect is somewhat specific to the mGluR-NMDAR complex. Based on the previous results of the Huber lab, they then show that disrupting mGluR5-Homer binding mimics and occludes the increased receptor trafficking in the *Fmr1* KO. Additionally, the co-localization of mGluR5, NMDAR and Homer1 is increased in the synapses of the *Fmr1* KO neurons.

In addition to the in vitro work, the authors examine whether the NMDAR component of LTD is altered in young (P11-15) hippocampal slices from *Fmr1* KO mice. Specifically, they measure NMDAR EPSCs during stimulation of group 1 mGluRs using the agonist DHPG. They find that the NMDAR component of DHPG-LTD is deficient in the *Fmr1* KO, and this is mimicked by disruption of the mGluR-Homer interaction. To show that enhancing the mGluR-Homer interaction could rescue the deficit in NMDAR function in the *Fmr1* KO, they injected shRNA to the dominant negative Homer 1a (or scrambled shRNA), and measured the NMDAR component of DHPG-LTD in the *Fmr1* KO. They also performed novel object recognition and contextual fear conditioning on injected animals, and show a remarkable rescue of both behavioral deficits.

Overall, this study is an interesting addition to the literature regarding the regulation of mGluR-NMDAR interaction, and the role of this in the fragile X mouse model. The in vitro Q-dot experiments are particularly compelling. There are, however, a number of drawbacks that should be addressed, particularly with respect to the electrophysiology and behaviour experiments.

Response:
We thank the reviewer for this overall positive evaluation of our work and will address the specific comments in detail.

Comment:
1.) The in vitro experiments using Q-dots show a convincing increase in the mobility of mGluR5 and NMDARs in the *Fmr1* KO neurons. However, these experiments use Mitotracker labelling to define "synaptic sites" without providing verification using a more conventional synaptic marker (i.e., synaptophysin, PSD-95). This is important because all of the work shown in Figures 5-7 is dependent upon the "synaptic" association.

Response:
The use of mitotracker as a synaptic marker has been validated previously Groc et al., 2004 by examining co-localisation with the synaptic marker synaptotagmin. This approach has been extensively used for localizing synaptic sites during single particle imaging by the teams of Laurent Groc (Groc et al., 2007) and Daniel Choquet (Opazo et al., 2010). In the context of fragile X syndrome, it is preferable to use this approach instead of transfection, because transfection with a synaptic marker such as homer or PSD-95 may precociously alter synapse development. Since we wanted to examine native receptors this was an important factor to avoid. In addition, a number of mRNAs encoding synaptic markers are targets of FMRP (Darnell et al., 2011) and their expression may thus be altered in Fmr1KO cultures further element of complexity to an already difficult mechanism to be disentangled. Finally mitotracker labeling has the advantage of being rapid (1 min) and this is important in the context of live imaging where neuronal health is crucial.

Comment:
These data would be much more robust if there was validation using another method, and if the total co-localization of mGluR5 and GluN1 was shown (not just the "synaptic" fraction).

Response:
We used QD and triple immunocytochemistry for demonstrating an increased permanence of mGluR5 and NR1 at synapses and an increased co-localization of mGluR5 and NR1, respectively. The analysis of data from both experiments leads to the same conclusion. The small yet statistically significant increased level of mGluR5/NMDAR1 co-clustering at synaptic sites, makes very unlikely the detection of this with other methods such as immunoprecipitation. The increased functional interaction between, GluR5 and NR1 could be studied by BRET. This method is not used in any of the laboratories involved in the present study, and would require transfection, perturbing the system and making even more difficult to compare WT and KO. In the present study we decided to study naïve receptors only.

Comment:

2.) The LTD experiments are interesting in light of the specific focus on NMDAR EPSCs. However, there are a few issues. First, the developmental time-point at which these experiments are performed is P11-15, at which point mGluR-LTD is presynaptic (Nosyreva and Huber, 2005). The authors, however, interpret their effects in terms of a postsynaptic mechanism. This should be clarified by including measurements of PPR. These experiments should also be accompanied by standard LTD measurements to ensure that they are indeed inducing DHPG-LTD with their protocol.

Response:
We totally agree that mGluR-LTD has a presynaptic component at the developmental age that we studied (P11-15): this was shown by Nosyreva and Huber (2005) and confirmed by other research groups including ours (Costa et al., 2012). In our previous study, we have already measured PPR in the same synapse in wt and Fmr1 KO mice at P11-15 in the same experimental conditions as those used in the present work: we confirmed that activation of mGluRs by DHPG indeed modified the PPR. At the same time, we show that DHPG application also induced internalization of AMPA receptors at P11-15, similar to data obtained at later developmental stages. Thus activation of mGluRs in the CA3-CA1 synapse at P11-13 induces both pre- and postsynaptic effects, which do not mutually exclude each other and both contribute to mGluR-LTD of AMPAR-mediated synaptic currents.
In the present work, we studied mGluR-LTD of NMDAR-mediated synaptic currents: we show for the first time that this form of plasticity was substantially reduced or even absent in Fmr1 KO mice and this condition was mimicked by disrupting the interaction between mGluRs and Homer PSD scaffolding proteins. Consistently, we also show that mGluR-LTD of NMDAR-mediated EPSCs was rescued by reducing Homer1a expression, which restored the interaction between mGluRs and long Homer proteins. The post-synaptic location of the observed effects is also inferred by the almost exclusively post-synaptic localization of Homer proteins, as shown previously. Thus, although we have previously confirmed that DHPG exerts presynaptic effects, our present data indicate that mGluR-LTD of NMDAR-mediated synaptic currents relies on a post-synaptic mechanism. Standard LTD measurements have been performed in our previous work (Costa et al., 2012; Costa et al. 2015) in the same experimental conditions, showing that we are indeed inducing DHPG-LTD with our protocol.

Comment:
3.) In Figure 7, both the mGluR5ct and control peptides seem to have an effect on the *Fmr1* KO. This should be discussed.
Accordingly, the electrophysiology shown in Figure 8 should include a peptide-treated *Fmr1* KO group.

Response:
We carried out new experiments showing that Ct-peptide do not have any effect on Fmr1 KO in electrophysiology experiments aimed at eliciting the mGlu-LTD of NMDA currents. This experiments were carried out on KO2 in a C57BL6 background, and for this reason were not added to figure 8, but presented as a new supplementary figure. These results corroborate the finding that mGlu-LTD of NMDA currents are reduced in KO mice.
Regarding the effect observed in the immunocytochemistry experiments shown in Figure 7, although both mGluR5ct and control peptides seemed to have an effect, as this reviewer points out, this was not statistically significant. Moreover, the effect observed, which can be due to an unspecific perturbation of the membrane, is in the opposite direction as expected which make this effect not relevant for our study and not in contrast with our main conclusion.

4.) The in vivo injection experiments require a number of controls, including an estimation of the efficiency of the Homer1a knockdown

Response:
The shRNA and control shRNA constructs were validated previously in the CNS (in vitro and in vivo) Tappe et al., 2006. Nature Medicine 12, 677 – 681.

In addition to this validation, we have now provided data showing an estimation of knockdown efficiency in hippocampal neurons. In cultured hippocampal neurons, homer1a mRNA levels are reduced by 65-68% (depending on the choice of normalizing gene) with respect to expression levels present in AAV-scr infected (control) neurons (Figure 21 - Supplementary Figure b). Following stereotaxic delivery, an estimated 97% of neurons within the stratum pyramidale of CA1 were transduced with our AAV vectors expressing shRNA and control constructs (estimated by co-expression of the reporter gene, eGFP) (Supplementary Figure 21c).

Comment:
5.) The behaviour experiments seem to be the weaker parts of this study. The descriptions of the work are not very thorough, and the data appear hastily put together. For example, the methods state that behaviour experiments were only performed in the *Fmr1* KO, yet there is data shown for WT. Were these experiments performed blind to genotype? Were the groups age-matched and counterbalanced for treatment?
These details are essential for assessing the results, especially since the effects shown are so uncharacteristically large.

Response:
We apologise for these omissions in the presentation of this data. We have now included a fuller description of the methods used as well as a substantial number of controls as requested by reviewer 2. All behavioral experiments were performed using age-matched cohorts of WT and Fmr1-/y littermates. Treatments were balanced within litters and with respect to genotype and semi-randomized with respect to the assignment of treatments to individual animals. Contextual fear conditioning and object memory experiments were performed and analyzed blind to genotype and treatment. We have now included changes to the methods section to reflect this information. Please see: "Methods", "Behavioral Testing" section, "Novel object recognition task" and "Contextual fear conditioning task" subsections, page 17.

In addition, we would like to note that the mice were injected at post natal age 21 days and the behavioral training started 9 weeks after virus infusion. To this extent it is important to note that Homer1a levels are significantly upregulated in the third week of postnatal life and peak between 3-5 weeks of age. Moreover, it is important to note that homer1a is upregulated in response to activity and that circuit level activity is perturbed in fragile X syndrome, likely leading to an overall exacerbation of homer1a signaling. Since the refinement of neuronal circuits is critically regulated by circuit level activity, we believe that early and prolonged intervention during a critical period could explain why these effects are so pronounced.

Comment:
Moreover, many of these findings have been previously published. The deficits in contextual fear conditioning have been described previously (Eadie et al. 2012, Auerbach et al. 2011).

While it is true that reduction of Homer1a in the *Fmr1* KO has not been found to rescue these specific phenotypes, it has been thoroughly studied with respect to other phenotypes (Huber). Additionally, the deficit in contextual fear conditioning has been attributed to a hypofunction of NMDARs in the dentate gyrus (Eadie et al. 2012). How does this fit with the authors' other findings, which are focused on CA1? New finding?

Response:
We did not intend to claim that the behavioral phenotypes presented here are novel. The description of these phenotypes in naïve (non-injected) animals was included to demonstrate that we could recapitulate these phenotypes in the second-generation mouse model (as we have done previously in Zhang et al., 2014), in order to validate the phenotype prior to demonstrating its correction. Additionally, although the very elegant work of Kimberly Huber's team has indeed demonstrated rescue of anxiety and seizure susceptibility, we find it important to note that the primary feature of Fragile X syndrome is ***intellectual disability****. Instead, as noted in a recent review (Gross, Hoffmann, Bassell and Berry-Kravis, 2015, Neurotherapeutics) the majority of behavioral characterizations of the Fmr1 KO mouse have focused on autism-associated features, including anxiety. However, as FXS is an intellectual disability disorder, the development of therapeutic strategies for the improvement of cognitive defects in FXS is also a priority. In this sense, we feel that our findings showing the correction of two hippocampal-dependent cognitive phenotypes is extremely pertinent to for the development of novel therapeutic strategies.*

However, most importantly, our work describes two extremely novel findings: the higher surface mobility of mGluR5 and the altered plasticity of NMDA receptors, as a consequence of mGluR5/Homer disruption. The functional interaction between mGlu5 and NMDA and their reciprocal modulation is well established, but never investigated in the context of FXS. We also provide a strong correlation between these findings and the correction of the aforementioned behavioural phenotypes.

Whilst it is indeed true that hypofunction of NMDAR in the dentate gyrus has been associated with a defect in contextual fear conditioning, these findings are not mutually exclusive with our own work. The NMDA/AMPA ratio has not been studied before in the CA1 region using our protocols. Indeed, hypo-functionality of NMDA receptors have been detected in other regions of FXS such as anterior pyriform cortex (Gocel and Larson, 2012), prefrontal cortex (Martin et al., 2016) and nucleus accumbens of striatum (Neuhofer et al., 2015). Indeed, taken together, they lend support to the idea that dysfunction of NMDAR and mGluR5 can be associated in FXS and both receptors and their interaction might be considered as a new avenue for FXS therapy.

Reviewers' comments:

Reviewer #1 (Remarks to the Author):

The authors tried to answer my methodological questions mostly in bringing arguments that experiments have been done in the past in slightly different systems and experimental configurations and recent publications of the group show comparable receptor mobility in neurons. I have to say I disagree with both arguments.
First, for the reference given by the authors the mobility of NMDAR in Dupuis et al. 2014 is reported to be under control conditions around 0.03-0.04 $\mu m^2/s$ (Fig.1), whereas in the current manuscript even in synapses NMDAR under control conditions are much more mobile ($D > 0.1 \mu m^2/s$).
Second, the argument that within mixed cultures, receptors are even faster than in glia-free Banker type cultures sound counterintuitive. There I would expect to see the opposite, since particular synapses are probably enwrapped by glia endfeets and might rather impose constrain of the QD-labeled receptors that like to enter or leave the synaptic membrane.
Third, the authors claim that diffusion might be influenced by the age of the culture. Surprisingly, the culture of previous studies were between 3-11 DIV, whereas here the cultured cells where 12-15 DIV. If at all, I would expect to see lower diffusion coefficients since the surface dynamics of proteins decrease with the maturation of neuronal networks (Borgdorff, Choquet 2003, Groc et al. 2006).
Forth, the authors seem to be aware that absolute values of diffusion coefficients are highly vulnerable but do claim that the effects they report are specific for the effects they describe.
I would take this point never as an argument to defend my data, rather I would expect a more careful analysis and design of the experiments to circumvent such high variability.

Here i would like to see an improvement on the data rather than bringing arguments that do not hold.

Reviewer #2 (Remarks to the Author):

The behavioral data presentation is now more complete. The authors have successfully addressed the potential hyperactivity confound with additional experiments. Relevant new data appear in Supplementary Figures 13-15.

Statistical presentation remains insufficient. What is needed are actual ANOVA F values, degrees of freedom, and p values for each factor, and for numerical values obtained from each posthoc comparison. Instead, what appears in the figure legends are means + standard errors, which are unnecessary since already shown in the data, and the names of the statistical tests used, which should have already been summarized in the Statistics paragraph of the Methods section, lines 648-657.

In the Response to Reviewer 2, the authors clearly explain the Fmr1 mutant model which

they used in the present study. However, this information still does not appear early within the manuscript. It is essential that the text states and describes this line of mice, to differentiate it from the more standard Fmr1 knockout that readers will immediately assume were used. Explicit naming and/or description of this specific Fmr1 mutation must appear briefly in the Abstract and Introduction, with a reminder to the readers in the Discussion section, rather than only in the Methods section which readers may not read carefully.

Several of the references to the original generation and phenotyping of this Fmr1 mutant model, which appear in the Response list, should appear in the manuscript reference list, and be cited within sentences describing this Fmr1 line. Either the authors could request that the journal editor allow them to exceed the 70 reference limit, or the necessary additional references can be placed into Supplementary Materials.

In the Response to Reviewer 2, the authors explain that their modifications of the novel object recognition task were deliberate, to enhance detection of a cognitive phenotype, and based on previous publications using these methods. However, again, the reader does not receive sufficient alerts that this is not the standard novel object recognition assay. Clear statements must be added to the Abstract, Introduction, Methods, Figure legends, Results and Discussion sections, emphasizing that these were modifications of the standard novel object recognition task, in which complexity was introduced by adding a spatial compartmentalization feature. Further, the authors are strongly encouraged to use a different name for their novelty test, which better describes their method, and which clearly distinguishes their method from standard novel object recognition.

Supplementary Figure 22 is stated as proving that no object bias existed for exploring the lego versus the bottle cap. However, the p values obtained in this simple t-test is $p=.06$, which is very close to statistical significance. Ns are only 4 per object. The authors are encouraged to expand this essential control experiment with larger Ns. If an innate preference for one object emerges, the authors are encouraged to choose different objects.

Differences between how the authors conducted fear conditioning, and how fear conditioning was conducted by the many other labs that did not detect fear conditioning deficits in Fmr1 knockout mice, must be clearly stated within the Discussion section.

Because the literature on lack of cognitive deficits in Fmr1 mice is a major source of controversy in the field, greatly increased emphasis on how the present behavioral assays differ from most of the standard literature on Fmr1 mice is essential.

At present there is almost no description of the behavioral results in the Discussion section. The single sentence in lines 329-330 is entirely insufficient. If the authors want to state conclusions about cognitive deficits and rescues, relevant to the primary diagnostic symptom of Fragile X syndrome, considerably more text must be devoted to interpreting their behavioral findings appropriately.

If the authors are not as interested in the behavioral component, as indicated by a statement in the Response that behavioral characterization was not a major focus of the

manuscript, then the behavioral figures should be removed entirely.

Reviewer #3 (Remarks to the Author):

The authors have carefully considered the reviewers' comments, and the manuscript has been strengthened significantly. This is particularly true of the new behavior data and more thorough explanation. They should be commended for a well revised manuscript that will make an interesting addition to the literature.

Reviewers' comments:

Reviewer #4 (Remarks to the Author):

Their reported values for the diffusion coefficient of GluN1 are indeed a little higher than reported in comparable studies, but the authors provide plausible arguments for the difference in the measured diffusion coefficient. The precision and reproducibility of these experiments depends greatly on the experimental conditions (type, age, species of culture); detection (antibody-labeled endogenous receptors, or exogenously tagged); methods used to track receptor (QDs or beads); acquisition parameters (frame rate etc.) and can be highly variable. But in fact, as they show in the appendix in the second rebuttal, the numbers are not terribly different. The skewed, non-Gaussian distributions in diffusion coefficients make that the median and mean values report very different aspects of the population, and I believe this might have caused some of the confusion. What is most important, is that the experimental conditions within experiments are the same. Based on the presented information and data, the authors convincingly demonstrate that the lateral diffusion of mGluR5 and NR1 is altered in Fmr1 KO cultures, and that in particular mGluR5 diffusion is increased in "synaptic areas".

I do want to comment on the use of MitoTracker as a marker for synapses (as was noted by reviewer 2). Although MitoTracker has "extensively been used", at best it is a very rough estimate of synaptic sites; 60-70% overlap with synaptotagmin according to Groc et al., 2004, meaning that in 30-40% of the cases, synaptic tracks are not correctly assigned. This most likely reflects the fact that the majority of spines does not contain mitochondria (see e.g. Li et al., Cell 2004). Moreover, the morphology of mitochondria is highly variable and does not relate at all to synapse shape. And, important for this study, mobility, location, shape and number of mitochondria are all influenced by neuronal activity. So, even if MitoTracker was the best possible approach for estimating synaptic location in these experiments, the authors should at least demonstrate that the ability of MitoTracker to label synaptic sites is similar between wild-type and Fmr1 KO cultures, in which the activity of synapses is severely altered.

We thank the Reviewers for their consideration of our manuscript and for their suggestions for improvement. Below, we present a point-by-point response to their comments. Modifications to the manuscript are indicated by highlighting in yellow in the main text, and where appropriate, below.

Reviewer #1 (Remarks to the Author):

Comment:
The authors tried to answer my methodological questions mostly in bringing arguments that experiments have been done in the past in slightly different systems and experimental configurations and recent publications of the group show comparable receptor mobility in neurons. I have to say I disagree with both arguments. First, for the reference given by the authors the mobility of NMDAR in Dupuis et al. 2014 is reported to be under control conditions around 0.03-0.04 µm²/s (Fig.1), whereas in the current manuscript even in synapses NMDAR under control conditions are much more mobile (D > 0.1 µm²/s).

Response:
We apologize for not replying more fully to this question in the first revision. To address this concern, we now provide a supplementary table (Supplementary Table) in which we compare our mobility values for NMDA receptors with those obtained in complementary studies performed under similar conditions (e.g. Dupuis et al., 2014). To permit a more stringent comparison between these values we adopted the following criteria. We only included studies that:
*i) measured **native** NMDA receptors **within** the synaptic compartment and*
(ii) which include both the mobile and immobile fraction of receptors.
iii) present the median mobility values
We added Dupuis et al., 2014 to this list because even though mean values were presented in this publication, the median value is available (0.03113 ± 0.008-0.0574 µm2/s; J. Dupuis personal communication)
*To permit a comparison with our own values, we then calculated the median value for receptor diffusion (**0.071 µm2/s**; IQR = 0.026-0.144 µm2/s; the mean value was reported in the manuscript). The range of values presented in this comparison was 0.021-0.32* µm^2/s. *The median value obtained from our work was thus similar to most of the studies mentioned in this table (with the exception of Bard et al., 2010, for which higher values (0.11 and 0.32 µm2/s) were obtained). This comparison should convincingly demonstrate that our value is very similar to those obtained by these other studies despite any differences in the type- (Banker/mixed) or age (DIV 9-20) of culture, or the species (rat/mouse).*

*With respect to mGluR, Reviewer #1 has previously suggested that the diffusion values for mGluR5 receptors are 'extremely' high when compared to the study by Sergé et al., 2002, J Neuroscience. The latter study reported mGluR receptor pools with high and low diffusion, and the respective diffusion values were both **higher** and **lower**, respectively, when compared to our study. Thus, we are not certain which of the values Reviewer #1 refers to. Importantly, we are not sure to what extent this comparison is justified given that crucial technical aspects of the two studies are completely different. For example in the Sergé et al study, the authors used latex beads to label individual mGluR receptors. These beads are ~500 nm in diameter and thus roughly 20 times larger than QDs! Consequently, whether or not diffusion values are comparable under those different conditions is somewhat debatable. On the contrary, a study by Renner et al. 2010 (Neuron) using QD labeling of native mGluR5 in rat hippocampal neurons (21-27 days) reports a lateral diffusion of synaptic mGluR5 of 0.0146 µm^2/s (median) — a value that is very*

comparable to ours (0.017 ± 0.001 μm^2/s, mean ± s.e.m.).

Finally, following his or her initial evaluation of our manuscript, the Reviewer asked us to discuss the discrepancy between our results and those reported previously in the literature. Given the aforementioned discussion, we hope that we have now established that this difference is negligible. Nonetheless, we have added the following sentence to the Discussion (page 8, lines 283-285) in which we also cite Renner et al., which was not cited previously :

> "Noteworthy, although these mobility values may be influenced by a number of experimental variables, our measures for WT neurons are consistent with those reported under similar conditions (e.g. 24)"

In summary, we hope to have provided convincing arguments, well supported by the existing literature, that our mobility values are in the observed range of reported values, therefore corroborating the validity of our conclusions.

Comment:
Second, the argument that within mixed cultures, receptors are even faster than in glia-free Banker type cultures sound counterintuitive. There I would expect to see the opposite, since particular synapses are probably enwrapped by glia endfeets and might rather impose constrain of the QD-labeled receptors that like to enter or leave the synaptic membrane.

Response:
We agree that these assumptions would appear to be intuitive, however, as an analysis of the literature (please see Supplementary Table) reveals, both lower and higher values have been obtained with Banker cultures compared to mixed cultures. Furthermore, it remains to be shown whether glial processes reach into the synaptic cleft in mixed cultures, and whether this would indeed influence the diffusion of receptors. Finally, we would like to reiterate that our values fall within the range of values reported in the literature (irrespective of culture type).

Comment:
Third, the authors claim that diffusion might be influenced by the age of the culture. Surprisingly, the culture of previous studies were between 3-11 DIV, whereas here the cultured cells where 12-15 DIV. If at all, I would expect to see lower diffusion coefficients since the surface dynamics of proteins decrease with the maturation of neuronal networks (Borgdorff, Choquet 2003, Groc et al. 2006).

Response:
We believe that our responses may have been misconstrued and we apologize to not being clearer in our explanation. Here, we simply pointed out that there is a range of parameters that might affect the diffusion values obtained. In particular, we cited this as one explanation for why our values were not the same as Sergé et al., J Neuroscience, 2002, in which the cultures used were very different in age (3-8 DIV) compared to our study.

However, in addition to the age, it is important to note that the experimental conditions and analysis criteria employed in these two studies are completely different. First, in Sergé et al., 2002 a transfected myc-tagged or HA-tagged receptor construct was used and the tagged receptor was expressed under the control of a hybrid (non-native) promoter. In our case native (endogenously expressed) receptors were assayed. It is possible that the tagged protein does not

have the same dynamics within the membrane. It is also possible that over-expression of the receptor from a plasmid-based construct, or indeed the transfection itself alters the physiology of the neurons, their state of maturity or leads to a greater accumulation of receptor protein in the membrane, so it is not entirely equivalent to the normal developmental trajectory of a neuron. However, as mentioned above, the most critical difference between this early work and our own is that ***0.5 µm beads conjugated to the myc antibody were used instead of QD-based detection*** *— these beads are ~20 times larger than QDs! In addition, they did not distinguish between synaptic and extra-synaptic mobility (see also our point above). All of these factors may lead to the absolute diffusion values being different between their study and our study and for this reason we felt that a direct comparison of these values is not valid. As mentioned above, there is a more recent study (Renner et al., 2010) reporting diffusion values for mGluR5 that are comparable to ours and for which the experimental conditions are similar.*

Comment:
Forth, the authors seem to be aware that absolute values of diffusion coefficients are highly vulnerable but do claim that the effects they report are specific for the effects they describe.
I would take this point never as an argument to defend my data, rather I would expect a more careful analysis and design of the experiments to circumvent such high variability.

Response:
We believe that this is a misunderstanding. We think that there might be a confusion between the variability of values reported in the literature, which we clearly acknowledge despite very similar values obtained in the various studies (see above), and our inter-experiment variability, which was carefully tested and controlled for.

As demonstrated in the Supplementary Table there is some variability in the absolute values reported in the literature. This variability may reflect differences in the experimental conditions, species, type of culture etc). Thus it is important to always compare experiments performed under the same conditions i.e. within a study (as we have done). In the previous response we stated that "absolute values should be handled with caution" meaning that one should not place too much emphasis on whether our wild type values were exactly the same as those reported in the literature because many factors might cause slight difference between labs. We apologize for not being clear enough in our intent.
With respect to vulnerability, we would like to insist on the fact that this is not the case. Indeed, we were meticulous in ensuring that our experimental conditions were the same throughout (including maintaining a strict breeding strategy so that cultures were produced from WT and KO embryos at the same time and that the dissection was always performed at the same time of day by the same person). The antibody incubation and imaging parameters were also strictly controlled. Therefore the variability between experiments in our condition was minimal.

Reviewer #2 (Remarks to the Author):

Comment:
The behavioral data presentation is now more complete. The authors have successfully addressed the potential hyperactivity confound with additional experiments. Relevant new data appear in Supplementary Figures 13-15.

Response:
We thank the reviewer for this statement.

Comment:
Statistical presentation remains insufficient. What is needed are actual ANOVA F values, degrees of freedom, and p values for each factor, and for numerical values obtained from each posthoc comparison. Instead, what appears in the figure legends are means + standard errors, which are unnecessary since already shown in the data, and the names of the statistical tests used, which should have already been summarized in the Statistics paragraph of the Methods section, lines 648-657.

Response:
We decided to give p-values and the types of statistical tests for each data in the figure legends (rather than the statistics section), since they vary depending on the type of experiment performed. We now provide these additional statistical values (F-values etc.) in the corresponding figure legends

Comments:
In the Response to Reviewer 2, the authors clearly explain the Fmr1 mutant model which they used in the present study. However, this information still does not appear early within the manuscript. It is essential that the text states and describes this line of mice, to differentiate it from the more standard Fmr1 knockout that readers will immediately assume were used. Explicit naming and/or description of this specific Fmr1 mutation must appear briefly in the Abstract and Introduction, with a reminder to the readers in the Discussion section, rather than only in the Methods section which readers may not read carefully.

Response:
We have now integrated this information throughout the manuscript as suggested by the Reviewer. Specifically, the following mentions are made:

Abstract (page 2, lines 35-37):
Here, we probed the consequences of mGluR5/Homer scaffold disruption for mGluR5 cell-surface mobility, synaptic N-methyl-D-aspartate receptor (NMDAR) function, and behavioral phenotypes in the second-generation *Fmr1* knockout (KO) mouse.

Introduction (page 3, lines 92-95):
> "The majority of these experiments were performed using the second-generation *Fmr1* KO mouse line, which lacks both *Fmr1* mRNA and FMRP[33] Certain electrophysiological experiments were performed both in the second- and first-generation[34] mutants, demonstrating good comparability between these models."

Results (page 4, lines 110-112):
> "Here we used a single nanoparticle (quantum dot; QD) imaging approach to track surface mGluR5 in live hippocampal neurons derived from second-generation *Fmr1* KO and WT mouse embryos (12-15 days *in vitro*)".

Results (page 7, lines 238-242):
> "Since these phenotypes have not previously been investigated in the second generation *Fmr1*KO mouse model, we initially performed a pilot experiment with a separate batch of experimentally naïve animals to determine whether we could recapitulate these phenotypes. Consistent with the aforementioned studies employing the first-generation *Fmr1* KO mouse line, the second-generation model, used here, exhibited similar decreases in the discrimination index (DI) in the NOR task… and in the percentage of freezing compared

with WT mice following retrieval of CFC memory …"

Discussion (page 10, lines 347-349):

Our study using the second generation model replicates findings reported for the first generation *Fmr1*^{tm1Cgr} model, suggesting that these tests are robust and reproducible between independent laboratories.

Methods (page 11, lines 399-404)

Second generation *Fmr1* KO mice[33] were mostly used in our study. This model was generated by deletion of both the promoter and exon 1 of the *Fmr1* gene, as described previously[33]. These mice are distinct from the original *Fmr1* KO (*Fmr1*^{tm1Cgr}*)* mouse line[34], because they are deficient for both *Fmr1* mRNA and FMRP protein. This mouse model has largely been used for physiological, molecular, and anatomical studies (e.g. 60, 76-81), however limited behavioral characterization has also been performed (e.g. 60, 82-85).

Methods (page 16, ljnes 615-616)

"For the electrophysiological recordings of NMDAR currents from *Fmr1* KO mice (second generation) …"

Comments:
Several of the references to the original generation and phenotyping of this Fmr1 mutant model, which appear in the Response list, should appear in the manuscript reference list, and be cited within sentences describing this Fmr1 line. Either the authors could request that the journal editor allow them to exceed the 70 reference limit, or the necessary additional references can be placed into Supplementary Materials.

Response:
Again, as stated above, we now added several references of studies that used this mouse model, and also additional information regarding this mouse model (see results and methods e.g. Methods, page 11 lines 403-404).

Comments:
In the Response to Reviewer 2, the authors explain that their modifications of the novel object recognition task were deliberate, to enhance detection of a cognitive phenotype, and based on previous publications using these methods. However, again, the reader does not receive sufficient alerts that this is not the standard novel object recognition assay. Clear statements must be added to the Abstract, Introduction, Methods, Figure legends, Results and Discussion sections, emphasizing that these were modifications of the standard novel object recognition task, in which complexity was introduced by adding a spatial compartmentalization feature. Further, the authors are strongly encouraged to use a different name for their novelty test, which better describes their method, and which clearly distinguishes their method from standard novel object recognition.

Response:
*We have renamed the task the "*One-trial-acquisition novel object recognition episodic memory" task

We have now modified the manuscript in the following manner:
Results (page 7, line 235-236)

" — a one-trial acquisition novel object-recognition task (NOR), performed on an L-maze and which tests episodic memory[49]",

Methods (page 18, lines 661-665)

Novel object recognition task (NOR)

One-trial-acquisition novel object recognition episodic memory was assayed as described previously[47] (see **Fig. 6a)** for a schematic representation). Our testing conditions represent a modification of the more ‘classical’ presentation of the test in that mice were tested on an L-maze rather than an open field or circular arena to reduce anxiety and to encourage exploration[93]

Discussion (page 10, lines 344-345)

“To this end, the use of the non-classical object recognition test (performed on an L-maze)…”

Comment:
Supplementary Figure 22 is stated as proving that no object bias existed for exploring the lego versus the bottle cap. However, the p values obtained in this simple t-test is p=.06, which is very close to statistical significance. Ns are only 4 per object. The authors are encouraged to expand this essential control experiment with larger Ns. If an innate preference for one object emerges, the authors are encouraged to choose different objects.

Response:
We thank the Reviewer for this suggestion. We have now expanded this analysis to include a sample size of 9 (see Figure 22 - Supplementary Figure). At the suggestion of a colleague (Dr Arnau Busquets-Garcia) who is familiar with this task (and first author of the publication in which this was used in the Fmr1KO model, Busquets-Garcia et al., Nature Medicine, 2013) we now present our data (exploration of each object) as a percentage of total time spent exploring, rather than as an absolute value. On the basis of this extended analysis, which is presented in the now modified Fig 22 – Supplementary Figure, we find no statistical difference between the exploration of the two objects ($P = 0.123$, $t = 1.63$, $df = 16$ by unpaired Student's t-test). This new analysis supports our previous finding, leading us to conclude that there is no bias for one object or the other. We hope that this new analysis will convince the Reviewer of the robustness of our findings.

Comment:
Differences between how the authors conducted fear conditioning, and how fear conditioning was conducted by the many other labs that did not detect fear conditioning deficits in Fmr1 knockout mice, must be clearly stated within the Discussion section.

Response:
We believe that the key difference between our work and previous findings in the field (with the exception of Oddi et al., 2015, Ding et al., 2014 and Gauducheau et al., 2017) is that we paired the shock with the presentation of the context. Previous studies have almost exclusively (with the exception of Eadie et al., Hippocampus, 2012) paired the shock (unconditioned stimulus) with a tone (conditioned stimulus). In this configuration, the conditioned stimulus (tone) becomes the main predictor of the aversive event and the context in which the experience took place assumes a background role (reviewed in Calandreau et al, J Neuroscience, 2006). It is therefore less surprising that the context in which the conditioned stimulus was presented becomes a less important predictor of cognitive defects. Likewise, it is likely that, as the context assumed a background role, it was not a sufficient predictor of cognitive deficits in the Fmr1KO model. We believe that these factors could explain the previous negative results, Using a purely contextual protocol there is a different relevance of the background versus foreground sensorial cues and

the related recruitment of the hippocampus (e.g. as described in Gerlai, R. (2013). Cued and contextual fear conditioning. Behavioral Genetics of the Mouse. S. Pietropaolo, F. Sluyter, R. Gerlai and W. E. Crusio. Cambridge, Cambridge University Press. 1: 315-324.). We propose that this modification may thus be represent an improvement for cognitive testing of the FMr1KO mouse.

We have discussed these issues in the modified Discusssion (page10 lines 341-354)

Comment:
Because the literature on lack of cognitive deficits in Fmr1 mice is a major source of controversy in the field, greatly increased emphasis on how the present behavioral assays differ from most of the standard literature on *Fmr1* mice is essential.

At present there is almost no description of the behavioral results in the Discussion section. The single sentence in lines 329-330 is entirely insufficient. If the authors want to state conclusions about cognitive deficits and rescues, relevant to the primary diagnostic symptom of Fragile X syndrome, considerably more text must be devoted to interpreting their behavioral findings appropriately.

Response:
We have now modified the Discussion to take these recommendations into account. The modfications are found on page 10, lines 341-354.

Comment:
If the authors are not as interested in the behavioral component, as indicated by a statement in the Response that behavioral characterization was not a major focus of the manuscript, then the behavioral figures should be removed entirely.

Response:
We appreciate the Reviewer's recommendations and have adapted the manuscript accordingly.

Reviewer #3 (Remarks to the Author):

Comment:
The authors have carefully considered the reviewers' comments, and the manuscript has been strengthened significantly. This is particularly true of the new behavior data and more thorough explanation. They should be commended for a well revised manuscript that will make an interesting addition to the literature.

Response:
We thank this reviewer for his/her statement that we have considered the reviewers' comments and provide a significantly strengthened and well-revised manuscript.

Reviewer #4 (Remarks to the Author):

Comment:
Their reported values for the diffusion coefficient of GluN1 are indeed a little higher than reported in comparable studies, but the authors provide plausible arguments for the difference in the measured diffusion coefficient. The precision and reproducibility of these experiments

depends greatly on the experimental conditions (type, age, species of culture); detection (antibody-labeled endogenous receptors, or exogenously tagged); methods used to track receptor (QDs or beads); acquisition parameters (frame rate etc.) and can be highly variable. But in fact, as they show in the appendix in the second rebuttal, the numbers are not terribly different. The skewed, non-Gaussian distributions in diffusion coefficients make that the median and mean values report very different aspects of the population, and I believe this might have caused some of the confusion. What is most important is that the experimental conditions within experiments are the same. Based on the presented information and data, the authors convincingly demonstrate that the lateral diffusion of mGluR5 and NR1 is altered in Fmr1 KO cultures, and that in particular mGluR5 diffusion is increased in "synaptic areas".

Response:
We thank the reviewer for this statement. The comparison table is now presented as part of the supplementary information (Supplementary Table).

Comment:
I do want to comment on the use of MitoTracker as a marker for synapses (as was noted by reviewer 2). Although MitoTracker has "extensively been used", at best it is a very rough estimate of synaptic sites; 60-70% overlap with synaptotagmin according to Groc et al., 2004, meaning that in 30-40% of the cases, synaptic tracks are not correctly assigned. This most likely reflects the fact that the majority of spines does not contain mitochondria (see e.g. Li et al., Cell 2004). Moreover, the morphology of mitochondria is highly variable and does not relate at all to synapse shape. And, important for this study, mobility, location, shape and number of mitochondria are all influenced by neuronal activity. So, even if MitoTracker was the best possible approach for estimating synaptic location in these experiments, the authors should at least demonstrate that the ability of MitoTracker to label synaptic sites is similar between wild-type and Fmr1 KO cultures, in which the activity of synapses is severely altered.

Response:
We thank the reviewer for pointing out this possible caveat in our experiments. We addressed this criticism by performing double and triple labeling experiments in hippocampal cultures from WT and Fmr1 KO mice. We labeled synaptic sites by MitoTracker, presynaptic sites using antibodies against the presynaptic marker bassoon, and postsynaptic sites by using antibodies against Homer. We then quantified the overlap of MitoTracker with these other synaptic markers and tested for significant genotype differences. The results from this analysis are now presented as Supplementary Figure1 and discussed in the text (Results; page 4, 115-116 description of method, Methods page13-14, lines 494-515), and demonstrate that there is no significant difference between WT and Fmr1KO neurons in the ability of MitoTracker to detect synapses.

<u>REVIEWERS' COMMENTS:</u>

Reviewer #2 (Remarks to the Author):

The authors have successfully addressed the previous issues in the behavioral components of the manuscript.

Reviewer #4 (Remarks to the Author):

The authors have adequately addressed my concern.
